# Controlling the Symmetry of Perylene Derivatives via Selective *ortho*-Borylation

**DOI:** 10.1021/acs.joc.4c02669

**Published:** 2025-02-27

**Authors:** David Sánchez-Fernández, Tomás Torres, José García-Calvo

**Affiliations:** † Department of Organic Chemistry, 16722 Universidad Autónoma de Madrid, Campus de Cantoblanco, 28049 Madrid, Spain; ‡ Institute for Advanced Research in Chemical Sciences (IAdChem), Universidad Autónoma de Madrid, Campus de Cantoblanco, 28049 Madrid, Spain; § IMDEA-Nanociencia, c/Faraday 9, Campus de Cantoblanco, 28049 Madrid, Spain

## Abstract

This work presents a systematic and rational approach to the synthesis of previously reported as well as novel tetra- and di-*ortho*-borylated perylene, perylenediimide, and perylenemonoimide scaffolds. Through optimization of the reaction conditions, employing [Ir­(OMe)­(COD)]_2_ as a catalyst and suitable ligands, efficient tetraborylation and regioselective diborylations were achieved. Additionally, the reaction times were reduced from days to hours under microwave irradiation, rendering this methodology a practical and scalable route for the *ortho*-functionalization of perylene derivatives.

## Graphical abstract

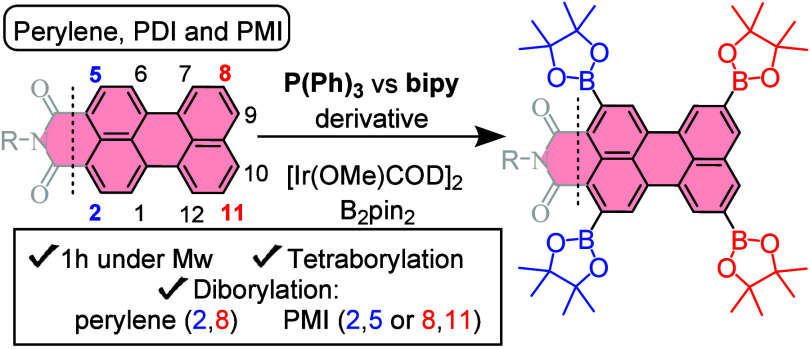

## Introduction

Perylene derivatives (PDs), including the widely explored peryleneimides (PI), are known for their scalable synthesis, high thermo- and photochemical stability,^1–3^ outstanding physicochemical properties, and, as a consequence, by their applications in materials chemistry and optoelectronics.^1–5^ However, perylene and PIs also exhibit some key differences in symmetry or charge distribution, providing unique applications to each derivative. Synthetically, PDs possess three differentiated positions for modification (see Figure 1), namely, *ortho*, *pery* (i.e., by the introduction of different substituents into the imide group), or *bay*, widely studied in the derivatization of PIs.^1,5^ With a focus on *ortho-*substituted PDs (positions 2, 5, 8, and 11 of the perylene core), their synthesis has been tackled by using ruthenium catalysts for C–C bond formation with aryl halides or alkenes,^6,7^ by using a rhodium catalyst to introduce alkyne derivatives^8^ or for direct halogenation,^9^ or even by the straightforward reaction with aryl magnesium derivatives by electrophilic substitution.^4^ In comparison to those approaches, this study concentrates on borylation, because borylated PDs may undergo C–C Suzuki couplings, and on substitutions to amino,^10^ cyano,^11^ or hydroxy^12^ groups, beyond just halogens.^13^

**Figure 1:**
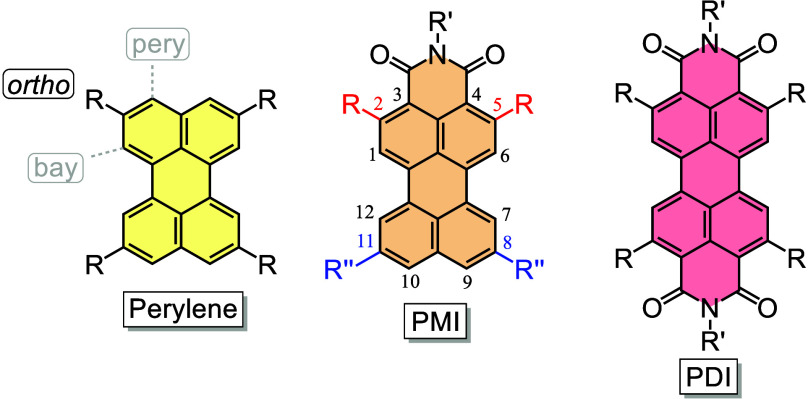
Structures of perylene, PMI, and PDI derivatives showing the *ortho* positions studied in this work (R and R″).

Selective borylation on activated C–H bonds is also a hot topic in a vast number of research lines,^14,15^ and the catalyst [Ir­(OMe)­COD]_2_ has been extensively studied in conjunction with bis­(pinacolato)­diboron (B_2_pin_2_) and diverse ligands.^16^ Ligand modification offers a versatile strategy for improving reactivity and/or selectivity, by modulating bulkiness and electronic properties.^17^ For instance, bidentate ligands such as 4,4′-di-*tert*-butyl-2,2′-bipyridine (dtbpy) enabled selective C–H activations,^14,18,19^ preferentially in unhindered positions^20^ [see the *ortho* positions of perylene (Figure 1)]. In contrast, monodentate phenyl phosphine derivatives have been demonstrated to give better results for the C–H activation and borylation reaction in aromatic rings bearing a carbonyl substituent in the *ortho* position.^21,22^ With regard to PDs, [Ir­(OMe)­COD]_2_-catalyzed borylations were first reported for the tetrasubstitution of perylene^23^ and PDIs.^12^ Moreover, via modification of the reaction conditions, less borylated species have also been synthesized.^24^ Conversely, although these methods have proven to be effective for perylenes and PDIs, their applicability to the less studied PMIs^25,26^ remains mostly unexplored. In that regard, PMIs possess only one imide group, differentiating positions 2 and 5, used for selective arylation or alkylation with a ruthenium catalyst,^7,27–29^ from positions 8 and 11, used for selective [Ir­(OMe)­COD]_2_ borylation.^30^

Altogether, the chemistry of PDs would benefit from a systematic approach for obtaining *ortho*-substituted perylene derivatives by an efficient and reproducible procedure. Therefore, building upon the existing research, this study aimed (1) to explore a small number of commercial ligands to optimize the synthesis of *o*-boronic esters of perylene and PDIs, (2) to apply the better-performing ligands for potential regioselective borylation of PMIs, and (3) to test the results under microwave (Mw)-assisted conditions, potentially reducing the reaction times from days to minutes.^31^

## Results and Discussion

### Synthesis of *ortho*-Borylated PDs

Perylene (**1′**) was used as received, while PDI (**2′**) and PMI (**3′**) were obtained simultaneously starting from commercially acquired perylene dianhydride (PDA) and 6-aminoundecane, respectively, following the procedure described in ref 25. Next, the conditions for obtaining the perylene^23^ and PDI^12^ tetra-*o*-boronic esters were adapted from the reported reactions with [Ir­(OMe)­COD]_2_ as the catalyst. For perylene, the best results were obtained using the dtbpy ligand, which is bidentate and rich in electrons, whereas for PDI, monodentate ligand P­(C_6_F_5_)_3_ was used. Those conditions were in agreement with the previously demonstrated involvement of carbonyl groups in the *ortho* position^32^ and the role of sterics and/or electronic features in the C–H activation.^32–34^ However, while ligand modification has proven to be useful and yields of >80% have been reached for dtbpy (in perylene) or for P­(C_6_F_5_)_3_ (in PDIs), further research may provide new insights into the potential of phosphine ligands for perylene borylation or the impact of using bipyridine ligands on PDIs.

Consequently, procedures from the literature were adapted (see Schemes 1 and 2 and section 2 of the Supporting Information).^10^ On one hand, perylene, B_2_(pin)_2_ (8 equiv), the [Ir­(OMe)­COD]_2_ catalyst (5% mol), and di-*tert-*butyl-bipyridine (dtbpy, **L3**, 10% mol) were mixed in hot cyclohexane and refluxed for 72 h. After the reaction had reached completion, the mixture was cooled and filtered under a vacuum to afford pure tetraborylated perylene [**1a**, 86% (Scheme 1)]. On the other hand, a very similar methodology is reported for the tetraborylation of PDIs,^12^ but employing a monodentate ligand [P­(C_6_F_5_)_3_, **L5**] and dioxane as the solvent, to afford PDI **2a** after purification by column chromatography [83% (Scheme 2)].

**Scheme 1:**
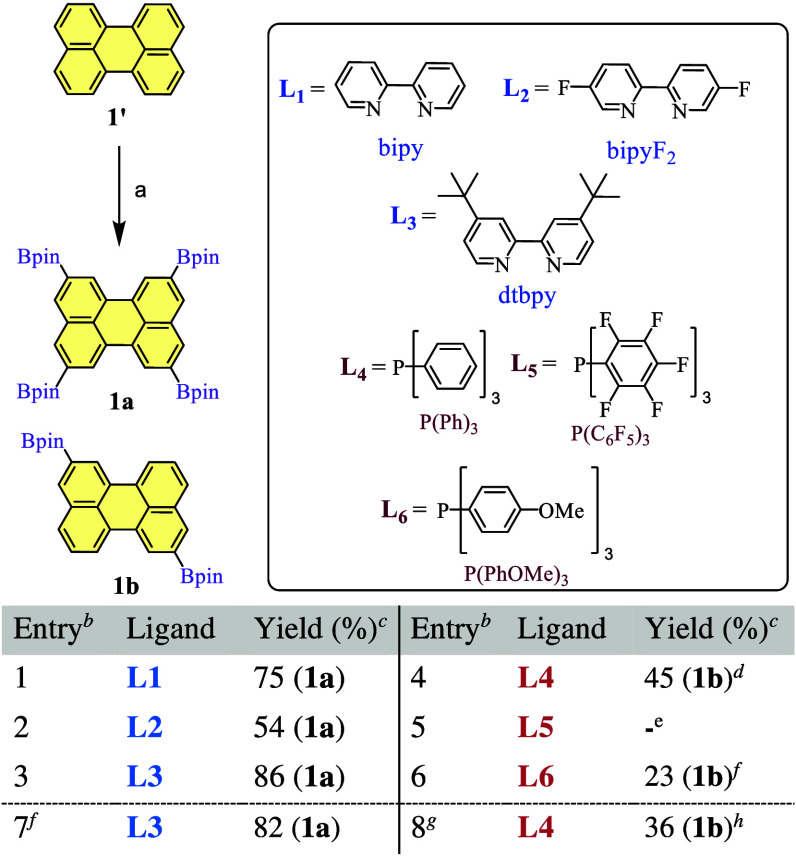
Reaction Conditions and Ligands Explored for the Borylation of Perylene (**1′**) to Obtain Products **1a** and **1b**

a Perylene (**1′**) (0.1 mmol), B_2_pin_2_ (0.8 mmol), [Ir­(OMe)­(COD)]_2_ (5% mol), a ligand (10% mol), cyclohexane (20 mM), 72 h, 90 °C.
b Entries 1–8.
c Yields are calculated from isolated products.
d Conversion of 60%.
e No conversion.
f Conversion of 30%.
g Same conditions but heating in the Mw for 1 h at 150 °C.
h Conversion of 52%.

**Scheme 2:**
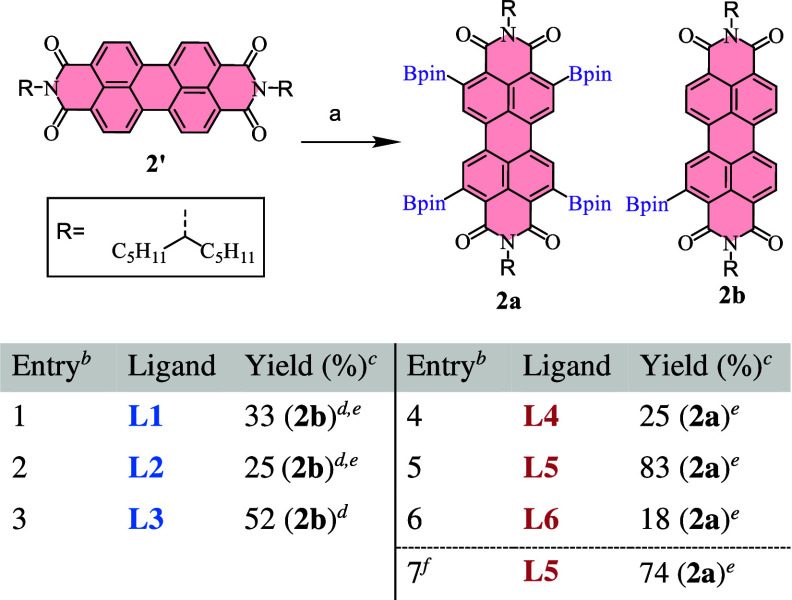
Reaction Conditions and Ligands Explored for the Borylation of PDI (**2′**) to Obtain Products **2a** and **2b**

a PDI (**2′**) (0.1 mmol), B_2_pin_2_ (0.8 mmol), [Ir­(OMe)­(COD)]_2_ (5% mol), a ligand (10% mol), dioxane (20 mM), 72 h, 120 °C.
b Entries 1–7.
c Yields are calculated from isolated products.
d Full conversion was not achieved.
e Mixture of different borylations isolating the major product.
f Same conditions but heating in the Mw for 1 h at 150 °C.

The same borylation conditions were also tested with commercially available and more electron rich phosphine derivatives (**L4** and **L6**), along with more electron poor bipyridine ligands (**L1** and **L2**), affording the results summarized in Schemes 1 and 2.

When the results for the borylation of perylene were analyzed [**1′** (Scheme 1)], it was observed that all ligands but **L5** afforded conversion to borylated products under the tested conditions. Using 2,2′-bipyridine (**L1**) led us to results very similar to those of the reported dtbpy ligand (**L3**), but with slightly lower yields (75%), from which a small quantity of the less borylated products was formed and column chromatography was needed to isolate **1a**. In comparison, the difluorinated version (**L2**) led again to a mixture, but even more complicated, in which **1a** represented only 54%. The results for the phosphine derivatives were again in line with a dependence on the electronic features, because no reaction was observed whenever the pentafluorinated phosphine was used (**L5**). However, after several tests employing phosphine **L6** (**1b**, 23%) and, in particular, triphenylphosphine **L4** (**1b**, 45%), conversions of 32–60% were achieved. While these conversions might appear to be a drawback, these conditions actually favored the incomplete borylation to disubstituted perylene **1b** over further borylated species. Additionally, precipitation of **1b** in a 1:1 heptane/cyclohexane mixture and subsequent washing with heptane and methanol, a method that has proven to be effective for the selective isolation of borylated species,^35^ facilitated the separation of this product. Finally, with the aim of improving the reported procedures, experiments were conducted employing the best performing ligands (**L3** and **L4**) and repeating the reaction under microwave-assisted heating at 150 °C (Scheme 1, entries 7 and 8), and borylated products were attained in analogous yields (entries 7 and 8).

The borylation of PDI **2′** (Scheme 2) proceeded mostly as anticipated from previous research. The use of the dtbpy ligand (**L3**) did not provide full conversions, affording only monoborylated product **2b** in 52% yield. However, this yield was surprisingly higher than that of the previously reported monoborylation with the same ligand (13%),^12^ which used a shorter alkyl chain in the imide group. Reactions with bypiridines **L1** and **L3** resulted in mixtures of di- and monoborylated products in moderate yields, ranging from 20% to 35%. In sharp contrast, whereas the use of fluorinated phosphine **L5** led to the nearly complete conversion to tetraborylated product **2a** (83% heating in a plate, 74% under Mw), ligands **L4** and **L6** produced again complicated mixtures containing varying amounts of tetra-, tri-, di-, and monoborylated PDIs. Hence, the use of **L4** and **L6** was rendered impractical for this reaction due to the significant challenges associated with purification, which would necessitate multiple chromatographic separations and excessive solvent consumption. All of those results indicated that the imide substituent also played a vital role in the borylation, likely because of the solubility and aggregation issues of PDIs. Long aliphatic chains seemed to improve the reactivity under the same reaction conditions, a fact already observed in tetraborylation with P­(C_6_F_5_)_3_,^12^ which explains the high yields for the monoborylation with dtbpy. Therefore, choosing the more suitable conditions may not imply the use of the P­(C_6_F_5_)_3_ ligand, because dtbpy may lead to better results for monoborylation, leading to easier purifications and fewer undesired products.

Building upon the ligand screening results, we also sought to optimize the formation of boronic esters in PMIs, selecting the monodentate and bidentate ligands with higher conversions, P­(C_6_F_5_)_3_ (**L5**) and dtbpy (**L3**), respectively, for further studies. A series of experiments were conducted to identify the ideal solvent, temperature, and reaction times for the synthesis, as summarized in Scheme 3. Early investigations focused on the dtbpy ligand (**L3**) in dioxane and THF. While refluxing the reaction mixtures in THF by heating in a plate (Scheme 3, entry 2) proved to be ineffective, optimal conditions for obtaining diborylated product **3b** involved refluxing in dioxane for 3 days (55%) or Mw heating in THF well above its boiling point, which yielded **3b** selectively and nearly quantitatively (88%). Monodentate ligand **L5** was also used under conditions based on literature precedents,^8,28^ and complete conversion was achieved by refluxing in dioxane for 72 h (76%) or, again, by Mw heating at 110 °C in THF for 1 h (71%), affording *ortho*-diborylated product **3c** (with opposite regioselectivity to **3b**). Notably, all of the diborylation reactions were sensitive to temperature, with no conversion observed when working below reflux temperatures. Finally, to pursue the challenging tetraborylation, a one-pot, two-step approach was implemented. Given the 4:1 ligand:catalyst ratio, the possibility of starting with **L3** or **L5** was evaluated. On one hand, using first **L3** led exclusively to product **3b**, suggesting a higher affinity of the iridium catalyst for **L3**. On the other hand, the sequential addition of **L5** followed by **L3**, and the corresponding additional catalyst, successfully afforded tetraborylated product **3a**, by heating in a plate (62%) or in the Mw (65%) (Scheme 2, entries 11 and 12).

**Scheme 3:**
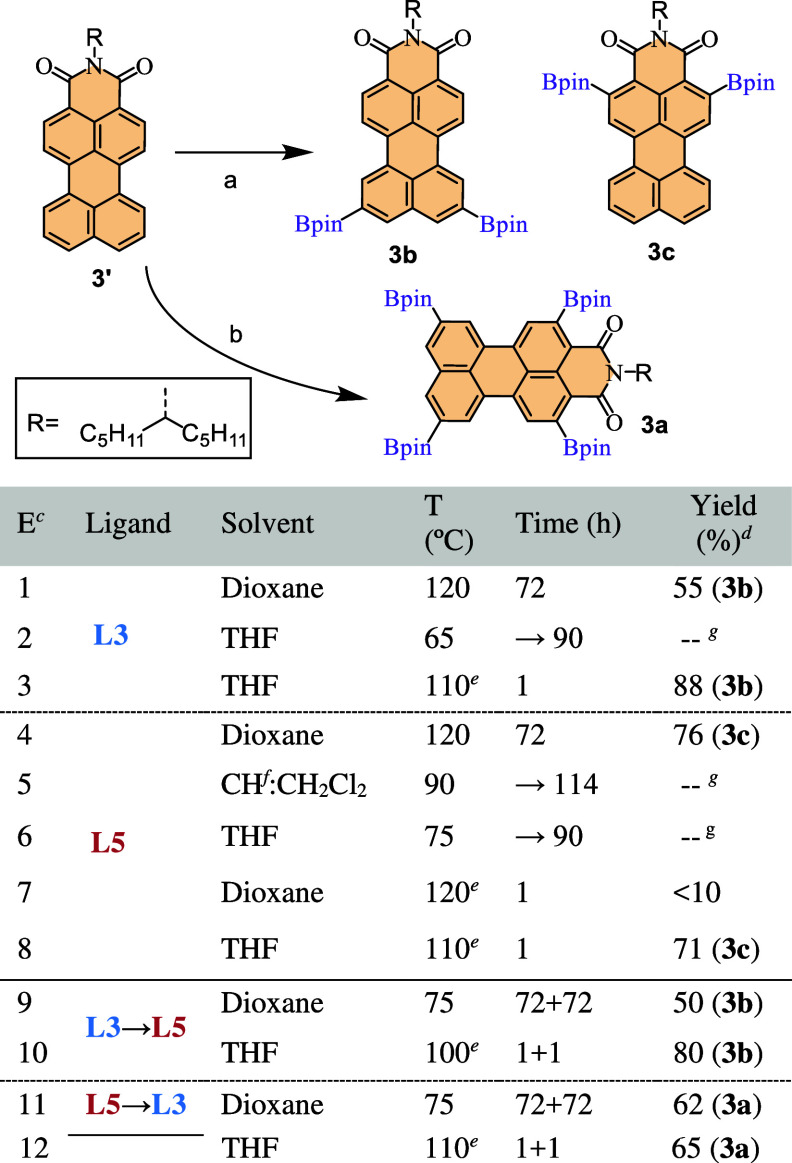
Reaction Conditions Tested for the Reaction of PMI **3′** to Obtain Products **3a–3c** in One (entries 1–8) or Two Steps (entries 9–12) via a One-Pot Reaction^a^

a PMI (**3′**) (0.1 mmol), B_2_Pin_2_ (0.8 mmol), [Ir­(OMe)­(COD)]_2_ (5% mol), a ligand (10% mol), 20 mM, **L3** or **L5**, a solvent, temperature, time.
b Same conditions but with a second step: [Ir­(OMe)­(COD)]_2_ (5% mol), a ligand (10% mol), time, temperature.
c Entry.
d Yields are calculated from isolated products.
e Mw irradiation.
f CH, cyclohexane.
g No reaction. PMI **3′** was recovered.
h Solvents, temperatures, and reaction times were tested as specified.

### Photophysical Properties and Electronic Structure

The photophysical properties of PDs might be tuned by not only the number substituents but also attending to their distribution. Thus, the absorption and fluorescence spectra of borylated PDs **1a**, **2a**, **3a**, **3b**, and **3c** were recorded in chloroform (Figure 2a,b). The results were compared with those of their unsubstituted homologues **1′**–**3′** (Figure 2b–d), and the absorption spectra were deconvoluted (Figures S19, S21, and S23) to obtain a more accurate assignment of the bands and the maxima. Density functional theory (DFT) calculations were also performed for a comprehensive analysis of the HOMO–LUMO variations, as well as time-dependent DFT (TD-DFT) calculations (Figures S20, S22, and S24), in order to compare the theoretical changes in the UV-Vis absorption with the experimental values.

**Figure 2:**
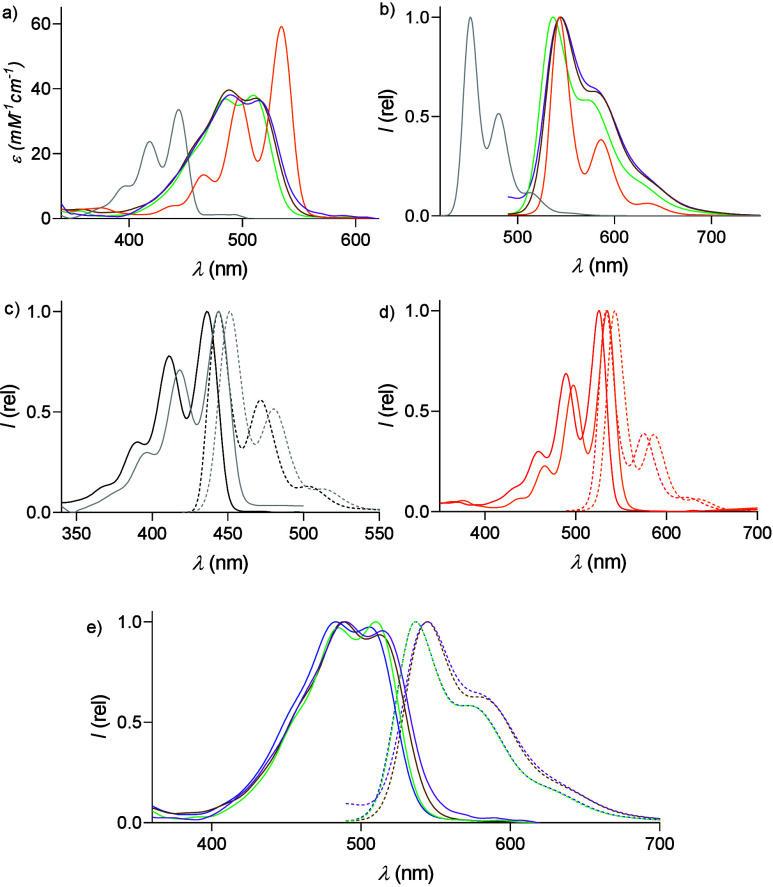
(a) ε vs λ of **1a**, **2a**, **3a**, **3b**, and **3c**. (b) Normalized emission spectra of **1a**, **2a**, **3a**, **3b**, and **3c**. (c) Normalized absorbance (solid lines) and emission (dashed line; λ_ex_ = 410 nm) of **1′** and **1a**. (d) Normalized absorbance (solid lines) and emission (dashed line; λ_ex_ = 480 nm) of **2′** and **2a**. (e) Normalized absorbance (plain) and emission (dashed line; λ_ex_ = 480 nm) of **3′**, **3a**, **3b**, and **3c**. Dissolved in chloroform were **1′** (black), **1a** (gray), **2′** (red), **2a** (orange), **3′** (brown), **3a** (purple), **3b** (brown), and **3c** (green).

According to the literature,^24,36^ the substitution of the hydrogens in *ortho* positions to boronic esters has little influence over the absorption or fluorescence spectra in solution, while there is a clear change in shape and position among perylene, PDI, and PMI. In comparison to perylene **1′** and PDI **2′**, tetraborylated **1a** and **2a** displayed red-shifted absorption (+8 and +9 nm, respectively), while from PMI **3′**, an only +1 nm shift was observed for **3a** (see Table 1). By deconvolution of the vibronic states into Gaussian components (Figures S19, S21, and S23), it was observed that borylated perylene **1a** and PDI **2a** produced no significant changes in the shapes, sizes, or proportions of the components, while PMI’s fine structure depends on the position of the substituents (see Figure 2e); we note the importance of selective changes over the electronic distribution. For the emission spectra, the same trend was followed, with slightly red-shifted bands upon borylation of the PMI in all of the cases except **3c**, which remained unchanged. The values of the molar extinction coefficients (Figure 2a and Table 1) are in agreement with the literature, displaying similar values for perylenes and PMIs while being slightly higher for PDIs. The fluorescence quantum yields (Φ_F_) showed not significant changes for perylenes and PDIs,^13^ in contrast to the sharp decrease reported in the literature for the tetraborylated species of the former^24^ (0.93 in chloroform vs 0.58 in toluene). Still, the Φ_F_ values for PMIs **3′** (79%), **3a** (70%), **3b** (68%), and **3c** (83%) depicted a slightly reduced value whenever the boronic ester was in the opposite position to the imide (**3a** and **3b**).

**Table 1:** Parameters Calculated for the Compounds of This Study

compound	ε^b^ (mM^–1^ cm^–1^)	λ_abs_ ^c^ (nm)	λ_em_ ^d^ (nm)	Φ_F_ [Table-fn t1fn5] (%)
**1′**	41	436	429	0.95
**1a**	34.6	444	451	0.93
**2′**	68.2	526	534	0.98
**2a**	60.2	535	543	0.85
**3′**	37.3	513	537	0.79
**3a**	38.1	514	544	0.70
**3b**	39.7	517	544	0.68
**3c**	35.1	513	537	0.83

a Molar extinction coefficient (ε) at the absorption maxima.

b Position of the absorption maxima with the highest energy.

c Position of the emission maxima with the highest energy.

d Fluorescence quantum yield in chloroform.

To support our findings and better understand the electronic changes in the structure, DFT calculations were performed by applying the B3LyP functional at the 6-31G­(d,p) level and supplemented by calculations of the electrostatic potentials (Figures S15–S17). DFT calculations of the HOMO–LUMO are represented for PMI in Figure 3 and are expanded in Figure S18. In summary, the energy for the HOMO–LUMO levels increases whenever any of the products are borylated and the gap becomes slightly lower, what matches with the small red-shifts in absorption. Noticeable changes are observed in the LUMO+1 of **3a** or **3b** in comparison to that of unsubstituted **3′**. However, the effect at the HOMO level is similar in all of the borylated products because changes are observed only whenever further energy levels are considered, such as HOMO–2 (Figures S11–S14) for **3b**, which is totally different from **3′**, suggesting again the potential tunability of this kind of PMI derivative by selective derivatization at the *ortho* positions. TD-DFT calculations were also performed at the CAM-B3LyP level (Figures S20, S22, and S24), where the absorption over 350 nm was explained (>95%) as HOMO → LUMO transitions (S_0_ → S_1_), foreshadowing the positions of the absorption bands and their intensities accurately with respect to the experimentally obtained molar extinction coefficients.

**Figure 3:**
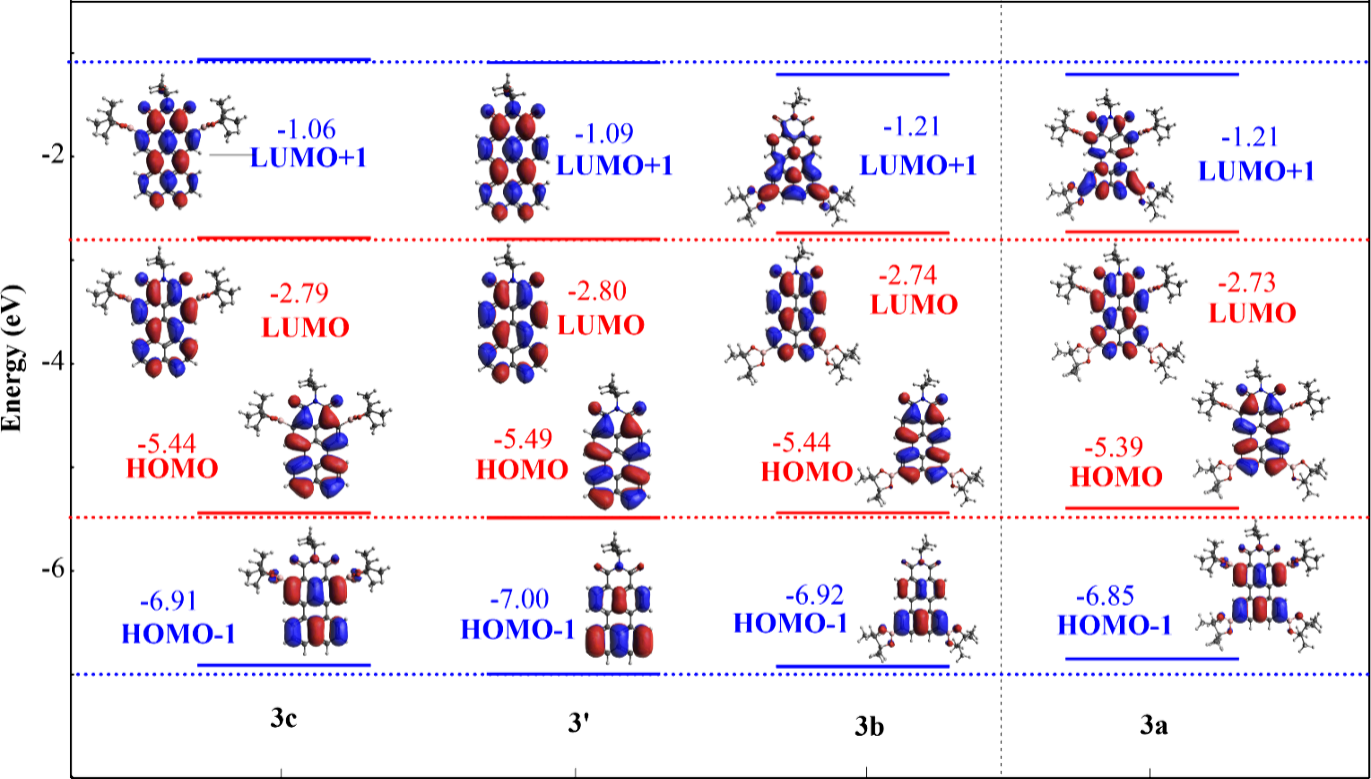
Distribution of the calculated energy levels for the HOMO – LUMO and HOMO–1 – LUMO+1 and their corresponding MO distribution for compounds **3a**–**3c** compared to those of PMI starting material **3′**.

## Conclusions

The results of this study represent the groundwork for expanding the chemistry of *ortho*-functionalized perylenes. A systematic approach to the synthesis of *ortho*-borylated perylene derivatives was performed, culminating in optimized protocols for the preparation of tetraborylated perylene and PDI in <1 h, together with a convenient approach for the synthesis and purification of 2,8-bis­(4,4,5,5-tetramethyl-1,3,2-dioxaborolan-2-yl)­perylene. The synthesis and characterization of tetra-*ortho*-borylated and diborylated PMIs were also described, with regiocontrol over the boronic ester positions relative to the imide. With regard to [Ir­(OMe)­COD]_2_ catalysis, it was confirmed that the imide substituents may strongly affect the borylation conditions, especially when looking for less borylated species such as the monoborylated PDI. In addition, the influence of the ligands was confirmed to play a critical role, too; the use of the bidentate ligand dtbpy favored tetraborylation of the less hindered *ortho* positions of PMI or perylene, while P­(C_6_F_5_)_3_ promoted borylation in *ortho* to the imide groups for both PDI and PMI. Additionally, choosing the different ligands might be of utmost importance when looking for the less borylated compounds. Finally, DFT calculations and ultraviolet–visible (UV–vis)/fluorescence spectroscopy supported the hypothesis that selective *ortho* substitution is a promising strategy for modulating perylene HOMO–LUMO energy levels and, therefore, optoelectronic properties.

## Supplementary Material





## Data Availability

The data underlying this study are available in the published article and its . The raw data of the UV–vis and fluorescence spectra and the HRMS are openly available at the Institutional Repository of the UAM at 10.21950/CVCYKL.

## Notes

FAIR data, including the primary NMR FID files, for compounds **1a**, **1b**, **2a**, **2b**, **3a**, **3b**, and **3c** (ZIP)
Experimental procedures and characterization (PDF)
