# Supplementary material for: Controlling the Symmetry of Perylene Derivatives via Selective ortho-Borylation
Source: J Org Chem. 2025 Feb 27;90(9):3202–8. doi: 10.1021/acs.joc.4c02669 (PMC12131211; doi:10.1021/acs.joc.4c02669)
Supplement: Supplementary file 2 [file jo4c02669_si_002.pdf]

Supporting Information

Controlling the Symmetry of Perylene Derivatives via Selective *Ortho*-Borylation

David Sánchez-Fernández, [a] Tomás Torres,*[a,b,c] José García-Calvo*[a,b,c]

[a]*Department of Organic Chemistry, Facultad de Ciencias, Universidad de Madrid Autónoma Cantoblanco, 28049-Madrid, Spain*

[b]*Institute for Advanced Research in Chemical Sciences (IAdChem), Universidad Autónoma de Madrid, Campus de Cantoblanco, 28049 Madrid, Spain*

[c]*IMDEA-Nanociencia, c/Faraday 9, Campus de Cantoblanco, 28049 Madrid, Spain*

jose.garciac@uam.es

tomas.torres@uam.es

Table of Contents

S1. EXPERIMENTAL SECTION 3

S1.1. Materials and Methods 3

S1.2. Synthesis and Characterization 4

S.1.2.1. Synthesis of *ortho*-borylated Perylene (**1a** and **1b**) 4

S.1.2.2. Synthesis of *ortho*-borylated PDIs (**2a** and **2b**) 6

S.1.2.3. Synthesis of *ortho*-borylated PMIs (**3a**, **3b** and **3c**) 8

S.1.3. ^{1}H NMR Spectra Comparison and Temperature Dependence 11

S.2. COMPUTATIONAL STUDIES 12

S.2.1. Cartesian Coordinates 19

S.3. OPTOELECTRONIC MEASUREMENTS 49

S.4. NMR CHARACTERIZATION 54

S.5. HRMS CHARACTERIZATION 65

S.6. SUPPORTING REFERENCES 67

S1. EXPERIMENTAL SECTION

S1.1. Materials and Methods

Chemical reagents and solvents were purchased from Merck-Sigma Aldrich, BLD-pharmaceuticals, TCI and Across and were used without further purification. Monitoring of the reactions has been carried out by thin layer chromatography (TLC), employing aluminium sheets coated with silica gel type 60 F254 (0.2 mm thick, E. Merck). Purification and separation of the synthesized products was performed by column chromatography, using silica gel (230–400 mesh, 0.040- 0.063 mm, Merck). When the Schlenks or flask were heated in a plate, aluminium adaptors with the proper size or a sand bath were employed, in all cases with a temperature controller display.

Mass Spectrometry (MS) and High-Resolution Mass Spectrometry (HRMS) spectra were recorded employing Electrospray Ionization (ESI Positive TOF_MS) mass spectra using a MAXIS II spectrometer, or Matrix Assisted Laser Desorption/Ionization-Time of Flight (MALDI-TOF) using a Bruker Ultraflex III TOF/TOF spectrometer, with a nitrogen laser operating at 337 nm, or with a NdYAG laser operating at 335 nm. The different matrixes employed are indicated for each spectrum. Mass spectrometry data are expressed in m/z units. All MS experiments were carried out at the "Servicio Interdepartamental de Investigación" (SIdI) of the Universidad Autónoma de Madrid. ^{1}H NMR and ^{13}C NMR were recorded on Bruker XRD-300 (300 MHz) and/or Bruker XRD-500 (500 MHz) instruments at room temperature (25 °C) and are reported as chemical shifts (δ) in ppm relative to TMS (δ = 0). Spin multiplicities are reported as a singlet (s), doublet (d), triplet (t) and quartet (q) with coupling constants (*J*) given in Hz, or multiplet (m). Broad peaks are marked as br. ^{1}H and ^{13}C resonances were assigned with the aid of additional information from 1D and 2D NMR spectra (H,H-NOESY, H,H-COSY, DEPT 135, HSQC and HMBC). Deuterated solvents employed are indicated in each spectrum. Structural assignments were made with additional information from gCOSY, gHSQC, and gHMBC experiments. IR spectra were recorded on an Agilent Technology Cary 630 FT-IR spectrometer (ATR) and are reported as wavenumbers υ in cm^{-1} with band intensities indicated as s (strong), m (medium), w (weak), br (broad). Spectroscopic and photophysical characterization UV-vis spectra were recorded on a JASCO-V660 UV-vis spectrophotometer using spectroscopic grade solvents and 10x10mm quartz cuvettes with a Jasco Peltier ETCS-761 temperature controller incorporated, or in a double beam UV-Vis-NIR Varian Cary 6000i spectrophotometer (Varian, Palo Alto, CA, USA). Fluorescence spectra were recorded with a JASCO FP-8600 spectrophotometer using spectroscopic grade solvents and quartz cuvettes (1cm) with a Jasco Peltier ETCS-761 temperature controller incorporated, or in a Spex Fluoromax-4 spectrofluorometer (Horiba Jobin-

Yvon, Edison, NJ, USA) equipped with a stirrer and a temperature controller. All Fluorescence spectra were corrected for lamp intensity fluctuations, background, and the wavelength-dependent response function of the detector.

Abbreviations: 1,4-Diazabicyclo[2.2.2]octane (DABCO); 4,4′-Di-tert-butyl-2,2′-dipyridyl (dtbpy), 3,3′-difluoro-2,2′-dipyridyl (bipy), 5,5'-difluoro-2,2'-bipyridine (bipyF2), microwave (Mw), perylene derivatives (PD), perylene dianhydride (PDA), perylene monoimide (PMI), perylene diimide (PDI).

S1.2. Synthesis and Characterization

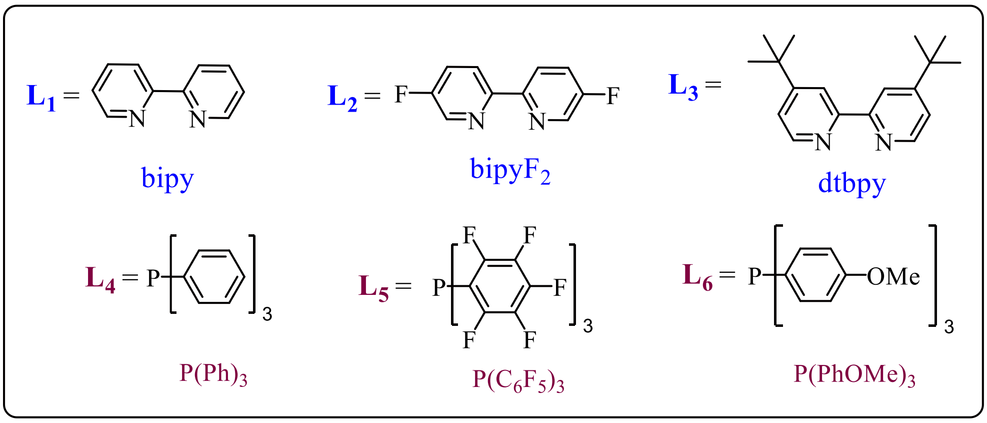

Figure S1 Structures of the ligands used in this study **L_1 – L_6**.

S.1.2.1. Synthesis of *ortho*-borylated Perylene (1a and 1b)

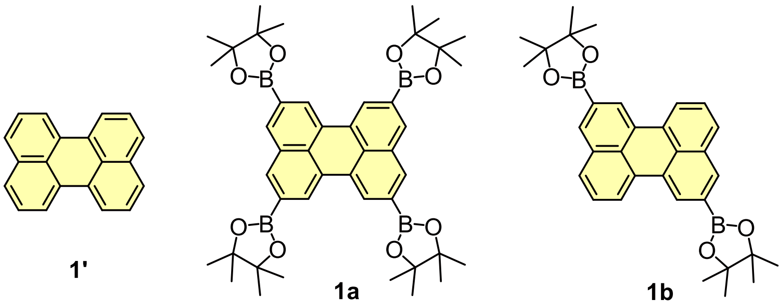

Figure S2 Structures of the perylene derivatives from this study. Perylene (**1'**), 2,5,8,11-tetrakis(4,4,5,5-tetramethyl-1,3,2-dioxaborolan-2-yl)perylene (**1a**) and 2,8-bis(4,4,5,5-tetramethyl-1,3,2-dioxaborolan-2-yl)perylene (**1b**).

Borylation of perylene (1') was adapted from the reported procedure.[S1]

Method 1: In a 25 mL Schlenk, perylene (50 mg, 0.2 mmol), B_2pin_2 (400 mg, 1.6 mmol), $[Ir(OMe)(cod)]_2$ (6.6 mg, 0.01 mmol) and ligand (L) (0.02 mmol) were introduced under Ar atmosphere, and mixed in dry cyclohexane (4 mL). The mixture was stirred in a plate under reflux (100 ºC) for 72h and cooled to rt. Once at rt the precipitate was filtered and washed with hot cyclohexane (50 ºC, 15 mL) and MeOH (10 mL) to obtain the corresponding perylene as a yellow powder.

L1 (3.1 mg). Product obtained: **1a** (112 mg, 75%).[a]

L2 (3.8 mg). Product obtained: **1a** (81 mg, 54%).[a]

L3 (5.4 mg). Product obtained: **1a** (129 mg, 86%).[a]

L4 (5.2 mg). Product obtained: **1b** (45 mg, 45%), (19 mg, 60% conversion).[b]

L5 (10.6mg). The starting material was recovered.

L6 (7.0 mg). Product obtained: **1b** (23 mg, 23%), **1'** (35 mg, 30% conversion).[b]

Method 2: In a 5 mL microwave vial, perylene (50 mg, 0.2 mmol), B_2pin_2 (400 mg, 1.6 mmol), $[Ir(OMe)(cod)]_2$ (6.6 mg, 0.01 mmol) and ligand (L) (0.02 mmol) were put in Ar atmosphere, and dry cyclohexane (4 mL) was added. The mixture was heated in the microwave at 150 ºC for 1h. After cooling, the crude was filtered and washed with hot cyclohexane (15 mL) and MeOH (10 mL) to obtain the corresponding perylene derivative as a yellow powder.

L3 (5.4 mg). Product obtained: **1a** (123 mg, 82%).[a]

L4 (5.2 mg). Product obtained: **1b** (36 mg, 36%). (26 mg, 52% conversion).[b]

[a]Full conversion was reached, obtaining a mixture of the lesser borylated products in the crude after filtration.

[b]**1b** was isolated by precipitating in CH/Heptane 1:1, washed with heptane (20 mL) and then with MeOH (10 mL). The filtered crude was concentrated under vacuum and purified by SiO_2 column chromatography (Heptane/CH_2Cl_2 1:1 to CH_2Cl_2) to recover the unreacted perylene.

Product **1a**: **^{1}H NMR** (300 MHz, $CDCl_3$): 8.63 (s, 4H); 8.25 (s, 4H); 1.43 (s, 48H). **^{13}C{1H} NMR** (75 MHz, $CDCl_3$): 137.1 (CH), 133.4 (C), 132.1 (C), 130.5 (C), 126.2 (CH), 84.2 (C), 25.1 (CH_3). **IR**

(ATR): 3047 (m), 2976 (s), 2929 (m), 1612 (w), 1489 (s), 1388 (s), 1379 (s), 1267 (s), 1205 (m), 1165 (s), 966 (s), 852 (s), 686 (s).

Product **1b**: ^{1}**H NMR** (300 MHz, $CDCl_3$): 8.54 (s, 2H), 8.36 (d, $^{3}J_{H-H}$ = 7.3 Hz, 2H), 8.18 (s, 2H), 7.71 (d, $^{3}J_{H-H}$ = 8.1 Hz, 2H), 7.50 – 7.45 (m, 2H), 1.42 (s, 24H). ^{13}**C{1H} NMR** (75 MHz, $CDCl_3$): 136.1 (CH), 134.2 (C), 131.4 (C), 130.5 (C), 128.5 (CH), 126.6 (CH), 125.1 (CH), 121.7 (CH), 84.2 (C), 25.1 (CH_3). **IR (ATR):** 3039 (m), 2975 (s), 2928 (m), 1612 (w), 1597 (w), 1472 (s), 1429 (s), 1390 (s), 1370 (s), 1311 (m), 1272 (s), 1214 (s), 1200 (s), 1006 (s), 757 (s), 639 (s).

S.1.2.2. Synthesis of *ortho*-borylated PDIs (2a and 2b)

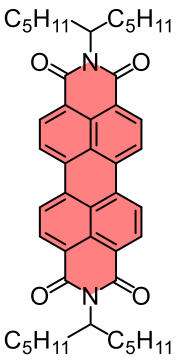

2'

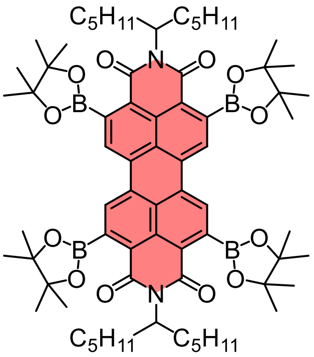

2a

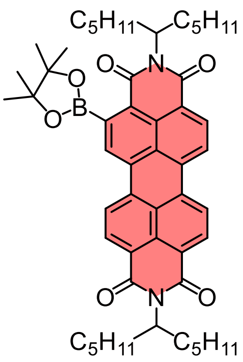

2b

Figure S3 Structures of the PDI derivatives from this study. PDI (**2'**), its tetra- *ortho*-borylated derivative (**2a**) and mono-borylated (**2b**).

Borylation of PDI (2') was adapted from the reported procedure.[S2]

Method 1: In a 25 mL Schlenk, N,N'-di-6-undecanyl-PDI (70 mg, 0.10 mmol), B_2pin_2 (200 mg, 0.80 mmol), $[Ir(OMe)(cod)]_2$ (3.3 mg, 0.005 mmol) and ligand (0.01 mmol) were dissolved in dry dioxane (4 mL). The orange solution was stirred in a plate under reflux (120 ºC) for 72h. After that time, the reaction was cooled to rt, the solvent was evaporated under vacuum and the resulting crude oil was purified by SiO_2 column chromatography ($CH_2Cl_2 \rightarrow CH_2Cl_2$/MeOH, 25:1). The fraction containing the product was triturated in MeOH, filtered and washed with MeOH (5 mL) to obtain the products as dark purple solids.

L1 (1.6 mg). Product obtained: **2b** (27 mg, 33%) and mixture di- + tri- borylated.

L2 (1.9 mg). Product obtained: **2b** (25 mg, 30%) and mixture di- + tri- borylated.

L3 (2.7 mg). Product obtained: **2b** (43 mg, 52%) and starting material (**2'**).

L4 (2.6 mg). Product obtained: **2a** (30 mg, 25%) + mixture of tri-, di-, mono- and unreacted.

L5 (5.3 mg). Product obtained: **2a** (100 mg, 83%).

L6 (3.5 mg). Product obtained: **2a** (22 mg, 18%) + mixture of tri-, di-, mono- and unreacted.

Method 2: In a 5 mL microwave vial, N,N'-di-6-undecanyl-PDI (70 mg, 0.10 mmol), B_2pin_2 (200 mg, 0.80 mmol), $[Ir(OMe)(cod)]_2$ (3.3 mg, 0.005 mmol) and $P(C_6F_5)_3$ (5.3 mg, 0.01 mmol) were dissolved in dry THF (4 mL). The orange solution was stirred in a plate at reflux (150 ºC) for 1h, cooled and evaporated under vacuum. The crude oil was purified by SiO_2 column chromatography (CH_2Cl_2→CH_2Cl_2/MeOH, 25:1). The fraction containing the product was triturated in MeOH, filtered and washed with MeOH (5 mL) to obtain **2a** (89 mg, 74%) as a dark purple solid.

Product **2a**: **^{1}H NMR** (300 MHz, $CDCl_3$): 8.51 (s, 4H); 5.10 – 4.98 (m, 2H); 2.17 (dd, $^3J_{H-H}$ = 12.4, $^4J_{H-H}$ = 5.4 Hz, 4H); 1.82 (dd, $^3J_{H-H}$ = 12.4, $^4J_{H-H}$ = 5.4 Hz, 4H); 1.54 (s, 48H); 1.28 – 1.22 (m, 24H); 0.85 (t, $^3J_{H-H}$ = 7.0, 12H). **^{13}C{1H} NMR** (75 MHz, $CDCl_3$): 165.9 (C), 138.5 (C), 133.3 (C), 128.2 (C), 126.9 (C), 125.9 (CH), 84.6 (C), 54.9 (CH), 32.4 (CH_2), 31.8 (CH_2), 26.7 (CH_2), 25.2 (CH_3), 22.6 (CH_2), 14.2 (CH_3). **IR** (ATR, cm^{-1}): 2956 (s), 2927 (s), 2960 (m), 1688 (s), 1651 (s), 1606 (w), 1548 (m), 1435 (m), 1380 (s), 1336 (s), 1314 (m), 1231 (m), 1214 (s), 1165 (s), 962 (s), 822 (s), 755 (s).

Product **2b**: **^{1}H NMR** (300 MHz, $CDCl_3$): 8.76 – 8.40 (m, 7H), 5.29 – 5.05 (m, 2H), 2.34 – 2.08 (m, 4H), 1.89 – 1.83 (m, 4H), 1.56 (s, 12H), 1.43 (s, 4H), 1.32 – 1.08 (m, 20H), 0.99 – 0.64 (m, 12H). **^{13}C{1H} NMR** (75 MHz, $CDCl_3$): 163.8 (C), 134.7, 133.3, 131.3 (CH), 129.7, 128.9, 126.6, 123.2 (CH), 84.8 (C), 54.9 (CH), 32.5 (CH_2), 31.9 (CH_2), 26.8 (CH_2), 25.2 (CH_3), 22.7 (CH_2), 14.2 (CH_3). **IR** (ATR, cm^{-1}): 2954 (s), 2925 (s), 2857 (m), 1694 (s), 1652 (s), 1593 (w), 1571 (m), 1435 (m), 1337 (s), 1311 (m), 1208 (m), 1105 (m), 967 (s), 851 (m), 810 (m), 747 (m).

S.1.2.3. Synthesis of *ortho*-borylated PMIs (3a, 3b and 3c)

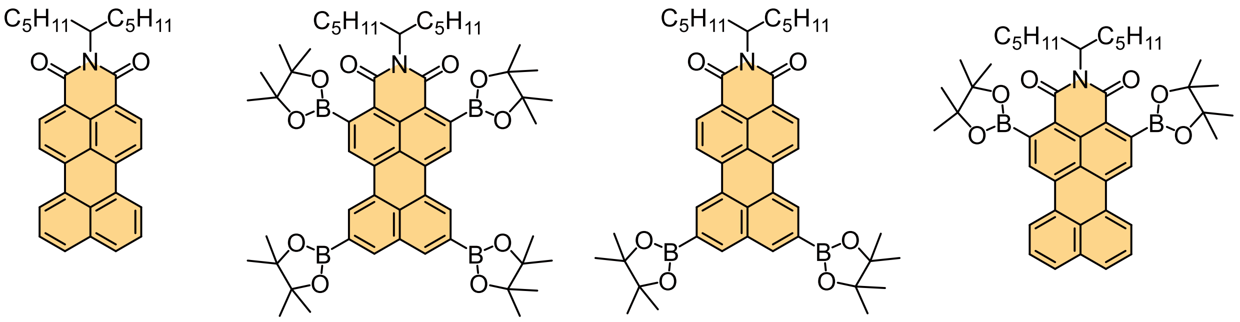

3' 3a 3b 3c

Figure S4 Structures of the PMI derivatives from this study. PMI (**3'**), its tetra- *ortho*-borylated derivative (**3a**), di-borylated opposite to the imide (**3b**) and di-borylated in *ortho* to the imide (**3c**).

Compound 3b. Method 1: N-6-undecanyl-PMI (50 mg, 0.1 mmol), B_2pin_2 (213 mg, 0.8 mmol), $[Ir(OMe)(cod)]_2$ (3.5 mg, 0.005 mmol), and dtbpy (11.3 mg, 0.04 mmol) were put in a Schlenk under Ar atmosphere and dissolved in dry dioxane. The Schlenk was heated in a plate at 120 ºC for 90 hours. The reaction mixture was cooled to rt and the solvent evaporated under vacuum, resulting in a crude oil that was purified by SiO_2 column chromatography (Heptane/CH_2Cl_2 1:1 to CH_2Cl_2). After concentration under reduced pressure product **3b** was obtained as a red solid (42 mg, 55%).

Method 2: N-6-undecanyl-PMI (50 mg, 0.1 mmol), B_2pin_2 (213 mg, 0.8 mmol), $[Ir(OMe)(cod)]_2$ (3.5 mg, 0.005 mmol), and dtbpy (11.3 mg, 0.04 mmol) were dissolved in dry THF (5 mL) in a microwave vial under Ar atmosphere. The vial was heated under Mw at 110 ºC for 1 hour. The reaction mixture was cooled to rt and the solvent evaporated under vacuum, resulting in a crude oil that was purified by SiO_2 column chromatography (Heptane/CH_2Cl_2 1:1 to CH_2Cl_2). After concentration under reduced pressure product **3b** was obtained as a red solid (67 mg, 88%).

^{1}H NMR (500 MHz, $CDCl_3$): 8.81 (s, 2H); 8.67-8.62 (m, 4H); 8.42 (s, 2H); 5.21 (m, 1H); 2.31-2.20 (m, 2H); 1.88-1.82 (m, 2H); 1.44 (s, 24H); 1.30-1.21 (m, 12H); 0.86-0.81 (m, 6H). **^{13}C{1H} NMR** (126 MHz, $CDCl_3$): 165.4 (CO); 164.4 (CO); 139.5 (CH); 137.2 (C); 133.1 (C); 132.0 (C); 131.2 (C); 130.8 (C); 130.1 (C); 129.5 (CH); 128.4 (C); 127.6 (C); 126.9 (C); 120.5 (CH); 84.5 (CH); 54.5 (CH_3); 32.6 (CH_2); 32.0 (CH_2); 26.8 (CH_2); 25.1 (CH_3); 22.7 (CH_2); 14.2 (CH_3). **IR** (ATR, cm^{-1}): 2976 (m), 2953 (m), 2926 (s), 2858 (m), 1689 (s), 1651 (s), 1584 (s), 1415 (m), 1371 (m), 1347 (s), 1296 (s), 1286 (m), 1207 (s), 965 (m), 848 (m), 752 (m), 686 (m). **HRMS** (APCI) m/z: = $[M+H]^+$ Calcd. for 728.4303; Found 728.4283.

Compound 3c. Method 1: In a 50 mL Schlenk, N-6-undecanyl-PMI (50 mg, 0.1 mmol), B_2pin_2 (225 mg, 0.8 mmol), $[Ir(OMe)(cod)]_2$ (7 mg, 0.01 mmol) and $P(C_6F_5)_3$ (22 mg, 0.04 mmol) were put under Ar atmosphere and dissolved in dry dioxane flask (5 mL). The orange mixture was stirred under reflux (120 ºC) in a plate with a condenser for 72 hours. Once the reaction is finished, it was cooled to rt and the solvent was evaporated under vacuum and the resulting oil was purified by SiO_2 column chromatographic column (CH_2Cl_2 → CH_2Cl_2/MeOH 20:1). The concentrated fraction containing the product was redissolved in CH_2Cl_2 (10 mL) and precipitated with a mixture of MeOH/EtOH (4:1 mL) by evaporating the CH_2Cl_2 at reduced pressure and filtering *in vacuo*. Product **3c** was obtained, as a red powder (58 mg, 76%).

Method 2: N-6-undecanyl-PMI (50 mg, 0.1 mmol), B_2pin_2 (213 mg, 0.8 mmol), $[Ir(OMe)(cod)]_2$ (3.5 mg, 0.005 mmol), and $P(C_6F_5)_3$ (35 mg, 0.06 mmol) were dissolved in dry THF (5 mL) in a microwave vial under Ar atmosphere. The vial was heated under Mw at 110 ºC for 1 hour. The reaction mixture was cooled to rt and the solvent evaporated under vacuum and the resulting purified by SiO_2 column chromatographic column (CH_2Cl_2 → CH_2Cl_2/MeOH 20:1). The fraction containing the product was redissolved in CH_2Cl_2 (10 mL) and precipitated with a mixture of MeOH/EtOH (4:1 mL) by evaporating the CH_2Cl_2 at reduced pressure and filtering *in vacuo*. Product **3c** was obtained, as a red powder (56 mg, 74%).

^{1}H NMR (300 MHz, $CDCl_3$): 8.46 (d, $^4J_{H-H}$ = 7.7 Hz, 2H); 8.35 (s, 2H); 7.85 (d, $^4J_{H-H}$ = 7.8 Hz, 2H); 7.60 (t, $^3J_{H-H}$ = 7.8 Hz, 2H); 5.05 (m, 1H); 2.22-2.17 (m, 2H); 1.84-1.77 (m, 2H); 1.54 (s, 24H); 1.28 (m, 12H); 0.86 (t, $^3J_{H-H}$ = 6.4 Hz, 6H). **^{13}C{1H} NMR** (126 MHz, $CDCl_3$): 166.1 (CO); 135.5 (C); 134.6 (C); 130.7 (CH); 129.6 (C); 128.5 (C); 128.5 (C); 127.1 (C); 127.0 (CH); 123.6 (CH); 123.2 (CH); 84.4 (C); 54.6 (CH_3); 32.4 (CH_2); 31.9 (CH_2); 26.7 (CH_2); 25.2 (CH_3); 22.7 (CH_2); 14.2 (CH_3). **IR** (ATR, cm^{-1}): 2972 (m), 2955 (m), 2928 (s), 2859 (m), 1678 (s), 1644 (s), 1547 (s), 1467 (w), 1366 (s), 1352 (s), 1305 (s), 1290 (m), 1210 (s), 1075 (s), 964 (m), 839 (m), 755 (m), 618 (s). **HRMS** (APCI) m/z: $[M+H]^+$ Calcd for 728.4303; Found 728.4294.

Compound 3a. Method 1: In a 50 mL Schlenk, N-6-undecanyl-PMI (50 mg, 0.1 mmol), B_2pin_2 (225 mg, 0.8 mmol), $[Ir(OMe)(cod)]_2$ (7 mg, 0.01 mmol) and $P(C_6F_5)_3$ (22 mg, 0.04 mmol) were put under Ar atmosphere and dissolved in dry dioxane (5 mL). The orange mixture was stirred under reflux (120 ºC) in a plate with a condenser for 72 hours. Within the same Schlenk $[Ir(OMe)(cod)]_2$ (3.5 mg, 0.005 mmol), and dtbpy (11.3 mg, 0.04 mmol) dissolved in 2 mL of dry dioxane were added and the reaction mixture was further heated in a plate at 120 ºC for another 72 hours. The mixture

was cooled to rt and the solvent was evaporated under vacuum. The resulting oil was purified by SiO_2 column chromatographic column (CH_2Cl_2 → CH_2Cl_2/MeOH 20:1). The concentrated fraction containing the product was redissolved in CH_2Cl_2 (10 mL) and precipitated with a mixture of MeOH/EtOH (4:1 mL) by evaporating the CH_2Cl_2 at reduced pressure and filtering. Product **3a** was obtained, as a red powder (64 mg, 62%).

Method 2: In a 5 mL microwave vial, N-6-undecanyl-PMI (50 mg, 0.1 mmol), B_2pin_2 (225 mg, 0.8 mmol), $[Ir(OMe)(cod)]_2$ (7 mg, 0.01 mmol) and $P(PC_6F_5)_3$ (22 mg, 0.04 mmol) were put under Ar atmosphere and dissolved in dry THF flask (4 mL). The orange mixture was in the Mw heated at 150 ºC for 1h. Within the same vial $[Ir(OMe)(cod)]_2$ (3.5 mg, 0.005 mmol), and dtbpy (11.3 mg, 0.04 mmol) dissolved in 1 mL of dry THF were added and the reaction mixture was further heated at 150 ºC for another hour. The mixture was cooled to rt and the solvent was evaporated under vacuum. The resulting oil was purified by SiO_2 column chromatographic column (CH_2Cl_2 → CH_2Cl_2/MeOH 20:1). The concentrated fraction containing the product was redissolved in CH_2Cl_2 (10 mL) and precipitated with a mixture of MeOH/EtOH (4:1 mL) by evaporating the CH_2Cl_2 at reduced pressure and filtering. Product **3a** was obtained, as a red powder (67 mg, 65%).

^{1}H NMR (500 MHz, $CDCl_3$): 8.80 (s, 2H), 8.45 (s, 2H), 8.42 (s, 2H), 5.05 (s, 1H), 2.21 – 2.15 (m, 2H), 1.85 – 1.80 (m, 2H), 1.56 (s, 24H), 1.44 (s, 24H), 1.31 – 1.24 (m, 12H), 0.85 (t, $^3J_{H\text{-}H}$ = 7.0 Hz, 6H). **^{13}C{1H} NMR** (126 MHz, $CDCl_3$): 166.3 (CO), 139.5 (CH), 135.7 (C), 133.2 (C), 131.3 (C), 129.4 (CH), 128.7 (C), 127.2 (C), 123.3 (CH), 84.5 (C), 54.5 (CH), 32.4 (CH_2), 31.9 (CH_2), 26.7(CH_2), 25.4 (CH_3), 25.1 (CH_3), 22.7 (CH_2), 14.3 (CH_3). **IR** (ATR, cm^{-1}): 2954 (m), 2928 (s), 2859 (m), 1683 (s), 1647 (s), 1607 (s), 1547 (s), 1463 (w), 1402 (br), 1343 (s), 1298 (s), 1270 (s), 1270 (s), 1138 (s), 1112 (s), 1007 (m), 876 (m), 828 (w), 767 (w), 689 (w). **HRMS** (APCI) m/z: $[M+H]^+$ Calcd. for 980.6025; Found 980.6024.

S.1.3. ^{1}H NMR Spectra Comparison and Temperature Dependence

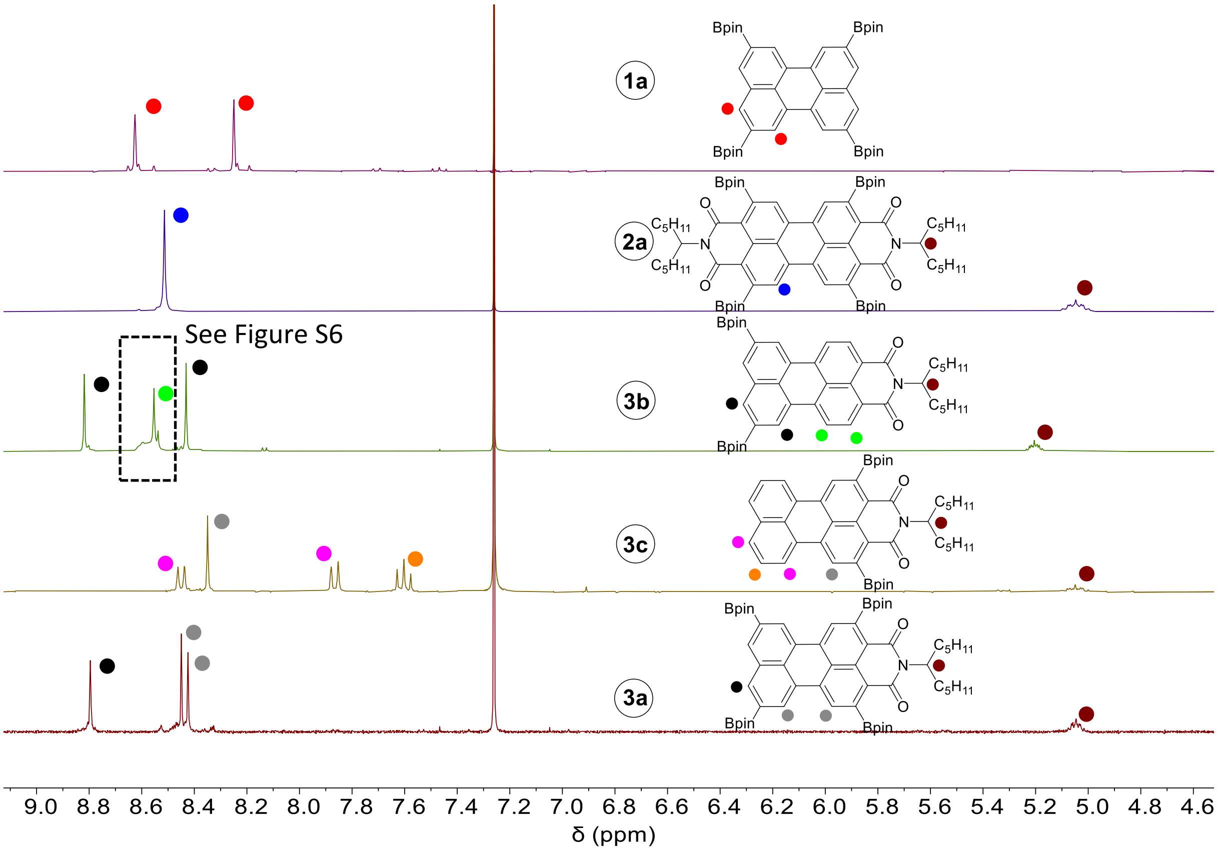

Figure S5 Zoomed ^{1}H-NMR spectra (4.6 – 9.1 ppm) for comparison of compound **1a** (pink), compound **2a** (purple), compound **3b** (green), compound **3c** (orange), and compound **3c** (red) in $CDCl_3$ at room temperature.

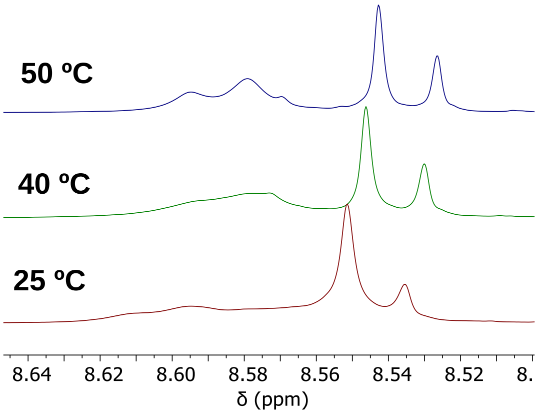

Figure S6 Zoomed ^{1}H-NMR spectra (8.50 – 8.65 ppm) for comparison of compound **3b** in $CDCl_3$ at 25 ºC (red), 40 ºC (green) and 50 ºC (blue).

S.2. COMPUTATIONAL STUDIES

The structures of Perylene **1’**, Perylene tetraborylated **1a**, PDI **2’**, PDI tetraborylated **2a**, PMI **3’**, PMI tetraborylated **3a** and the diborylated in ortho to the imide **3c** or in the opposite ortho positions **3b** were optimized at DFT level using the B3LYP[S3] functional in the 6-31G(d,p) level with chloroform as solvent. Analytical harmonic frequencies were computed at the same level of theory. All of the calculations were carried out by the methods implemented in Gaussian 16 package.[S4] The imide substituent for the calculations was changed from 6-undecanyl imide to isopropyl imide in order to reduce the time and simplify the calculations.

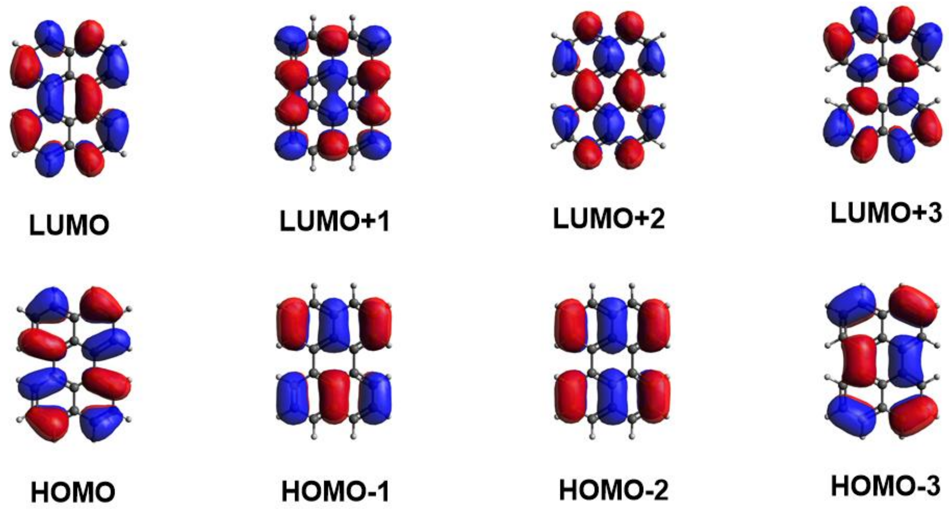

Figure S7 Kohn–Sham orbital representations of the H-3 to L+3 levels of **1‘**.

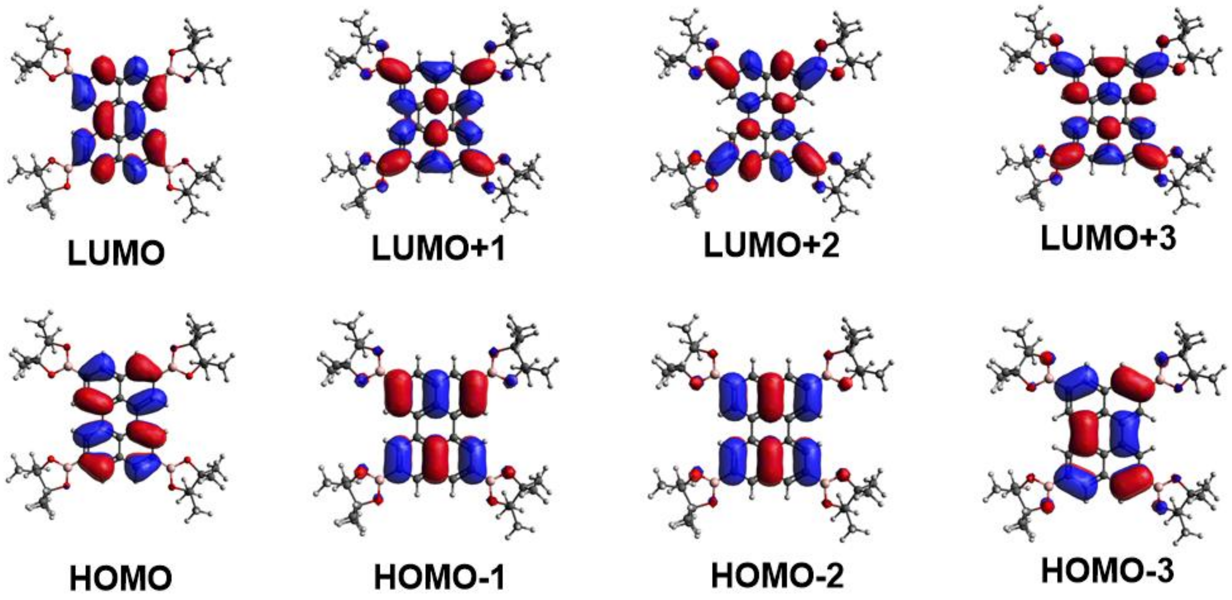

Figure S8 Kohn–Sham orbital representations of the H-3 to L+3 levels of **1a**.

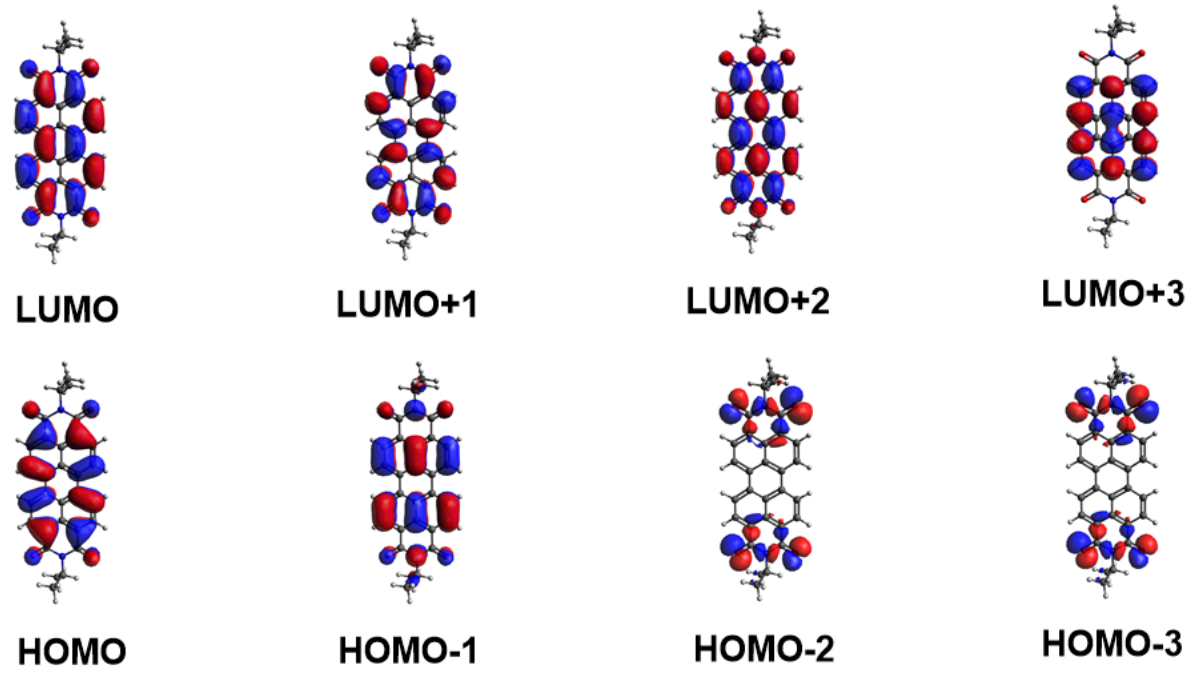

Figure S9 Kohn–Sham orbital representations of the H-3 to L+3 levels of **2’**.

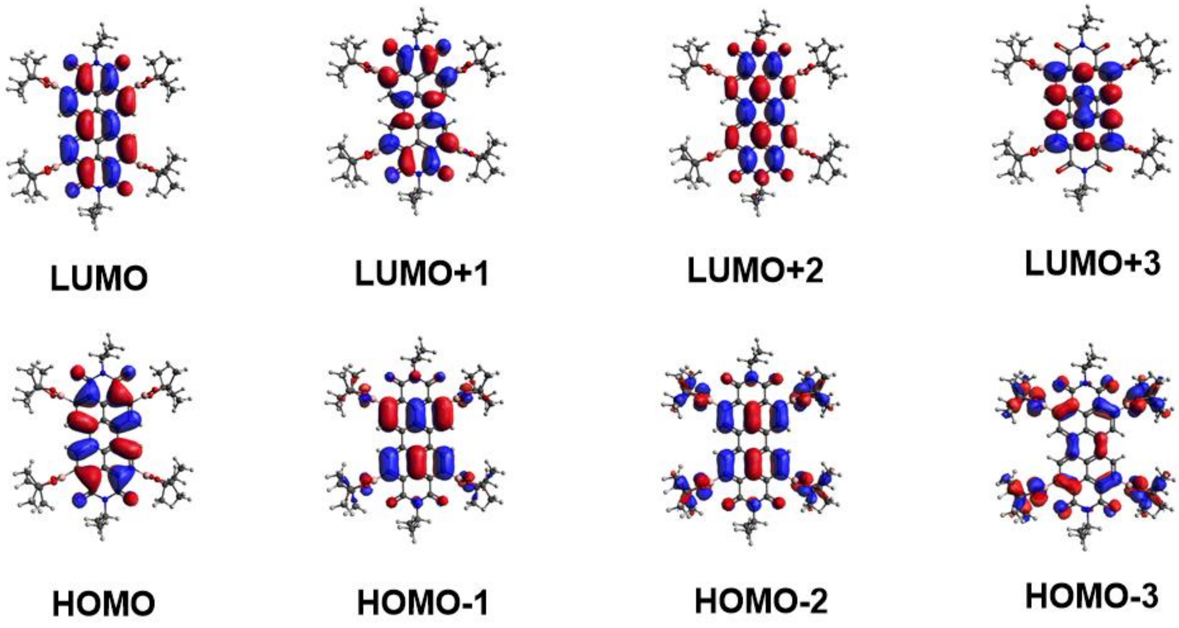

Figure S10 Kohn–Sham orbital representations of the H-3 to L+3 levels of **2a**.

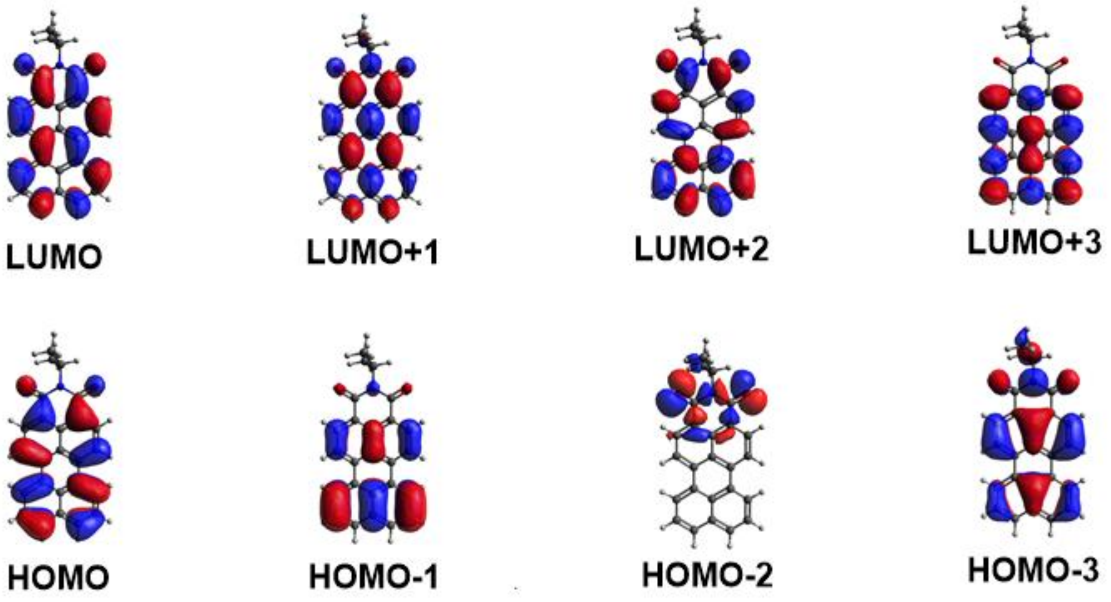

Figure S11 Kohn–Sham orbital representations of the H-3 to L+3 levels of **3'**.

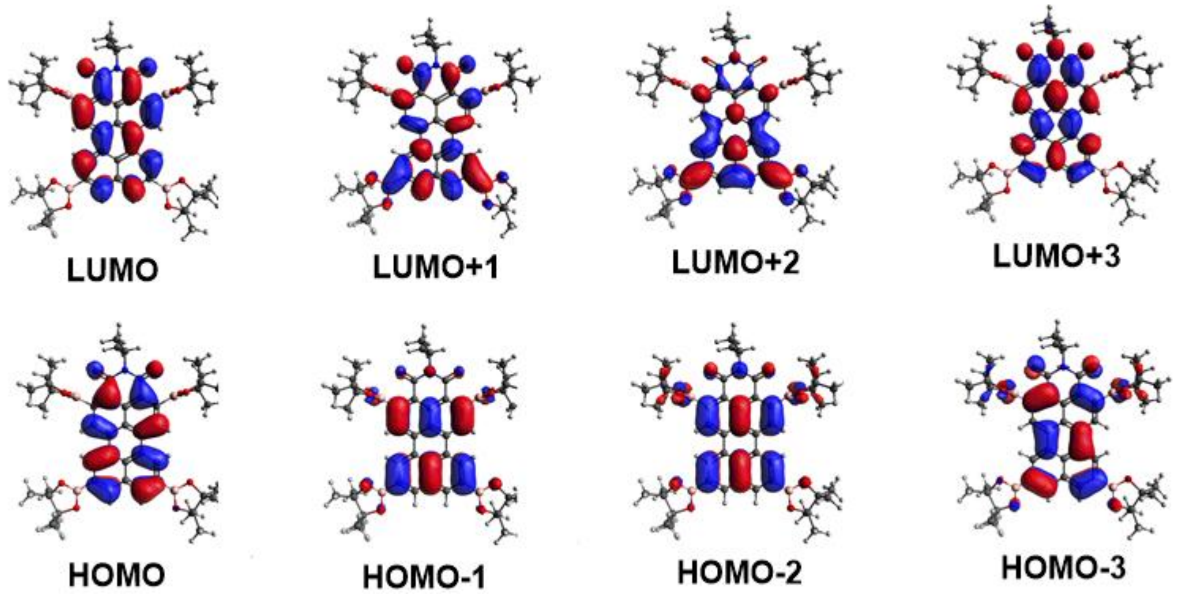

Figure S12 Kohn–Sham orbital representations of the H-3 to L+3 levels of **3a**.

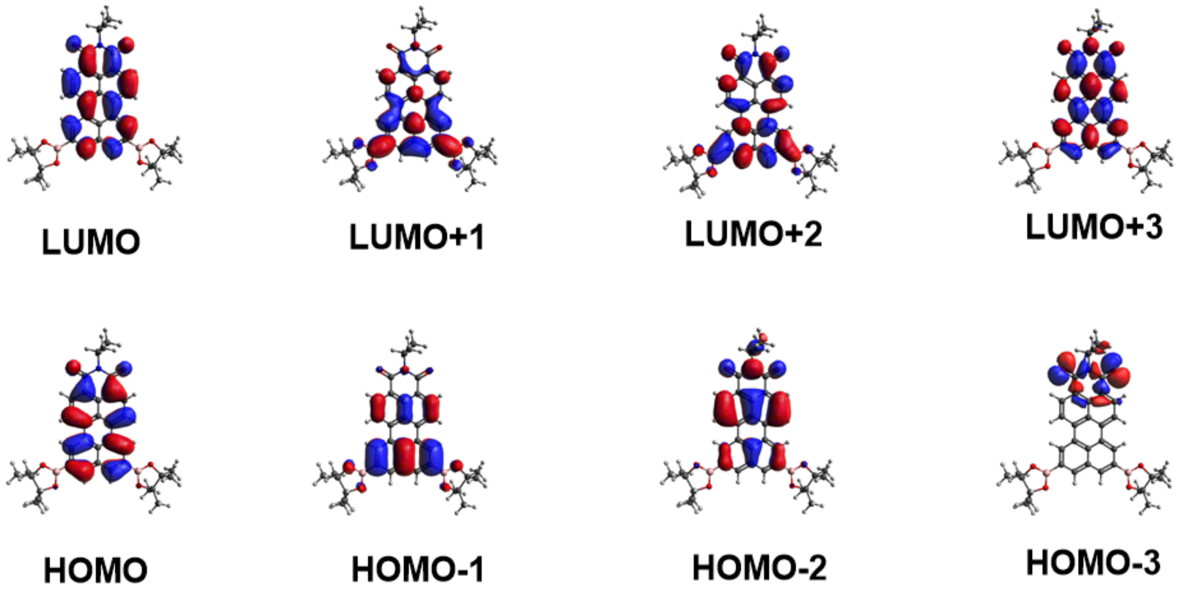

Figure S13 Kohn–Sham orbital representations of the H-3 to L+3 levels of **3b**.

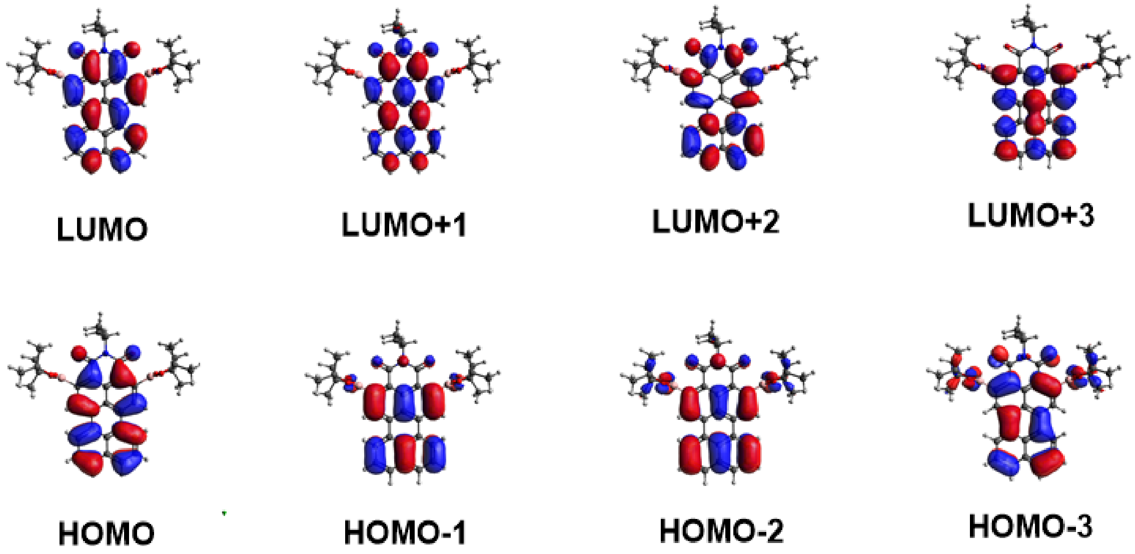

Figure S14 Kohn–Sham orbital representations of the H-3 to L+3 levels of **3c**.

The resulting electrostatic maps (ESPs) showed how the electron density is distributed, ESPs are represented from -0.05 to 0.05 au, from red to blue, respectively.

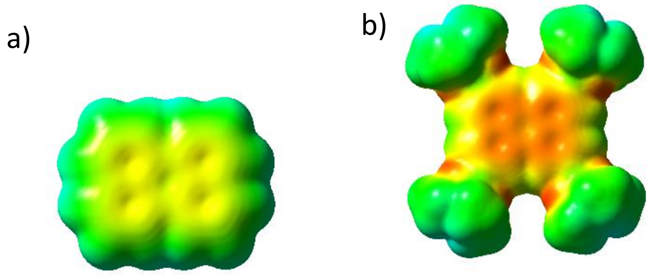

Figure S15 Electrostatic potential surfaces (ESP) calculated at B3LYP/6-31+G(d,p) level of theory of (a) perylene **1'** and (b) perylene tetraboronate **1a**.

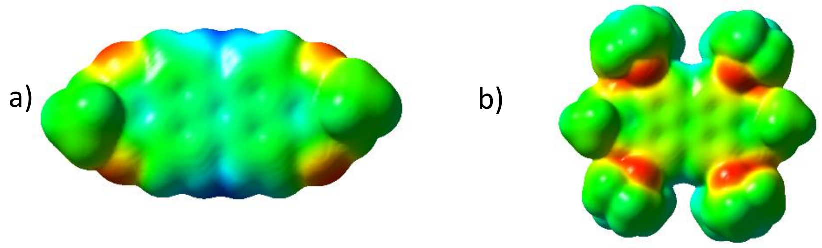

Figure S16 Electrostatic potential surfaces (ESP) calculated at B3LYP/6-31+G(d,p) level of theory of (a) PDI **2'** and (b) PDI tetraboronate **2a**.

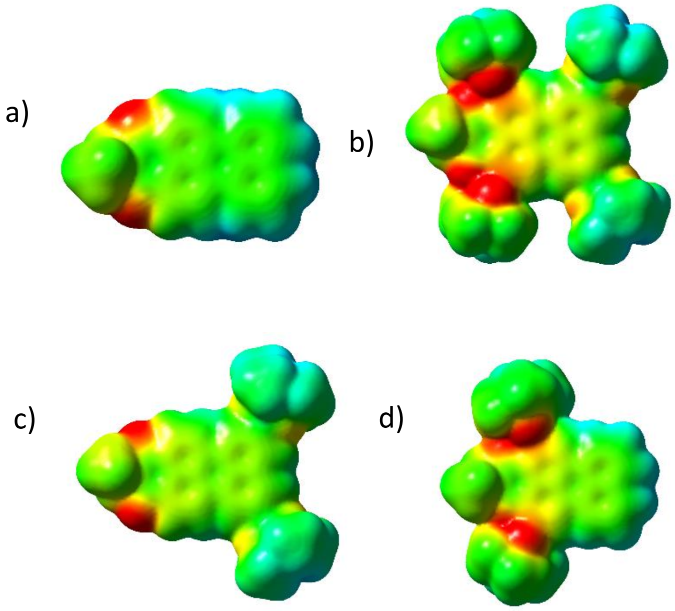

Figure S17 Electrostatic potential surfaces (ESP) calculated at B3LYP/6-31+G(d,p) level of theory of (a) PMI **3‘**, (b) PMI **3a**, (c) PMI **3b** and (d) PMI **3c**.

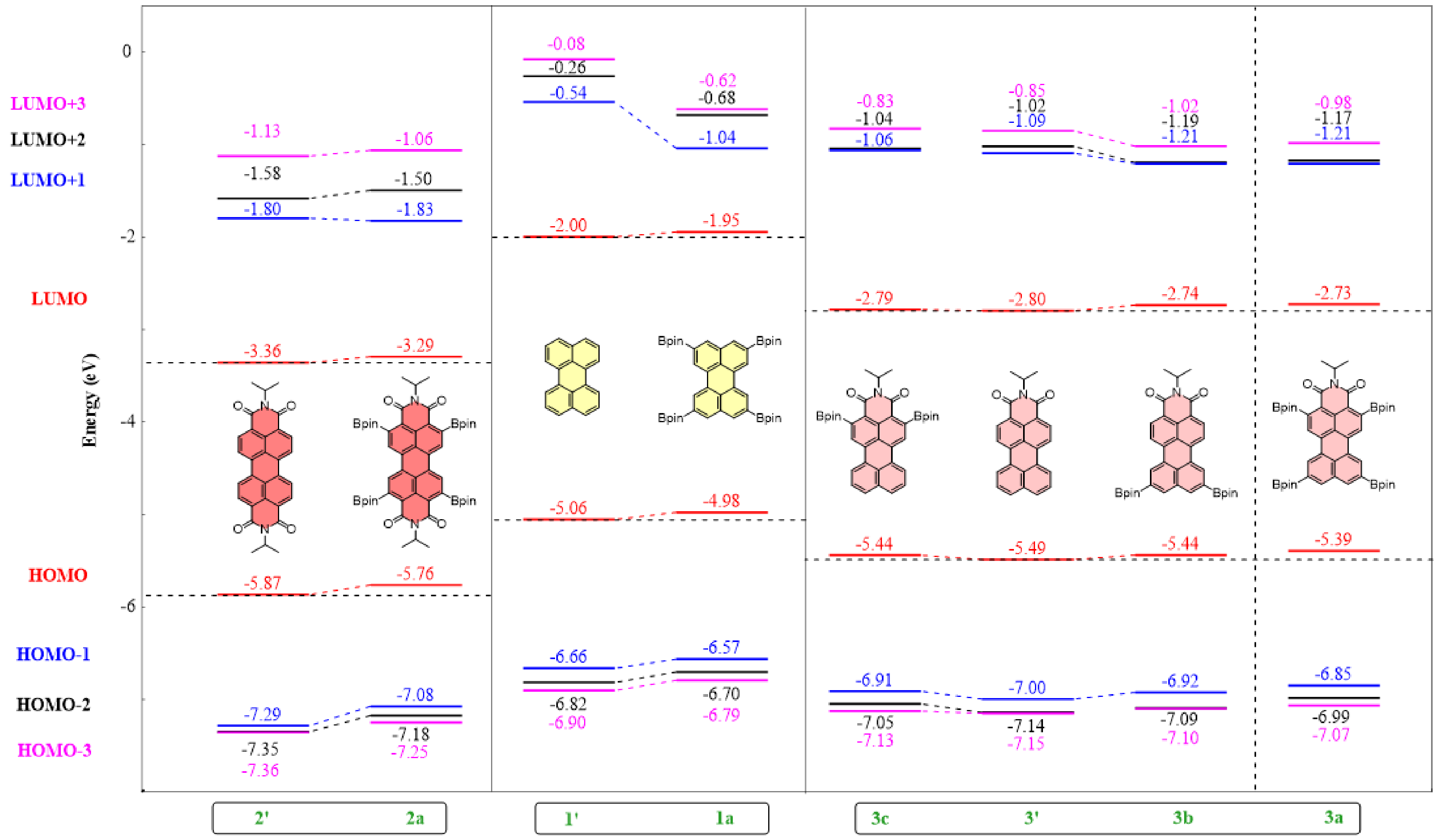

Figure S18 HOMO-LUMO levels and energy calculated for compounds **1'-1a**, **2'-2a** and **3'-3a-3b-3c**.

S.2.1. Cartesian Coordinates

Table S1. Cartesian coordinates for the optimized structure of **1’** in chloroform using B3LYP/6-31G(d,p) level.

Energy = -769.429 a.u.

C	-2.88588925	-2.42311301	-0.04510120
C	-1.47894663	-2.42846325	-0.03812840
C	-0.73838152	-1.25031575	-0.01025577
C	-1.43944176	-0.00000092	-0.00009712
C	-2.87503331	0.00000401	-0.00014642
C	-3.57606988	-1.23304797	-0.02305824
C	-0.73837924	1.25030952	0.01028846
C	-1.47893397	2.42845456	0.03855713
C	-2.88587822	2.42311368	0.04530816
C	-3.57606345	1.23305967	0.02282414
C	0.73838169	-1.25031557	0.01026425
C	1.43944172	-0.00000091	0.00009640
C	0.73837907	1.25030966	-0.01028019
C	1.47894704	-2.42846295	0.03814152
C	2.88588962	-2.42311265	0.04510180
C	3.57607007	-1.23304773	0.02304291
C	2.87503332	0.00000411	0.00013350
C	3.57606323	1.23305985	-0.02283924
C	2.88587786	2.42311402	-0.04530741
C	1.47893359	2.42845485	-0.03854417

H	-0.97671659	-3.38830328	-0.05691406
H	-4.66228255	-1.21902070	-0.02466916
H	-0.97668881	3.38827594	0.05789732
H	-4.66227649	1.21904010	0.02425068
H	0.97671705	-3.38830276	0.05694019
H	4.66228277	-1.21902057	0.02463946
H	4.66227627	1.21904016	-0.02428001
H	0.97668839	3.38827645	-0.05787215
H	-3.42067439	3.36784946	0.06701975
H	3.42067375	3.36785002	-0.06701583
H	3.42068873	-3.36785181	0.06659072
H	-3.42068808	-3.36785239	-0.06658760

Table S2. Cartesian coordinates for the optimized structure of **1a** in chloroform using B3LYP/6-31G(d,p) level.

Energy = -2412.269 a.u.

C	-2.44348369	2.90032671	-0.00808901
C	-2.42595539	1.48270591	-0.00404393
C	-1.25174784	0.73924502	-0.00138301
C	-0.00000402	1.43709805	-0.00000041
C	-0.00000391	2.87171438	0.00000283
C	-1.23200206	3.57129500	-0.00595564
C	1.25173990	0.73924521	0.00137698
C	2.42594737	1.48270594	0.00403328

C	2.44347586	2.90032695	0.00808738
C	1.23199440	3.57129510	0.00596397
C	-1.25174769	-0.73921656	0.00138181
C	-0.00000365	-1.43706916	-0.00000557
C	1.25174013	-0.73921614	-0.00138966
C	-2.42595523	-1.48267786	0.00404788
C	-2.44348310	-2.90029826	0.00808944
C	-1.23200099	-3.57126627	0.00595248
C	-0.00000324	-2.87168544	-0.00000706
C	1.23199495	-3.57126576	-0.00596672
C	2.44347665	-2.90029721	-0.00809726
C	2.42594798	-1.48267673	-0.00405156
B	-3.79161914	3.67776345	-0.01677729
B	3.79161459	3.67775815	0.01677313
B	-3.79161211	-3.67774602	0.01677403
B	3.79160778	-3.67774135	-0.01677595
O	-3.87685751	5.04726681	0.04611177
C	-5.26706596	5.41181042	-0.21533612
C	-6.03588476	4.08063893	0.14684894
O	-5.01670277	3.06115438	-0.08993363
O	3.87686311	5.04726235	-0.04607644
C	5.26708007	5.41178740	0.21535828
C	6.03588196	4.08062052	-0.14687171
O	5.01669467	3.06113732	0.08989443

O	5.01669627	-3.06114896	-0.08998287
C	6.03587174	-4.08063477	0.14682259
C	5.26703127	-5.41181328	-0.21529615
O	3.87683187	-5.04724386	0.04615910
O	-5.01670276	-3.06115953	0.08999100
C	-6.03587540	-4.08064924	-0.14681084
C	-5.26702590	-5.41182587	0.21529613
O	-3.87683041	-5.04724875	-0.04616762
C	-5.61864157	6.62060139	0.64800610
C	-5.35178950	5.78033263	-1.70173427
C	-6.41920864	3.98075810	1.62848904
C	-7.24395245	3.76756621	-0.73236274
C	6.41919070	3.98078272	-1.62851881
C	7.24395594	3.76751637	0.73231980
C	5.61865337	6.62059514	-0.64796069
C	5.35183622	5.78026961	1.70176444
C	5.35172663	-5.78039682	-1.70168070
C	5.61860601	-6.62057487	0.64808815
C	7.24392615	-3.76760957	-0.73242414
C	6.41922235	-3.98069442	1.62845169
C	-7.24392445	-3.76763331	0.73244663
C	-6.41923796	-3.98070414	-1.62843649
C	-5.35170827	-5.78041698	1.70167962
C	-5.61860120	-6.62058519	-0.64809120

H	-3.38474112	0.97728453	-0.00278111
H	-1.21102337	4.65795554	-0.01000805
H	3.38473315	0.97728472	0.00275961
H	1.21101502	4.65795565	0.01002474
H	-3.38474085	-0.97725604	0.00279068
H	-1.21102282	-4.65792670	0.01000228
H	1.21101702	-4.65792621	-0.01001984
H	3.38473338	-0.97725454	-0.00278678
H	-5.01148987	7.47764469	0.34294325
H	-5.42989516	6.43115223	1.70596621
H	-6.67158095	6.89245932	0.52369288
H	-4.64258599	6.58634573	-1.90866193
H	-5.09404973	4.93032211	-2.33958408
H	-6.35316468	6.12671619	-1.97248758
H	-5.56516620	4.19372966	2.27733675
H	-6.75907982	2.96315407	1.83901958
H	-7.22828917	4.67191143	1.88105305
H	-6.96896904	3.68988802	-1.78551406
H	-7.68318343	2.81422547	-0.42512800
H	-8.01093063	4.54142925	-0.62722872
H	5.56515170	4.19381703	-2.27735055
H	6.75901749	2.96317287	-1.83909305
H	7.22829811	4.67191233	-1.88106178
H	6.96898131	3.68981571	1.78547197

H	7.68317650	2.81417955	0.42505812
H	8.01093872	4.54137639	0.62719701
H	5.01151150	7.47763612	-0.34287195
H	5.42989310	6.43116958	-1.70592270
H	6.67159645	6.89244260	-0.52365419
H	4.64262059	6.58626091	1.90873523
H	5.09413375	4.93023420	2.33959634
H	6.35321130	6.12666718	1.97250003
H	5.09398492	-4.93040960	-2.33956081
H	4.64251104	-6.58641072	-1.90856378
H	6.35309350	-6.12680223	-1.97243704
H	5.42987383	-6.43108449	1.70604328
H	5.01144244	-7.47762384	0.34306494
H	6.67154121	-6.89244784	0.52377298
H	7.68317147	-2.81426031	-0.42523625
H	6.96892432	-3.68997155	-1.78557365
H	8.01089946	-4.54147506	-0.62727272
H	6.75912867	-2.96309084	1.83892752
H	5.56518409	-4.19360739	2.27732405
H	7.22828563	-4.67186055	1.88103589
H	-7.68317687	-2.81428505	0.42526593
H	-6.96891469	-3.68999801	1.78559427
H	-8.01089486	-4.54150211	0.62729839
H	-6.75915158	-2.96310152	-1.83890502

H	-5.56520356	-4.19360933	-2.27731646
H	-7.22829933	-4.67187363	-1.88101762
H	-5.09396312	-4.93043247	2.33956189
H	-4.64248920	-6.58643040	1.90855284
H	-6.35307212	-6.12682610	1.97244250
H	-5.42987786	-6.43108910	-1.70604689
H	-5.01143096	-7.47763246	-0.34307649
H	-6.67153406	-6.89246408	-0.52376939

Table S3. Cartesian coordinates for the optimized structure of **2'** in chloroform using B3LYP/6-31G(d,p) level.

Energy = -1567.046 a.u.

0 1

C	-2.80353559	2.50716176	-0.03406884
C	-1.40533386	2.47533809	-0.02829436
C	-0.69599768	1.27248458	-0.00701853
C	-1.42983585	0.04429583	0.00029751
C	-2.85781364	0.08832015	-0.00018436
C	-3.53414221	1.33025841	-0.01767980
C	-0.77326591	-1.22691568	0.00817811
C	-1.55555206	-2.38348632	0.02977952
C	-2.95315043	-2.32899799	0.03466337
C	-3.60957722	-1.10950296	0.01706866
C	0.77328511	1.22687157	0.00835306

C	1.42985531	-0.04433916	0.00030043
C	0.69601686	-1.27252669	-0.00718105
C	1.55557174	2.38343915	0.03011623
C	2.95317079	2.32895193	0.03490333
C	3.60959744	1.10945915	0.01713017
C	2.85783338	-0.08836354	-0.00017167
C	3.53416069	-1.33030322	-0.01770185
C	2.80355320	-2.50720387	-0.03425602
C	1.40535183	-2.47537869	-0.02858409
C	-5.09231707	-1.08097419	0.01858058
N	-5.71625477	0.17341158	-0.00262033
C	-5.01435878	1.39324277	-0.02117766
O	-5.60733480	2.46798041	-0.03909771
O	-5.75058393	-2.11728654	0.03713615
C	-7.21180355	0.24820357	-0.00563698
C	-7.80833829	-0.36369760	-1.27795647
C	-7.81142969	-0.31787167	1.28629119
C	5.09233778	1.08092455	0.01840166
N	5.71626832	-0.17345578	-0.00263858
C	5.01437646	-1.39330115	-0.02092506
O	5.60732035	-2.46806412	-0.03848184
O	5.75065061	2.11721488	0.03657534
C	7.21181525	-0.24812637	-0.00565282
C	7.80823341	0.36385233	-1.27798019

C	7.81130841	0.31818036	1.28623126
H	-0.87866790	3.42118565	-0.04297569
H	-1.08795336	-3.35978626	0.04528960
H	1.08797215	3.35973588	0.04579687
H	0.87868573	-3.42122386	-0.04342357
H	-7.40539051	1.31971934	-0.02500082
H	-8.88280820	-0.15589540	-1.30195780
H	-7.36023539	0.08391954	-2.17042582
H	-7.66217578	-1.44386097	-1.31327996
H	-8.88571449	-0.10817920	1.30089833
H	-7.36453326	0.16045783	2.16329923
H	-7.66648715	-1.39627024	1.36001521
H	7.40556493	-1.31961167	-0.02494701
H	8.88273713	0.15622783	-1.30197113
H	7.36020745	-0.08385980	-2.17044043
H	7.66187574	1.44399236	-1.31333691
H	8.88565575	0.10881126	1.30082455
H	7.36456671	-0.16023664	2.16326826
H	7.66605401	1.39654578	1.35990103
H	-3.54016836	-3.24004026	0.05116239
H	3.33288707	-3.45291785	-0.05076769
H	3.54018931	3.23999269	0.05144754
H	-3.33286952	3.45287565	-0.05057117

Table S4. Cartesian coordinates for the optimized structure of **2a** in chloroform using B3LYP/6-31G(d,p) level.

Energy = -3209.867 a.u.

C	-2.61393753	2.74291704	0.01520974
C	-1.21564165	2.57495468	0.01330155
C	-0.59599307	1.32400465	0.00371838
C	-1.41663715	0.15376357	0.00044849
C	-2.83289771	0.30731093	0.00273432
C	-3.40857361	1.59877947	0.01091214
C	-0.86614808	-1.16531547	-0.00329212
C	-1.73972855	-2.25407995	-0.01386834
C	-3.14170374	-2.11829986	-0.01729894
C	-3.67222488	-0.83063806	-0.00244940
C	0.86607622	1.16530144	-0.00329109
C	1.41656600	-0.15377660	0.00045668
C	0.59592199	-1.32401777	0.00371008
C	1.73965575	2.25406603	-0.01388734
C	3.14162949	2.11828927	-0.01731091
C	3.67215445	0.83062882	-0.00246122
C	2.83282776	-0.30732215	0.00275466
C	3.40850322	-1.59879079	0.01091881
C	2.61386704	-2.74293033	0.01518463
C	1.21557090	-2.57496839	0.01326842
C	-5.14092347	-0.67839856	0.01596473

N	-5.67474907	0.61243340	0.01706480
C	-4.87462949	1.76438954	0.01757873
O	-5.37840648	2.88889827	0.02513737
O	-5.87353673	-1.66939430	0.03185175
C	-7.16058523	0.80363211	0.02766463
C	-7.81173836	0.24778002	-1.24333661
C	-7.78869084	0.27680664	1.32246641
C	5.14085399	0.67839140	0.01601938
N	5.67467689	-0.61244336	0.01717098
C	4.87455844	-1.76439766	0.01763013
O	5.37833599	-2.88890721	0.02517846
O	5.87347090	1.66938199	0.03191349
C	7.16051273	-0.80363516	0.02789125
C	7.81176032	-0.24780201	-1.24306965
C	7.78851203	-0.27677671	1.32273091
B	-3.22092205	4.20229569	0.02698285
B	-4.04644258	-3.41418000	-0.03876200
B	4.04635532	3.41417861	-0.03879273
B	3.22086690	-4.20230461	0.02693259
O	-3.32762742	4.93599820	1.17938816
C	-3.92917669	6.22049645	0.82268422
C	-3.61222745	6.32598777	-0.72452429
O	-3.43769824	4.92841641	-1.11456394
O	-4.27630263	-4.15867153	1.08862973

C	-5.14369454	-5.27182047	0.70443554
C	-4.89135483	-5.37594533	-0.85433143
O	-4.43934422	-4.02973641	-1.19843033
O	3.43774425	-4.92837697	-1.11462180
C	3.61247035	-6.32593041	-0.72462484
C	3.92926904	-6.22045803	0.82262719
O	3.32758255	-4.93602612	1.17931949
O	4.43930322	4.02967038	-1.19848760
C	4.89139645	5.37586061	-0.85444737
C	5.14361183	5.27183324	0.70435384
O	4.27613571	4.15876052	1.08855287
C	-5.42527603	6.11350739	1.14120402
C	-3.28543596	7.30131330	1.68889048
C	-4.73245897	6.92239075	-1.57397574
C	-2.28476433	7.02886206	-1.03398060
C	-3.74605939	-6.32418296	-1.23027464
C	-6.13035968	-5.69221970	-1.68918410
C	-4.72420168	-6.49924490	1.51076545
C	-6.57578563	-4.86545827	1.07205133
C	3.28554190	-7.30135437	1.68874771
C	5.42532910	-6.11335986	1.14129134
C	2.28516039	-7.02902219	-1.03423830
C	4.73287742	-6.92210679	-1.57400702
C	6.13047959	5.69199141	-1.68924013

C	3.74620601	6.32417704	-1.23050646
C	6.57565498	4.86544105	1.07211876
C	4.72410200	6.49933830	1.51055324
H	-0.60309975	3.46866102	0.01997311
H	-1.33308655	-3.25830285	-0.02070209
H	1.33301318	3.25828841	-0.02071966
H	0.60302402	-3.46867175	0.01991415
H	-7.27041613	1.88713732	0.01641126
H	-8.86704750	0.53811621	-1.25652731
H	-7.33727386	0.66550837	-2.13648700
H	-7.74966667	-0.84014091	-1.28741270
H	-8.84474786	0.56369443	1.34687344
H	-7.30065654	0.71734120	2.19715508
H	-7.72197969	-0.80964533	1.39133983
H	7.27035342	-1.88713970	0.01666799
H	8.86707523	-0.53812186	-1.25617012
H	7.33737250	-0.66555708	-2.13624808
H	7.74967487	0.84011746	-1.28717349
H	8.84456784	-0.56366056	1.34722649
H	7.30041137	-0.71729470	2.19739123
H	7.72179199	0.80967631	1.39157758
H	-5.54286434	5.88436623	2.20414505
H	-5.89773669	5.31210639	0.57042040
H	-5.94402356	7.05441941	0.93617539

H	-3.53094244	7.12154227	2.73935688
H	-2.19842606	7.30725295	1.59173020
H	-3.66536772	8.29163535	1.41862514
H	-5.65620718	6.34924792	-1.48099105
H	-4.43487548	6.91969321	-2.62645843
H	-4.93193498	7.95840111	-1.28206971
H	-1.46169098	6.60212347	-0.45393560
H	-2.05347308	6.89931978	-2.09476586
H	-2.33783600	8.10070258	-0.82294448
H	-2.83993982	-6.10254115	-0.65939740
H	-3.51578228	-6.19862688	-2.29178186
H	-4.01609374	-7.37015969	-1.05932015
H	-6.91172915	-4.94370405	-1.54899952
H	-5.86268568	-5.71000170	-2.74950700
H	-6.53508349	-6.67480692	-1.42651077
H	-4.90004903	-6.31477467	2.57430938
H	-3.66619209	-6.73173544	1.37803339
H	-5.31321343	-7.37440623	1.21860527
H	-6.61819358	-4.65751306	2.14500024
H	-6.88113752	-3.96170736	0.54166672
H	-7.28617857	-5.66755369	0.85199641
H	2.19854176	-7.30738799	1.59149022
H	3.53093615	-7.12158095	2.73924004
H	3.66558525	-8.29163794	1.41849949

H	5.94417739	-7.05421489	0.93625480
H	5.89777426	-5.31188572	0.57059765
H	5.54280238	-5.88427199	2.20425669
H	2.05395377	-6.89948452	-2.09504265
H	1.46195294	-6.60245124	-0.45426347
H	2.33839886	-8.10086091	-0.82323453
H	4.43538466	-6.91939508	-2.62651545
H	5.65652463	-6.34882029	-1.48090885
H	4.93249487	-7.95810165	-1.28214338
H	5.86287359	5.70970520	-2.74958152
H	6.91179233	4.94343800	-1.54894461
H	6.53524298	6.67457583	-1.42661810
H	3.51594825	6.19856496	-2.29201116
H	2.84004814	6.10266354	-0.65963814
H	4.01633235	7.37014101	-1.05961836
H	7.28609491	5.66749368	0.85206098
H	6.88101713	3.96164001	0.54182491
H	6.61796841	4.65757224	2.14508608
H	3.66611993	6.73188292	1.37769190
H	4.89982183	6.31492501	2.57412848
H	5.31319692	7.37444666	1.21840161

Table S5. Cartesian coordinates for the optimized structure of **3’** in chloroform using B3LYP/6-31G(d,p) level.

Energy = -1168.2394 a.u.

C	-0.83605184	2.49811096	0.01145817
C	0.56052392	2.46885460	0.00940423
C	1.27377170	1.26670732	0.00191767
C	0.53953850	0.03743922	0.00001894
C	-0.89029318	0.07929645	0.00014711
C	-1.56846939	1.32068265	0.00586809
C	1.20030964	-1.23286816	-0.00204600
C	0.41772691	-2.39081224	-0.00962212
C	-0.97824751	-2.33819031	-0.01137817
C	-1.64016684	-1.12010331	-0.00550155
C	2.74451276	1.22261072	-0.00418401
C	3.40673552	-0.04714155	-0.00009962
C	2.67105886	-1.27574898	0.00397875
C	3.51894058	2.38080694	-0.01357070
C	4.92389354	2.33461234	-0.01575842
C	5.57684324	1.12240849	-0.00807536
C	4.83997424	-0.08924307	-0.00010286
C	5.50429288	-1.34208755	0.00784453
C	4.78127016	-2.51384013	0.01551545
C	3.37602702	-2.47756161	0.01335304
C	-3.11886715	-1.09517840	-0.00596976

N	-3.74466935	0.15987387	0.00053143
C	-3.04454073	1.38253574	0.00688787
O	-3.64419221	2.45580173	0.01309067
O	-3.78059730	-2.13163294	-0.01169394
C	-5.23878704	0.23261987	0.00141489
C	-5.83803460	-0.35008690	-1.28345673
C	-5.83693555	-0.36367571	1.28056714
H	1.08540001	3.41590281	0.01459457
H	0.88658172	-3.36667314	-0.01511962
H	3.04435080	3.35441993	-0.02022684
H	6.66199966	1.07632938	-0.00861823
H	6.59020079	-1.35978629	0.00840639
H	2.84542798	-3.42195698	0.02006376
H	-5.43413043	1.30405174	0.00715408
H	-6.91312454	-0.14396498	-1.30085270
H	-5.39234702	0.11927144	-2.16601709
H	-5.68890382	-1.42866958	-1.34451110
H	-6.91219567	-0.15870715	1.30066617
H	-5.39113631	0.09695952	2.16764736
H	-5.68690078	-1.44271752	1.33038026
H	-1.36670604	3.44353516	0.01714802
H	-1.56367027	-3.25070439	-0.01701523
H	5.48698042	3.26235519	-0.02312226
H	5.28902103	-3.47299678	0.02286516

Table S6. Cartesian coordinates for the optimized structure of **3b** in chloroform using B3LYP/6-31G(d,p) level.

Energy = -1989.660 a.u.

C	3.51984785	-2.67993581	-0.01440127
C	2.12523843	-2.60303159	-0.01333330
C	1.45425771	-1.37693460	-0.00537750
C	2.23017743	-0.17315694	0.00014440
C	3.65783934	-0.26425796	-0.00031163
C	4.29264905	-1.52845340	-0.00751965
C	1.61376642	1.11962452	0.00643781
C	2.43543336	2.25010860	0.01427230
C	3.82869344	2.14904700	0.01422473
C	4.44849625	0.90872070	0.00663467
C	-0.01451979	-1.28241671	-0.00210729
C	-0.63009149	0.00968567	0.00295836
C	0.14484237	1.21301804	0.00519464
C	-0.83105545	-2.40940135	-0.00299485
C	-2.24497499	-2.33758434	-0.00127052
C	-2.83610932	-1.08413343	0.00243428
C	-2.05997361	0.10079334	0.00522157
C	-2.67927359	1.37468692	0.00961641
C	-1.93357668	2.54274963	0.00790564
C	-0.52194694	2.43462402	0.00483428
C	5.92499313	0.83265577	0.00653978

N	6.50713912	-0.44348466	-0.00053105
C	5.76523277	-1.64149108	-0.00780733
O	6.32809427	-2.73478292	-0.01402505
O	6.62281107	1.84537372	0.01249132
C	7.99753974	-0.56827699	-0.00033350
C	8.61792350	0.00688004	-1.27863986
C	8.61627019	-0.00808334	1.28541730
B	-3.10587266	-3.63649371	-0.00532156
B	-2.62224418	3.94049874	0.00883400
O	-1.92311520	5.11933691	0.06446937
C	-2.87542979	6.20295142	-0.17824848
C	-4.25265917	5.52820973	0.19974336
O	-3.98174362	4.11190363	-0.04632065
O	-2.56409616	-4.89367142	-0.09165501
C	-3.64204798	-5.85167493	0.15319746
C	-4.92950778	-4.99999595	-0.18271465
O	-4.47473029	-3.63416389	0.07605110
C	-4.60707364	5.65322833	1.68638718
C	-5.43998964	5.94769198	-0.66270995
C	-2.47700824	7.39606693	0.68615615
C	-2.75823323	6.56185393	-1.66444230
C	-3.41700967	-7.06793825	-0.74096952
C	-3.54213702	-6.25271869	1.62981364
C	-5.32976657	-5.05389821	-1.66200188

C	-6.14107139	-5.27950915	0.70245023
H	4.01812482	-3.64277076	-0.02040642
H	1.56680871	-3.53061339	-0.01899919
H	1.99918151	3.24095788	0.02125907
H	4.44543296	3.04079605	0.02026264
H	-0.38779126	-3.39819949	-0.00427321
H	-3.91912802	-0.99448393	0.00194847
H	-3.76485292	1.42350280	0.01412327
H	0.04272324	3.35958628	0.00145138
H	8.15484049	-1.64595640	-0.00651586
H	9.68499016	-0.23734300	-1.29832184
H	8.15609284	-0.43648059	-2.16632560
H	8.50755374	1.09072063	-1.32796174
H	9.68327139	-0.25270852	1.30371093
H	8.15318416	-0.46166145	2.16727081
H	8.50605040	1.07514384	1.34724597
H	-4.88729660	6.67781788	1.94624711
H	-3.77234310	5.34919584	2.32402212
H	-5.45602271	4.99990313	1.90504235
H	-5.26851454	5.73591011	-1.71935592
H	-6.33298000	5.40090270	-0.34707116
H	-5.64217234	7.01728731	-0.54852708
H	-1.49845351	7.76747576	0.36906782
H	-2.41207576	7.12807845	1.74194020

H	-3.19851286	8.21164883	0.57614276
H	-1.72183274	6.83221233	-1.88378840
H	-3.03067164	5.71681544	-2.30277080
H	-3.39601552	7.41179620	-1.92266419
H	-2.48995560	-7.57005465	-0.45031711
H	-3.33567154	-6.78812112	-1.79255717
H	-4.23688274	-7.78482668	-0.63242527
H	-2.54546749	-6.65973468	1.82046691
H	-3.68894383	-5.39250461	2.28879898
H	-4.27978368	-7.01764996	1.88786561
H	-4.47766053	-4.84636213	-2.31517157
H	-6.09398842	-4.29512677	-1.85061190
H	-5.74286821	-6.03035491	-1.92983747
H	-5.92139446	-5.10424339	1.75684985
H	-6.96582223	-4.62208045	0.41326597
H	-6.47677165	-6.31429804	0.58258189

Table S7. Cartesian coordinates for the optimized structure of **3c** in chloroform using B3LYP/6-31G(d,p) level.

Energy = -1989.650 a.u.

C	-2.47719320	0.01939019	0.00071884
C	-2.49355604	1.42578451	0.00650802
C	-1.33370589	2.20518592	0.00982881
C	-0.06714908	1.54209390	0.01805613

C	-0.03440616	0.11649244	0.00857783
C	-1.23950578	-0.62302393	0.00114515
C	1.16766773	2.26269936	0.03521815
C	2.36210821	1.53753464	0.02504465
C	2.41015305	0.13176039	0.00863279
C	1.20347736	-0.56641523	0.00632679
C	-1.36720402	3.67638087	0.00379966
C	-0.13323436	4.40355164	0.03716960
C	1.13299434	3.73374600	0.06163084
C	-2.56341275	4.38973257	-0.03320252
C	-2.59029416	5.79516410	-0.03031376
C	-1.41429573	6.50996482	0.01146301
C	-0.16643118	5.83711208	0.04630695
C	1.04892307	6.56660208	0.09012085
C	2.25665837	5.90637514	0.12367816
C	2.29482177	4.50123760	0.10873240
C	1.24623641	-2.03816970	0.00201349
N	0.03513760	-2.73653487	-0.00299704
C	-1.21335018	-2.09411720	-0.00277513
O	-2.26192601	-2.74487168	-0.00479519
O	2.32493577	-2.63850440	0.00430805
C	0.04008639	-4.23337757	-0.00579204
C	0.66934629	-4.79599523	-1.28503565
C	0.65216833	-4.79974008	1.28013633

B	-3.84400213	-0.77195259	-0.00702190
B	3.81137039	-0.59688525	-0.00958448
O	-4.57239630	-0.98609588	1.13515978
C	-5.76205258	-1.74589646	0.75596834
C	-5.89414469	-1.42052989	-0.78665060
O	-4.52712309	-1.06630419	-1.15917546
O	4.56070996	-0.77321516	1.12580377
C	5.78359104	-1.47177566	0.73474135
C	5.88550434	-1.13698266	-0.80758376
O	4.49809853	-0.85831883	-1.16813966
C	-5.46090918	-3.22018358	1.05161084
C	-6.92684716	-1.26576492	1.61966586
C	-6.33940189	-2.59297584	-1.65802515
C	-6.75564774	-0.18670760	-1.08327607
C	6.67541552	0.14328964	-1.10718587
C	6.38878190	-2.28096160	-1.68517019
C	6.93066528	-0.93613142	1.58931694
C	5.55952226	-2.96009197	1.02815850
H	-3.45961494	1.91668331	0.00885205
H	3.30474861	2.07216787	0.02649494
H	-3.51059482	3.86534191	-0.06735595
H	-3.54551781	6.30953830	-0.06014650
H	-1.42448709	7.59599783	0.01669884
H	1.00888833	7.65198039	0.09841360

H	3.18711158	6.46387358	0.16067218
H	3.26512740	4.02065435	0.13714623
H	-1.01973307	-4.48463456	-0.01321000
H	0.52159144	-5.88049742	-1.30648795
H	0.18705844	-4.37372633	-2.17191753
H	1.73903019	-4.58898834	-1.33355652
H	0.50621833	-5.88458457	1.29586659
H	0.15680982	-4.38138434	2.16166604
H	1.72077403	-4.59120085	1.34432993
H	-5.22438969	-3.32406975	2.11439435
H	-4.60049685	-3.57260575	0.48015435
H	-6.32387567	-3.85519924	0.83102623
H	-6.72583786	-1.50278333	2.66828269
H	-7.07622279	-0.18763003	1.53916100
H	-7.85560059	-1.76898057	1.33244846
H	-5.65430516	-3.43806101	-1.57490919
H	-6.36856919	-2.28038413	-2.70579757
H	-7.34351275	-2.92658778	-1.37717039
H	-6.44355911	0.67237568	-0.48274800
H	-6.64278667	0.07899253	-2.13794851
H	-7.81487056	-0.37835956	-0.88948100
H	6.32057567	0.98260303	-0.50237198
H	6.54070557	0.40459383	-2.16037171
H	7.74496249	0.01006815	-0.92107439

H	5.75230746	-3.16294346	-1.59962902
H	6.39313846	-1.96494470	-2.73231013
H	7.41180975	-2.55901750	-1.41211116
H	6.75260560	-1.18696708	2.63886174
H	7.02396881	0.14850316	1.51179896
H	7.88088934	-1.39027581	1.29074058
H	5.33480005	-3.07852041	2.09197764
H	4.71504447	-3.35430947	0.46018910
H	6.45233461	-3.54984766	0.80078568

Table S8. Cartesian coordinates for the optimized structure of **3a** in chloroform using B3LYP/6-31G(d,p) level.

Energy = -2811.071 a.u.

C	2.18551627	-2.45925543	0.03661306
C	2.34038619	-1.06137853	0.02763901
C	1.26255883	-0.17212749	0.01154710
C	-0.06361359	-0.70719105	0.00819835
C	-0.23625978	-2.12283652	0.01133613
C	0.89049755	-2.97706978	0.02302200
C	-1.22233401	0.13109944	-0.00033560
C	-2.48221504	-0.47302313	-0.01295286
C	-2.66765846	-1.86727854	-0.01186211
C	-1.53534879	-2.68075406	0.00022115
C	1.44060893	1.28906970	-0.00268557

C	0.28274751	2.13161402	-0.00160190
C	-1.04371669	1.59231495	0.00376962
C	2.69819537	1.88492960	-0.01831653
C	2.88704227	3.28801386	-0.02728369
C	1.76357860	4.09919535	-0.01831694
C	0.45633894	3.55396456	-0.00615795
C	-0.68136618	4.39767583	0.00117389
C	-1.96699108	3.88060666	0.01321069
C	-2.12109146	2.47323049	0.01373498
C	-1.72249333	-4.14066373	-0.00339339
N	-0.58579530	-4.95455782	0.00883395
C	0.71993840	-4.43792818	0.01513725
O	1.69921508	-5.18926993	0.01278430
O	-2.85478671	-4.63277720	-0.01819652
C	-0.73729776	-6.44335320	0.00718187
C	-1.43138555	-6.94449136	1.27852299
C	-1.38802629	-6.94564629	-1.28642050
B	3.46710311	-3.38212120	0.05810715
B	-4.13359364	-2.45462167	-0.03023567
B	4.32114781	3.89640161	-0.04782578
B	-3.21212298	4.81704430	0.02641847
O	4.20011575	-3.63679109	-1.07293506
C	5.29873943	-4.52118155	-0.69058859
C	5.41927711	-4.25984293	0.86577221

O	4.08696710	-3.77636838	1.21618351
O	-4.86545242	-2.55095580	-1.18668030
C	-6.16219551	-3.12516996	-0.83539489
C	-6.27133535	-2.79676521	0.70863174
O	-4.87413666	-2.65339528	1.10739875
O	-4.50081716	4.35281506	-0.04826411
C	-5.38884148	5.48459092	0.21534829
C	-4.46543793	6.72085439	-0.12311839
O	-3.12663385	6.18304626	0.11517562
O	5.45966498	3.13433514	0.02219758
C	6.59358599	4.01770103	-0.24805346
C	5.99827920	5.44024333	0.09426630
O	4.56818640	5.24242714	-0.13928220
C	4.86379690	-5.94833932	-1.04548363
C	6.52819529	-4.13023182	-1.50856930
C	5.71444927	-5.49982387	1.70730610
C	6.39583298	-3.13349160	1.22677127
C	-6.94515071	-1.45081507	1.00413398
C	-6.90144999	-3.89704581	1.56003641
C	-7.22565149	-2.46674256	-1.71232342
C	-6.08290364	-4.62539647	-1.14321293
C	-4.51339050	7.13466211	-1.59892793
C	-4.66078146	7.94022477	0.77393746
C	-6.62744682	5.33976463	-0.66474511

C	-5.77725700	5.39848547	1.69627967
C	7.76552261	3.57739952	0.62496330
C	6.94063789	3.84055907	-1.73116454
C	6.15017752	5.82776792	1.57016927
C	6.47947955	6.57807099	-0.80209627
H	3.34953795	-0.66678014	0.03460749
H	-3.36722753	0.15220823	-0.02507792
H	3.58901702	1.26794113	-0.02604251
H	1.87452107	5.18021478	-0.02081249
H	-0.52904476	5.47364568	-0.00292630
H	-3.13395948	2.08821575	0.02455791
H	0.29288031	-6.79652372	0.02461750
H	-1.38961340	-8.03826819	1.29857791
H	-0.91957030	-6.57246515	2.17134426
H	-2.47641608	-6.63493532	1.31717133
H	-1.34852202	-8.03956483	-1.30337629
H	-0.84486141	-6.57631174	-2.16166957
H	-2.43044461	-6.63372366	-1.36146308
H	4.65114697	-5.99475372	-2.11741733
H	3.95572630	-6.23147688	-0.51027094
H	5.65283087	-6.67263073	-0.82305839
H	6.33228123	-4.30899718	-2.56964024
H	6.78147185	-3.07592104	-1.38430317
H	7.39446321	-4.73380401	-1.21944197

H	4.94821818	-6.26563601	1.57719773
H	5.74451003	-5.22529077	2.76565233
H	6.68643712	-5.92770739	1.44167500
H	6.18948534	-2.22628980	0.65201708
H	6.28357385	-2.89359653	2.28769003
H	7.43435487	-3.42762349	1.04972791
H	-6.49496095	-0.64225939	0.42137635
H	-6.81673099	-1.21505135	2.06412325
H	-8.01670554	-1.47987912	0.78658173
H	-6.34936434	-4.83460147	1.47772677
H	-6.90044873	-3.59302180	2.61075007
H	-7.93947741	-4.07417043	1.26072077
H	-7.04485655	-2.72304237	-2.76010850
H	-7.21060253	-1.37914041	-1.62277877
H	-8.22485884	-2.82528938	-1.44519984
H	-5.84789857	-4.75473458	-2.20354763
H	-5.29483960	-5.10872278	-0.56302262
H	-7.03526420	-5.12456661	-0.94199047
H	-5.46557589	7.60958667	-1.85143213
H	-4.36724586	6.27620601	-2.26033784
H	-3.71108457	7.85176813	-1.79229868
H	-4.48312646	7.70266467	1.82406631
H	-3.96069568	8.72750351	0.48045724
H	-5.67602439	8.33626594	0.67226286

H	-7.18123653	4.44281643	-0.37342649
H	-6.36597151	5.24886096	-1.72026805
H	-7.29189178	6.20080412	-0.54216498
H	-6.24046469	4.42640959	1.88572152
H	-4.90189919	5.48903522	2.34534869
H	-6.49366224	6.17856444	1.96850386
H	8.08414537	2.57314988	0.33165373
H	7.49619971	3.55238949	1.68212125
H	8.61771341	4.25216324	0.49721638
H	7.15210993	2.78508162	-1.92255478
H	6.10989607	4.14186898	-2.37523673
H	7.82383244	4.42283510	-2.00828854
H	5.80278499	5.02919675	2.23150849
H	5.54631262	6.71767117	1.76719813
H	7.19001172	6.05724601	1.81912748
H	6.25007407	6.39134011	-1.85246214
H	5.98796205	7.50954153	-0.50739662
H	7.55990762	6.72027653	-0.70000901

S.3. OPTOELECTRONIC MEASUREMENTS

The absorption and fluorescence emission spectra of **1’** and **1a** (λ_{ex} =410 nm); and **2’**, **2a**, **3’**, **3a**, **3b** and **3c** (λ_{ex} =480 nm) were recorded at 25 ºC (c = 2 µM).

- Fluorescence quantum yields were calculated for solutions with absorbance < 0.1. They were evaluated by comparison with a standard, whether Rhodamine 6G (EtOH, Φ_R = 95%, λ_{ex} = 480 nm) or perylene (Cyclohexane, Φ_R = 94%, λ_{ex} = 410 nm), using equation (S1):

$$\Phi = \Phi_R \frac{Int}{Int_R}\left(\frac{A_R}{A}\right)\frac{n^2}{n_R^2} \qquad (S1)$$

where Φ_R is the quantum yield of the standards, Int is the area of the emission intensity of the sample, Int_R is the area of the emission intensity of the standard, A is the absorbance, A_R is the absorbance of the standard, n is the refractive index of the sample and n_R is the refractive index of the standard.

- Absorption spectra were deconvoluted using fityk software and optimized to four (**1a-1’** and **2a-2’**) or three (**3’**, **3a**, **3b** and **3c**) gaussian components.
- TD-DFT calculations were carried out at the CAM-B3LYP/6-31+G(d,p) level of theory. From the previously optimized structures.

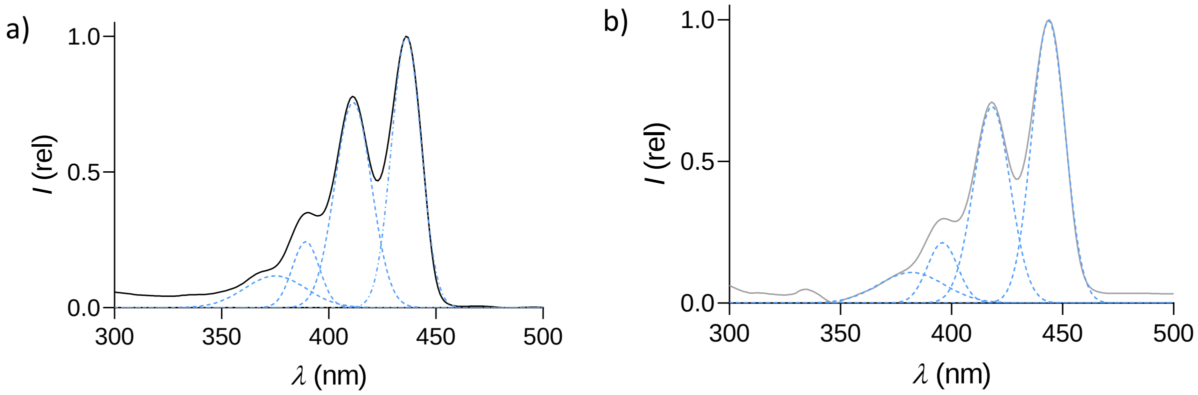

Figure S19 Deconvoluted normalized absorption spectra of **1’** (a) and **1** (b) in $CHCl_3$.

Table S9 Selected transition properties of **2** calculated at CAM-B3LYP/6-31+G(d,p) level of theory.

**Wavelength (nm)**[a]	**Osc. Strength**[b]	**Major contributions**[c]
399.5	0.561	H->L (98%)
242.7	2.085	H-2->L (58%), H->L+3 (38%)
222.4	0.630	H-1->L+1 (54%)
219.1	0.396	H-4->L+1 (38%), H->L+5 (23%)
205.1	1.289	H-3->L+1 (88%)

[a]Wavelength associated to the transition. [b]Oscillator strength. [c]MOs involved in the transitions (H = HOMO and L= LUMO).

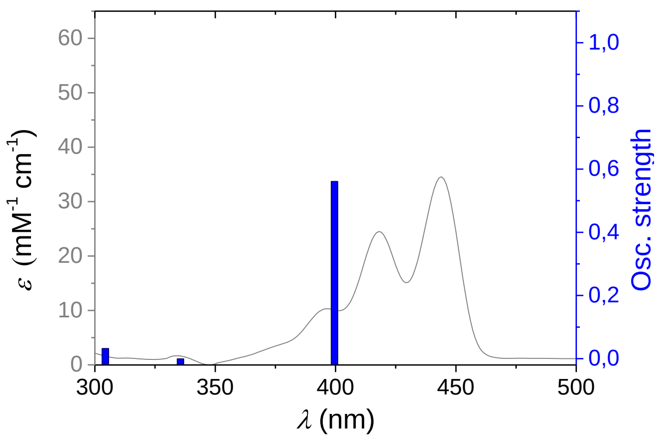

Figure S20 Molar extinction coefficient spectra vs calculated oscillator strength and their contribution (blue bars, from Table 1) at different wavelengths for compound **1a**.

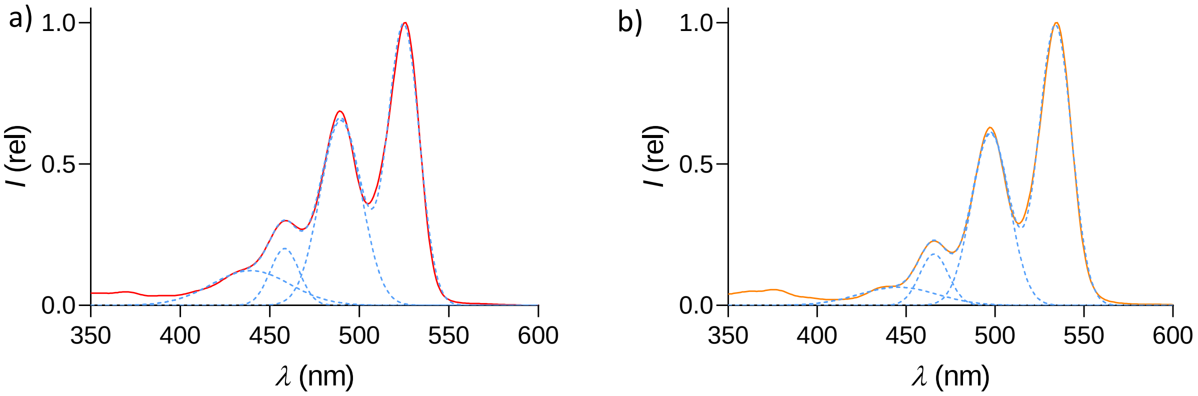

Figure S21 Deconvoluted normalized absorption spectra of **2’** (a) and **2a** (b) in $CHCl_3$.

Table S10 Selected transition properties of **2** calculated at CAM-B3LYP/6-31+G(d,p) level of theory.

**Wavelength (nm)**[a]	**Osc. Strength**[b]	**Major contributions**[c]
486.3	1.009	H->L (98%)
322.4	0.212	H-2->L (79%)
262.3	0.073	H-11->L (38%), H-5->L (29%)

[a]Wavelength associated to the transition. [b]Oscillator strength. [c]MOs involved in the transitions (H = HOMO and L= LUMO).

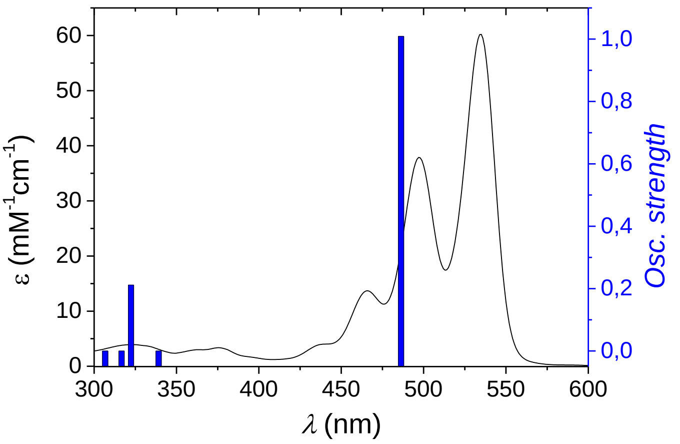

Figure S22 Molar extinction coefficient spectra vs calculated oscillator strength and their contribution (blue bars, from Table 1) at different wavelengths for compound **2a**.

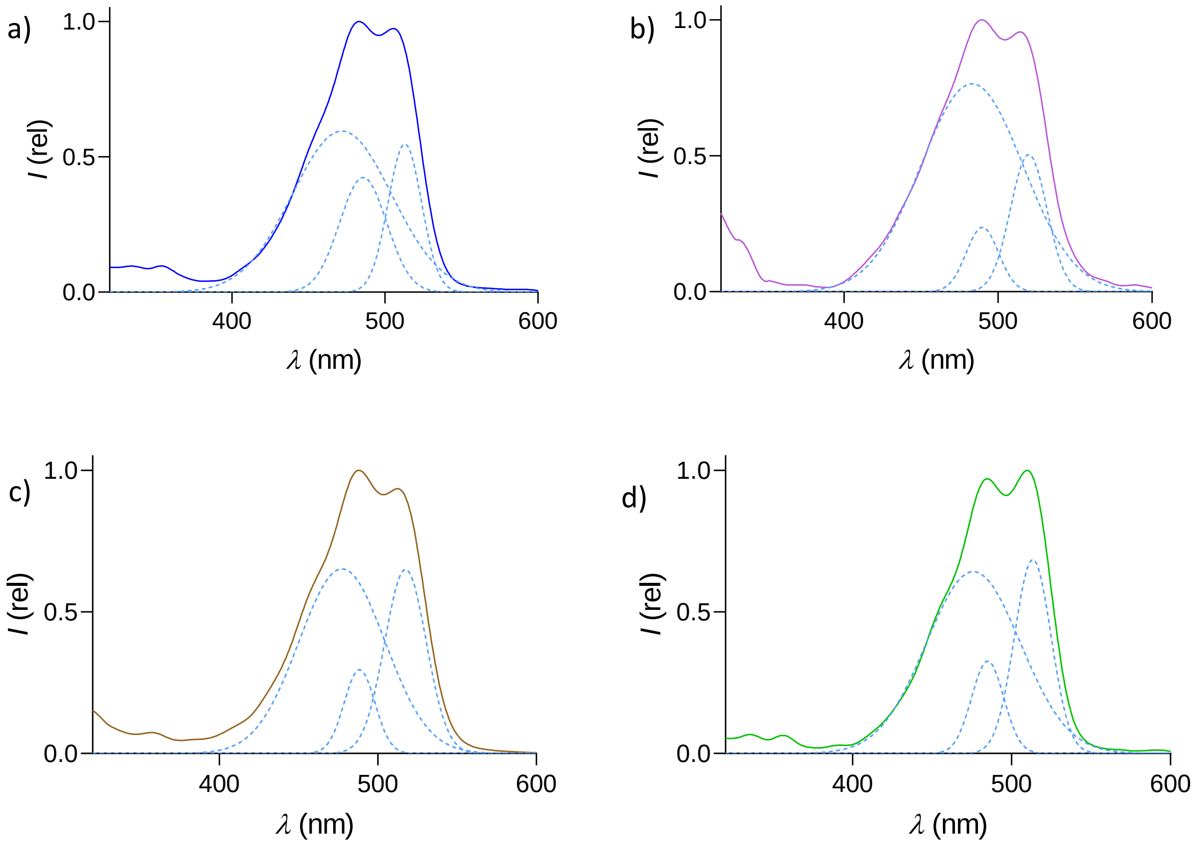

Figure S23 Deconvoluted normalized absorption spectra of **3’** (a), **3a** (b), **3b** (c) and **3c** (d) in $CHCl_3$.

Table S11 Selected transition properties of **2** calculated at CAM-B3LYP/6-31+G(d,p) level of theory.

**Cpd**	**Wavelength (nm)**[a]	**Osc. Strength**[b]	**Major contributions**[c]
	455.6	0.823	H->L (98%)
	309.2	0.053	H-2->L (64%)
**3a**	275.5	0.067	H-4->L (54%), H->L+3 (29%)
	248.7	0.394	H->L+4 (32%)
	235.7	1.014	H-5->L (42%), H->L+4 (31%)
	456.2	0.791	H->L (98%)
**3b**	232.5	0.698	H-5->L (38%), H->L+4 (40%)
	235.7	0.439	H-8->L (53%)

	448.3	0.86	H->L (98%)
	271.6	0.114	H-1->L (32%), H->L+1 (21%), H->L+3 (28%)
**3c**	248	0.258	H-6->L (43%), H->L+4 (22%)
	234	0.904	H-6->L (44%), H->L+4 (40%)
	219.3	0.062	H-4->L+1 (21%), H-1->L+1 (23%)
	235.7	0.382	H-1->L+1 (46%)

[a]Wavelength associated to the transition (nm). [b]Oscillator strength. [c]MOs involved in the transitions (H = HOMO and L= LUMO).

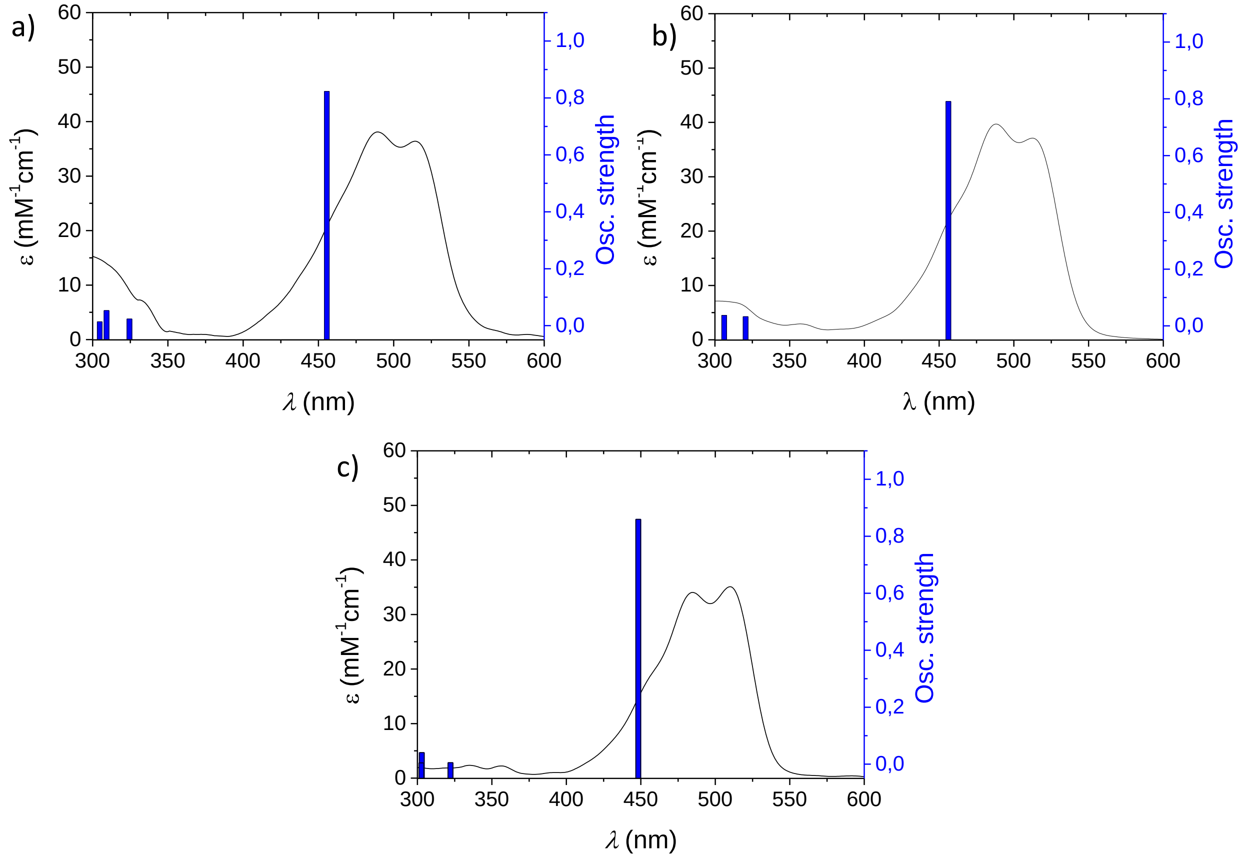

Figure S24 Molar extinction coefficient spectra vs calculated oscillator strength and their contribution (blue bars, from Table 3) at different wavelengths for compounds **3a** (a), **3b** (b) and **3c** (c).

S.4. NMR CHARACTERIZATION

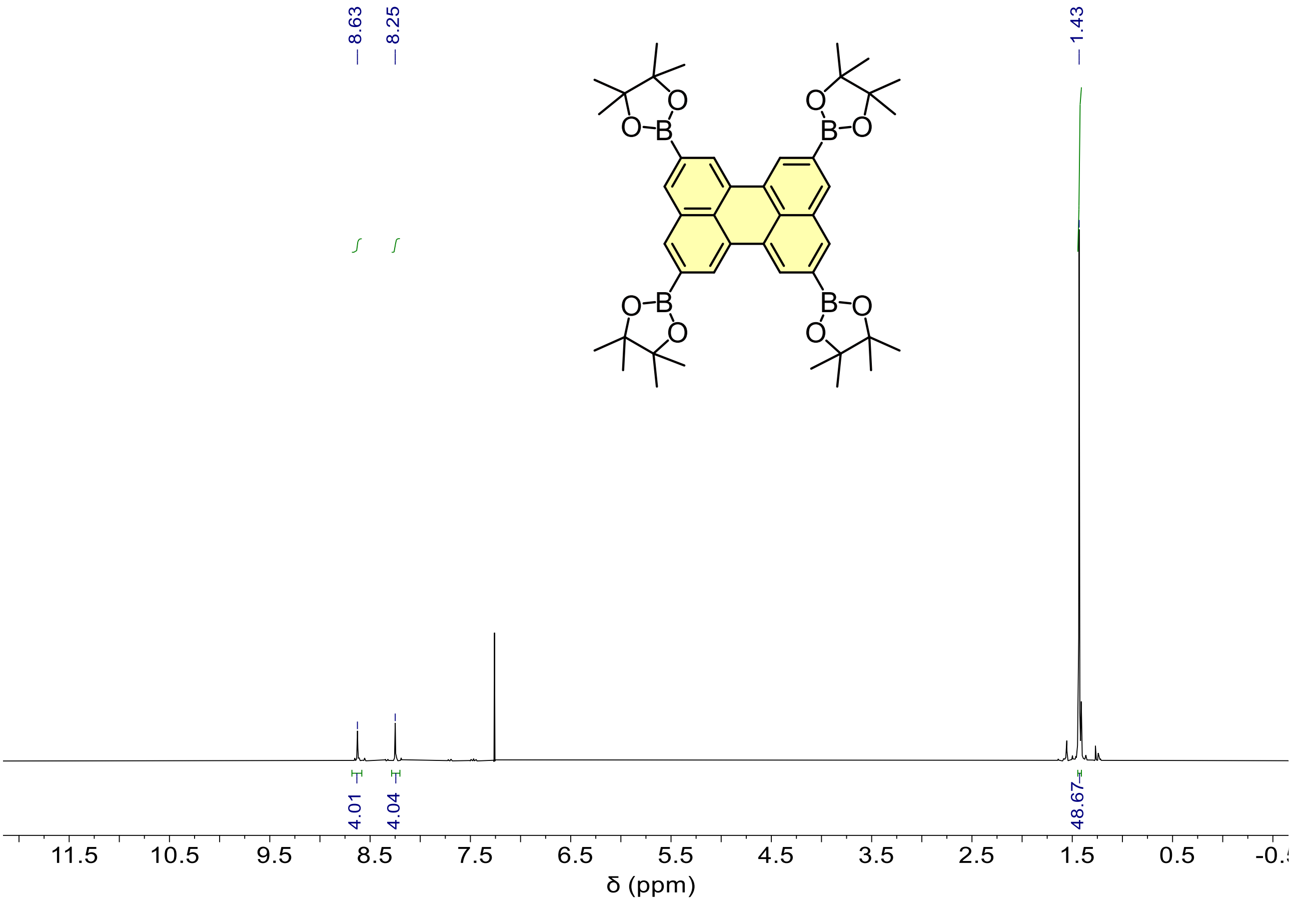

Figure S25 ^{1}H NMR (300 MHz) spectrum of compound **1a** in $CDCl_3$.

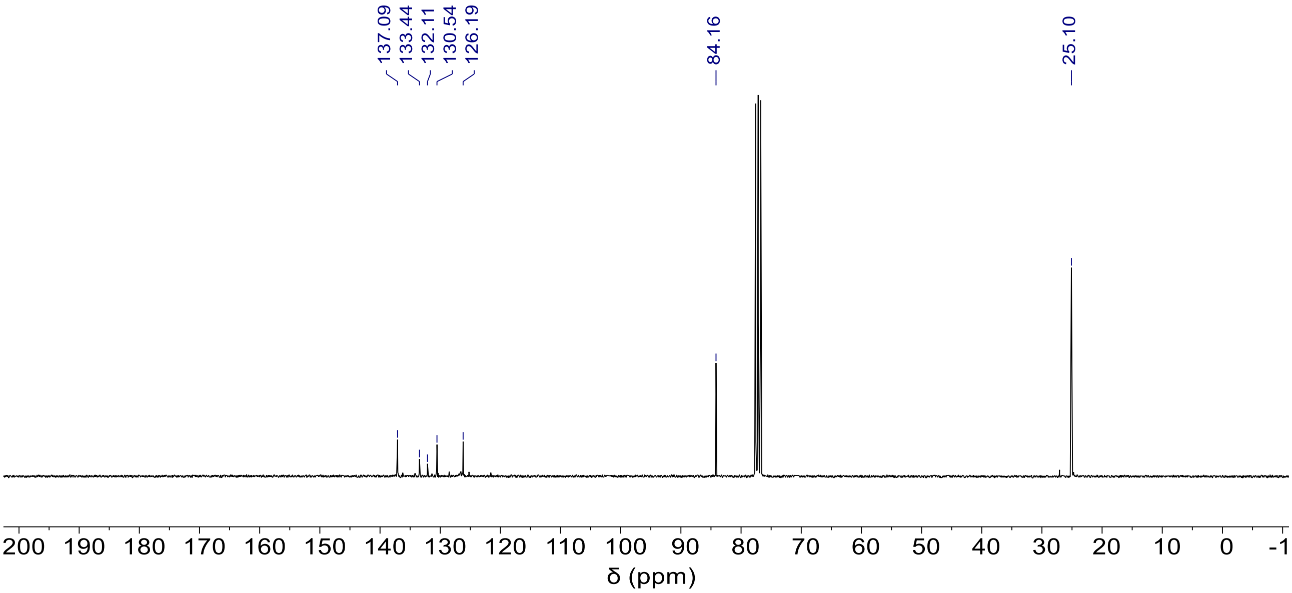

Figure S26 ^{13}C{1H} NMR (75 MHz) spectrum of compound **1a** in $CDCl_3$.

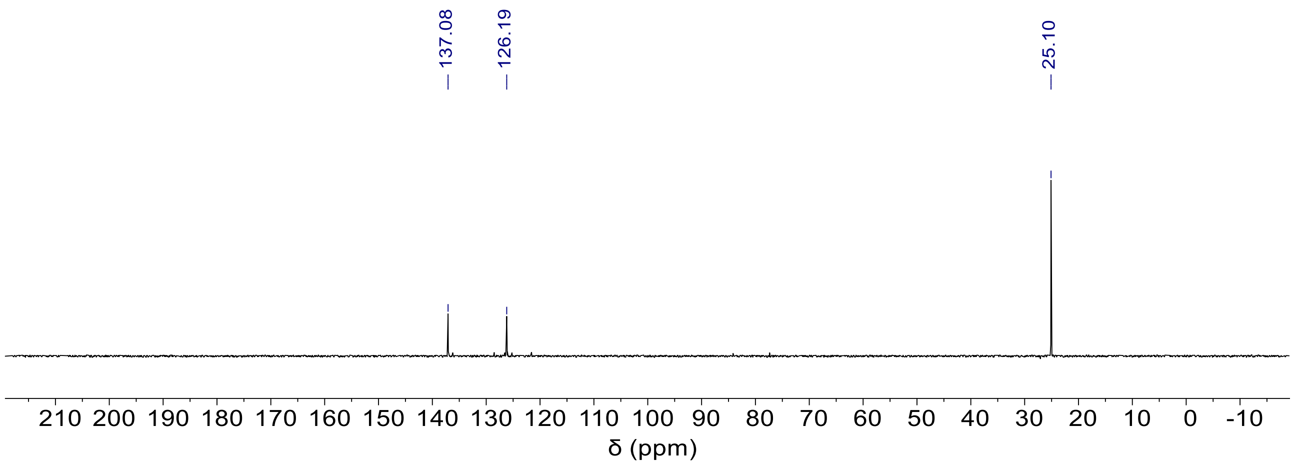

Figure S27 DEPT NMR (75 MHz) spectrum of compound **1a** in $CDCl_3$.

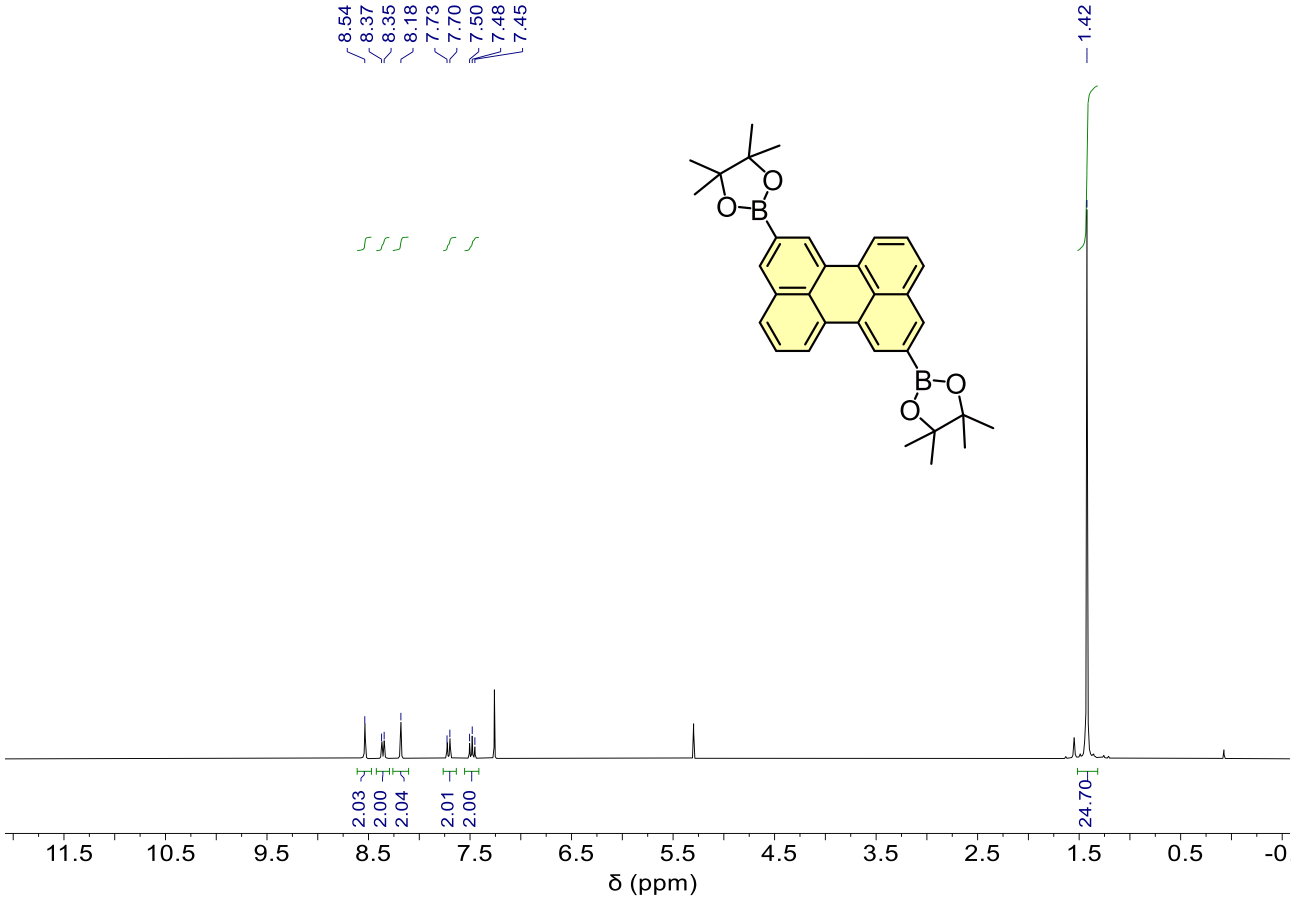

Figure S28 ^{1}H NMR (300 MHz) spectrum of compound **1b** in $CDCl_3$.

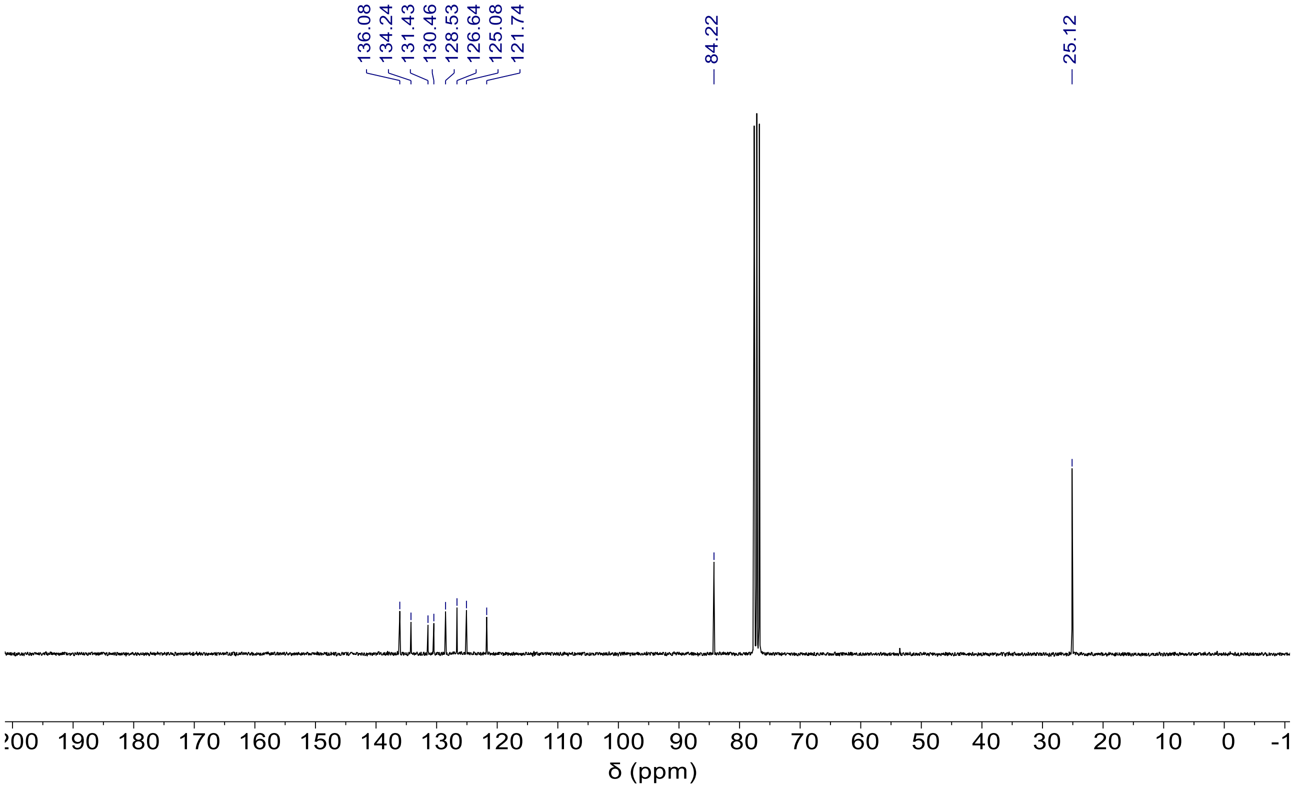

Figure S29 $^{13}C\{1H\}$ NMR (75 MHz) spectrum of compound **1b** in $CDCl_3$.

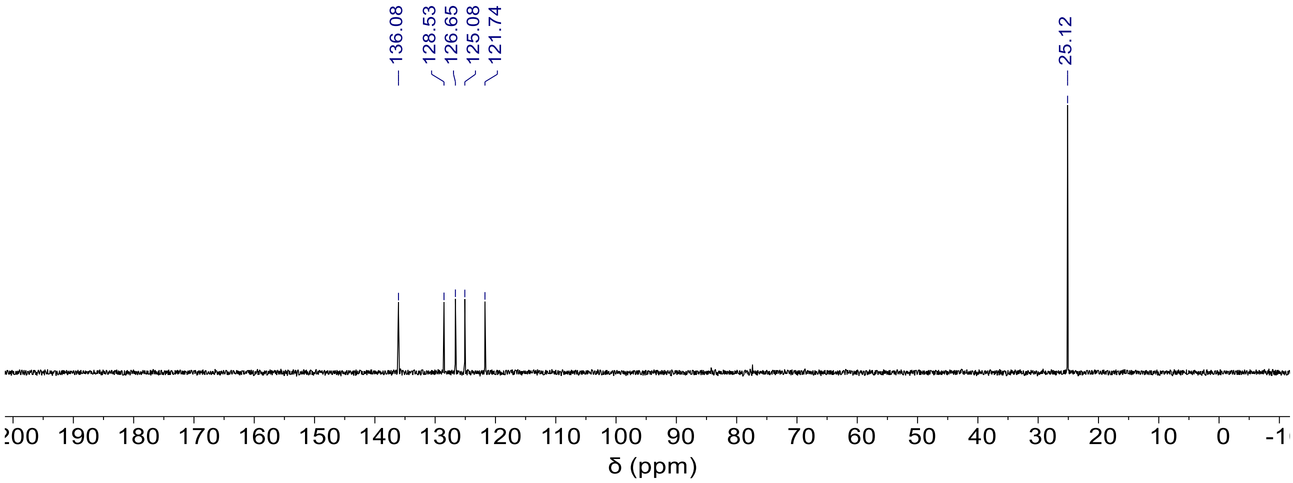

Figure S30 DEPT{1H} NMR (75 MHz) spectrum of compound **1b** in $CDCl_3$.

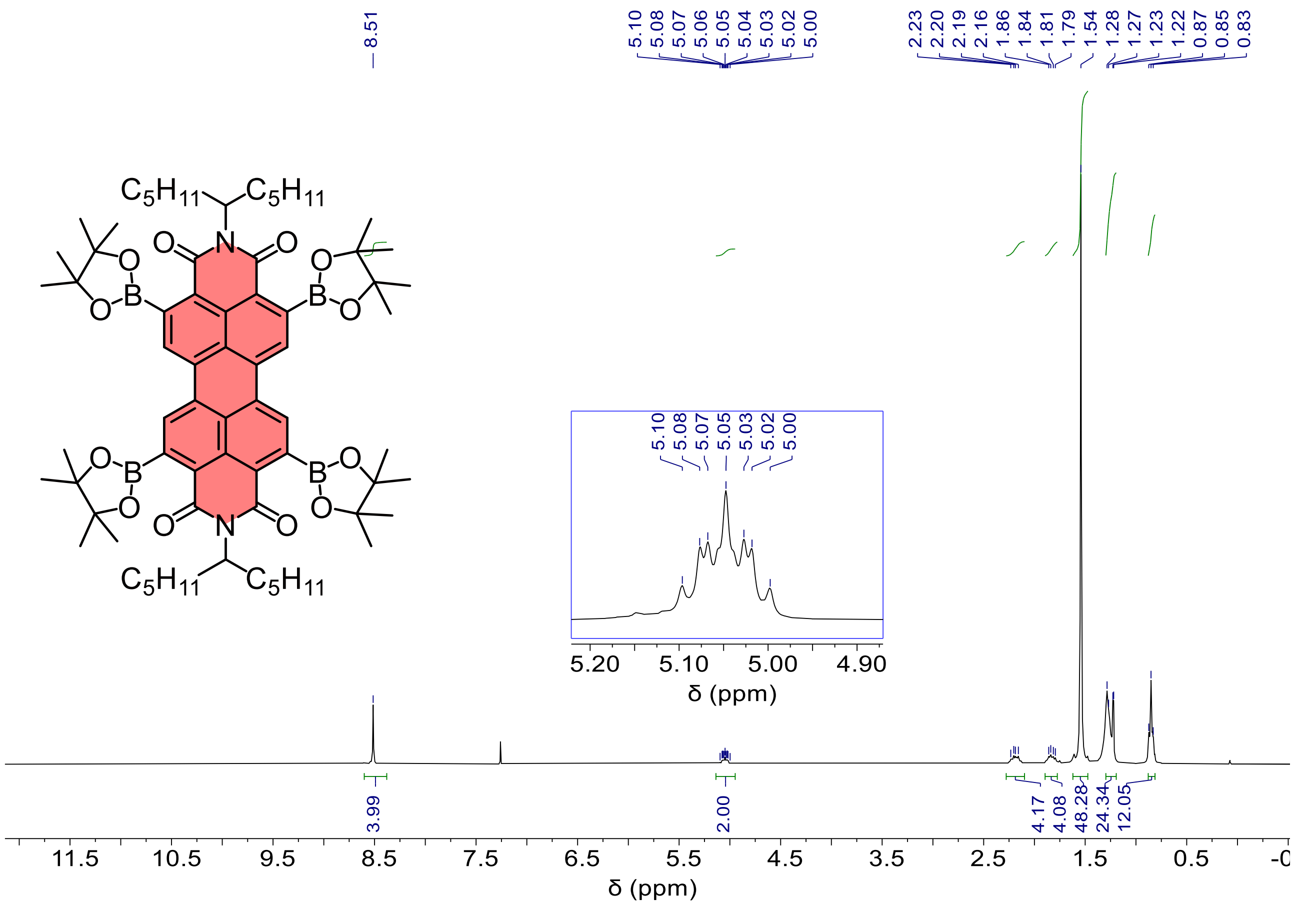

Figure S31 ^{1}H NMR (300 MHz) spectrum of compound **2a** in $CDCl_3$.

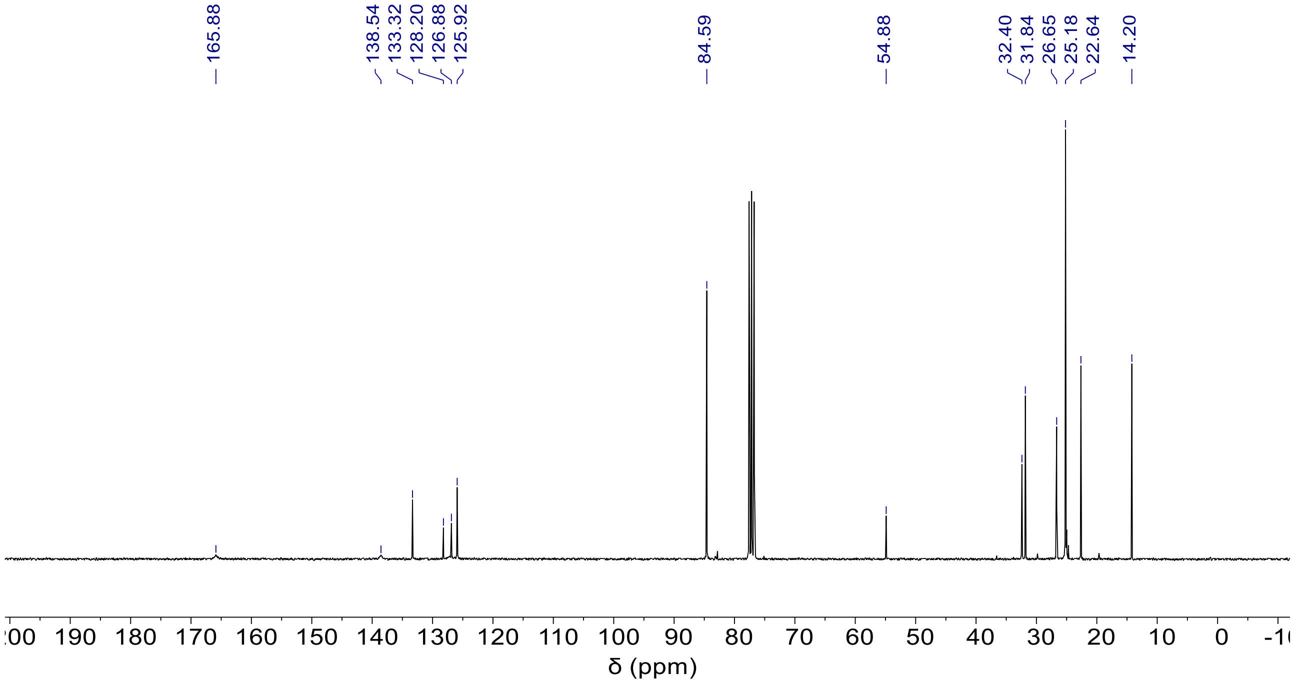

Figure S32 ^{13}C{1H} NMR (75 MHz) spectrum of compound **2a** in $CDCl_3$.

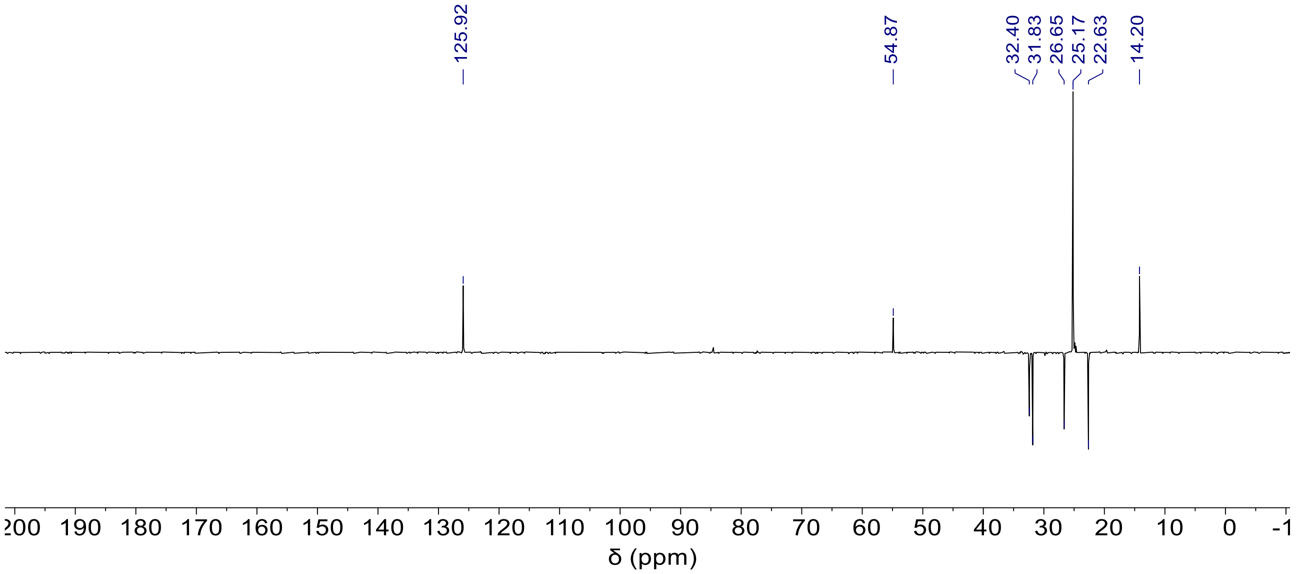

Figure S33 DEPT{1H} NMR (75 MHz) spectrum of compound **2a** in $CDCl_3$.

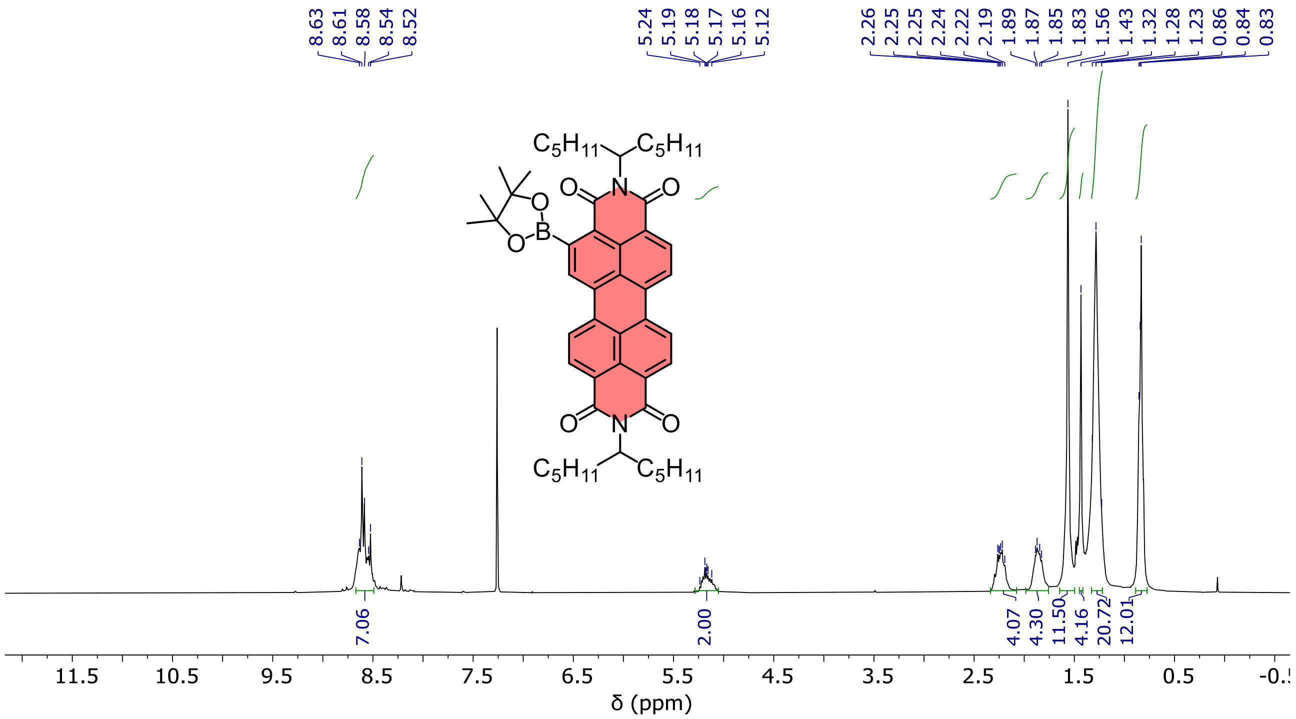

Figure S34 ^1H NMR (300 MHz) spectrum of compound **2b** in $CDCl_3$.

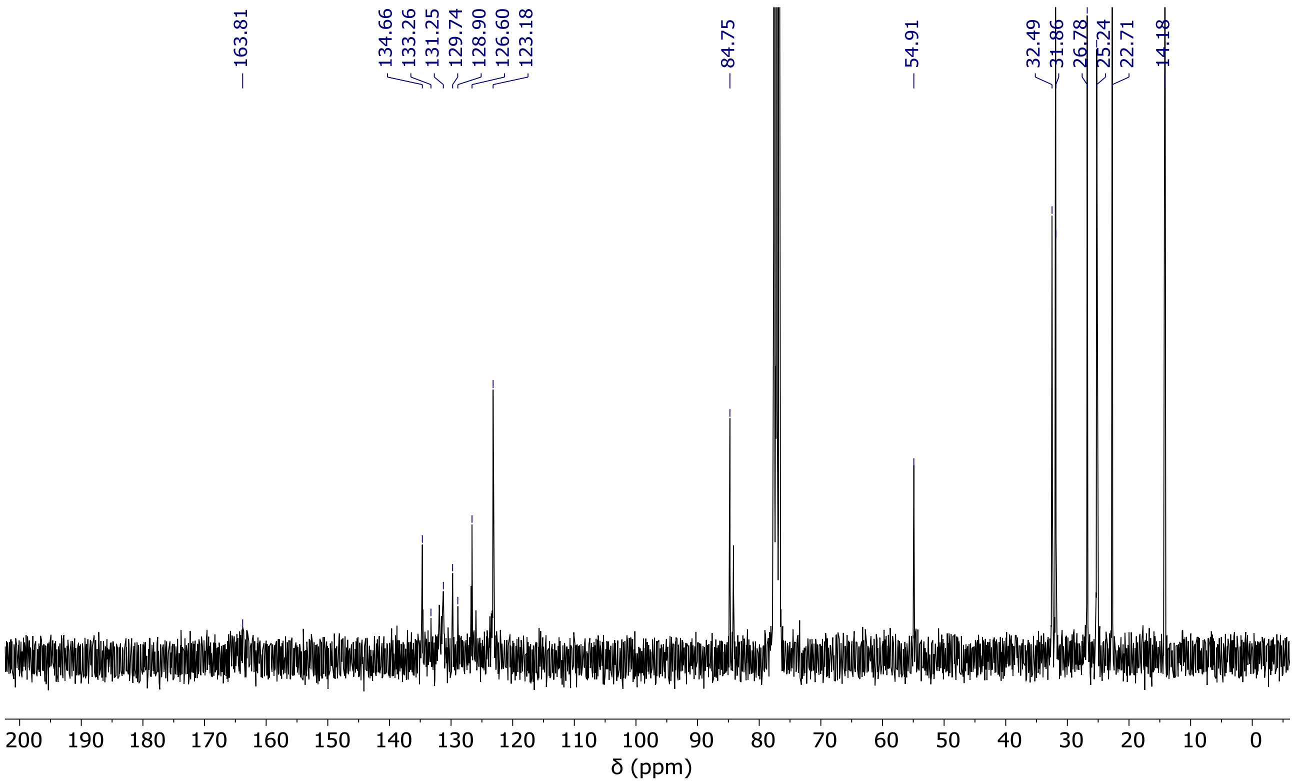

Figure S35 $^{13}C\{1H\}$ NMR (75 MHz) spectrum of compound **2b** in $CDCl_3$.

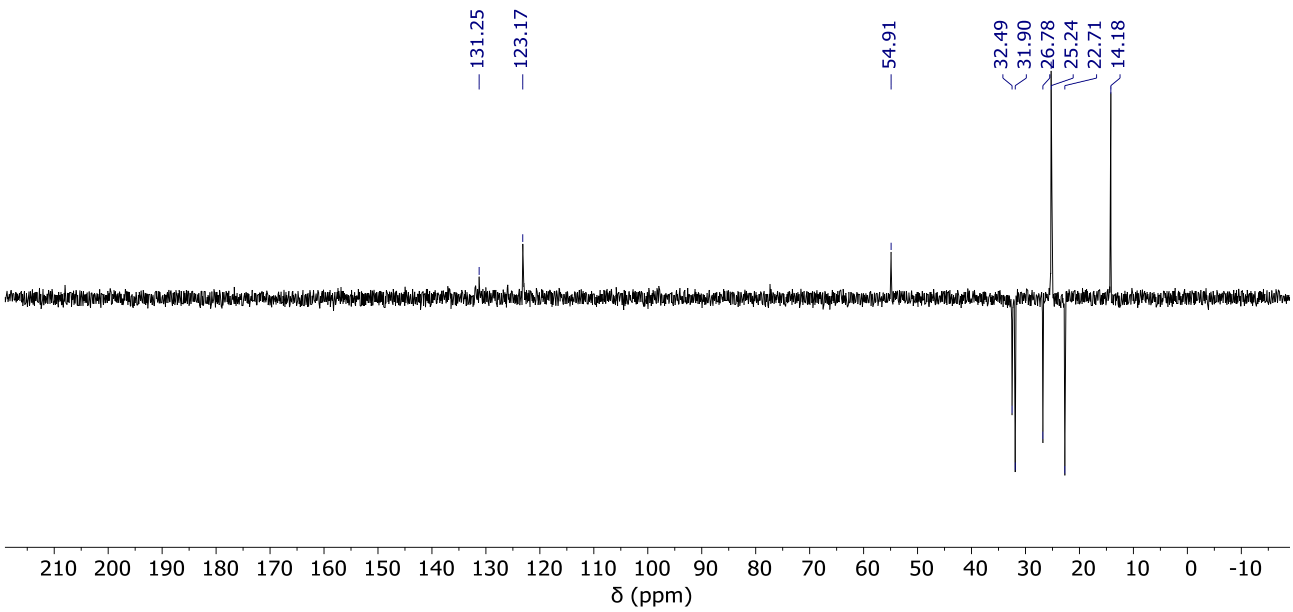

Figure S36 DEPT{1H} (75 MHz) spectrum of compound **2b** in $CDCl_3$.

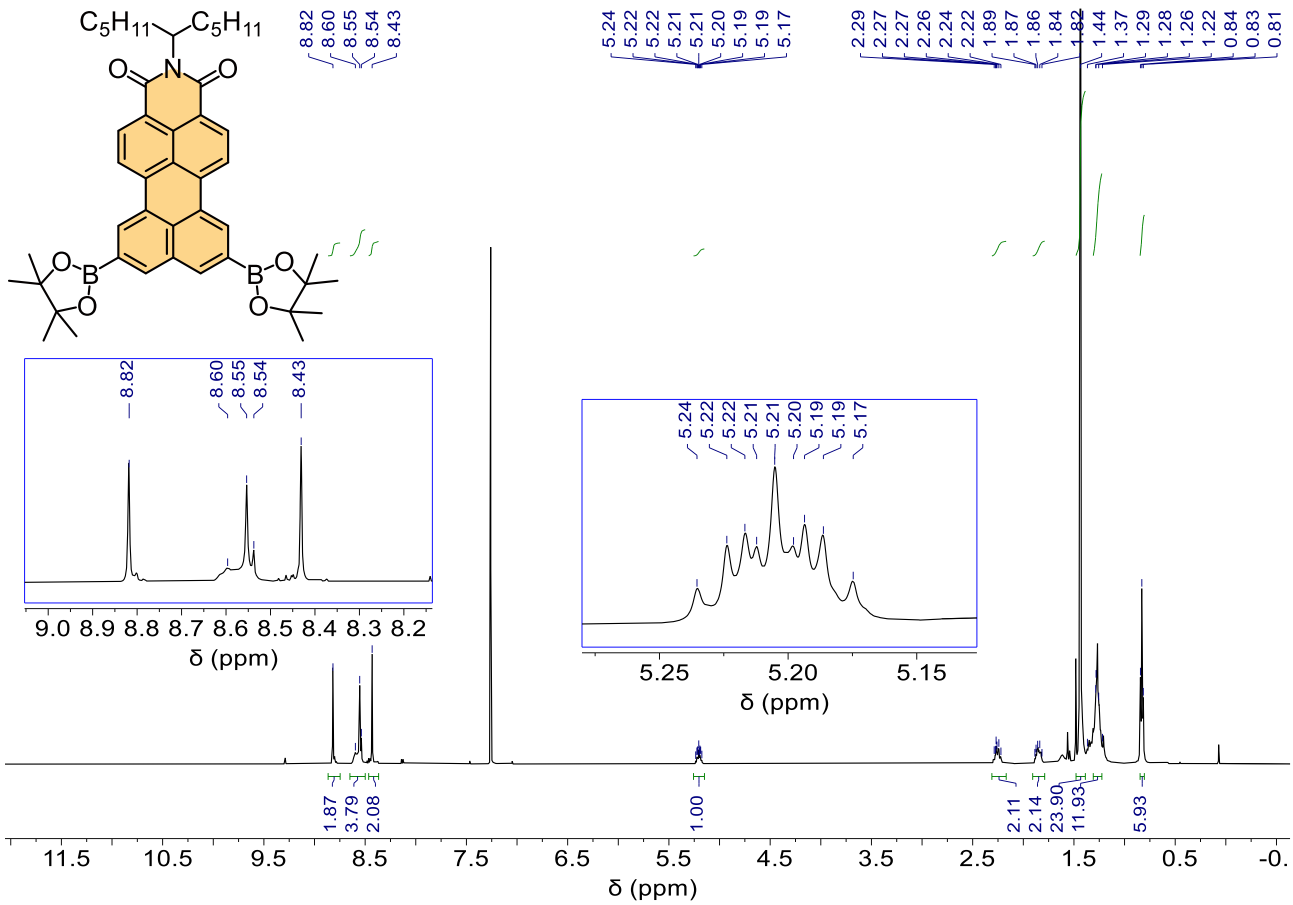

Figure S37 ^{1}H NMR (500 MHz) spectrum of compound **3b** in $CDCl_3$.

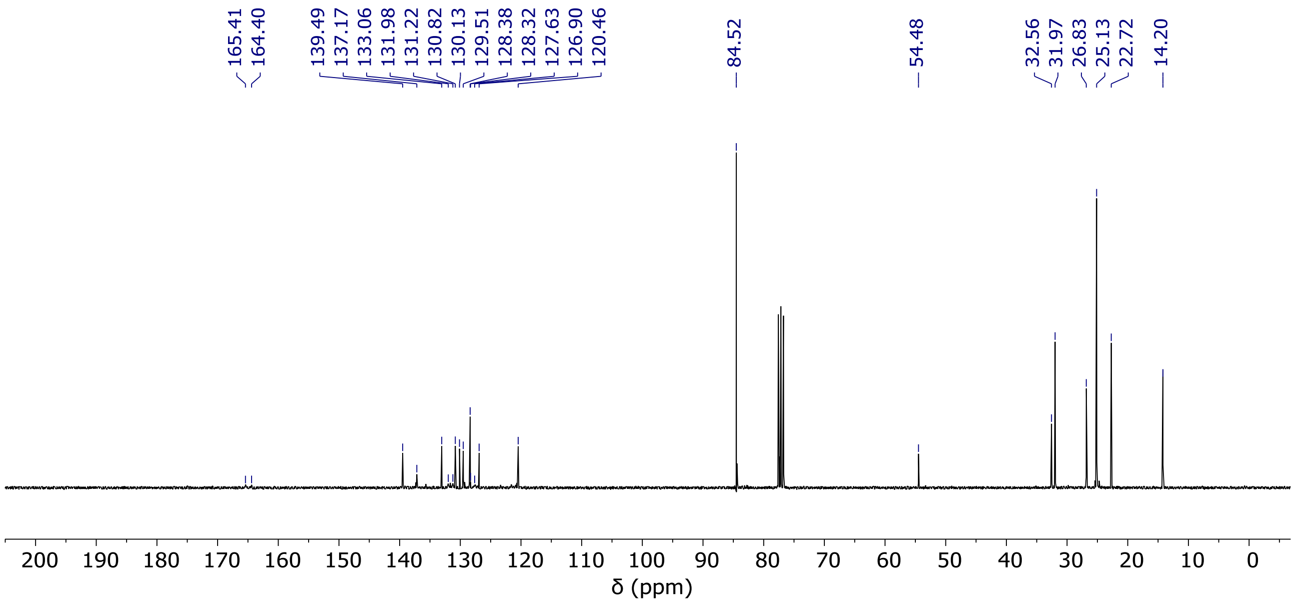

Figure S38 $^{13}C\{1H\}$ NMR (126 MHz) spectrum of compound **3b** in $CDCl_3$.

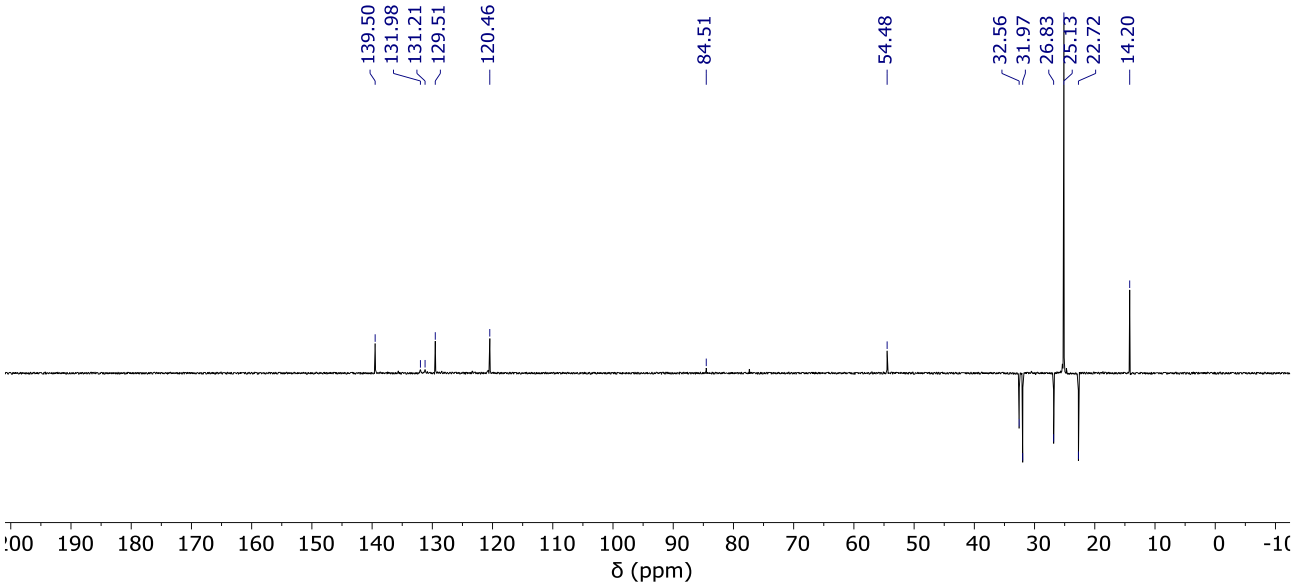

Figure S39 DEPT{1H} (126 MHz) spectrum of compound **3b** in $CDCl_3$.

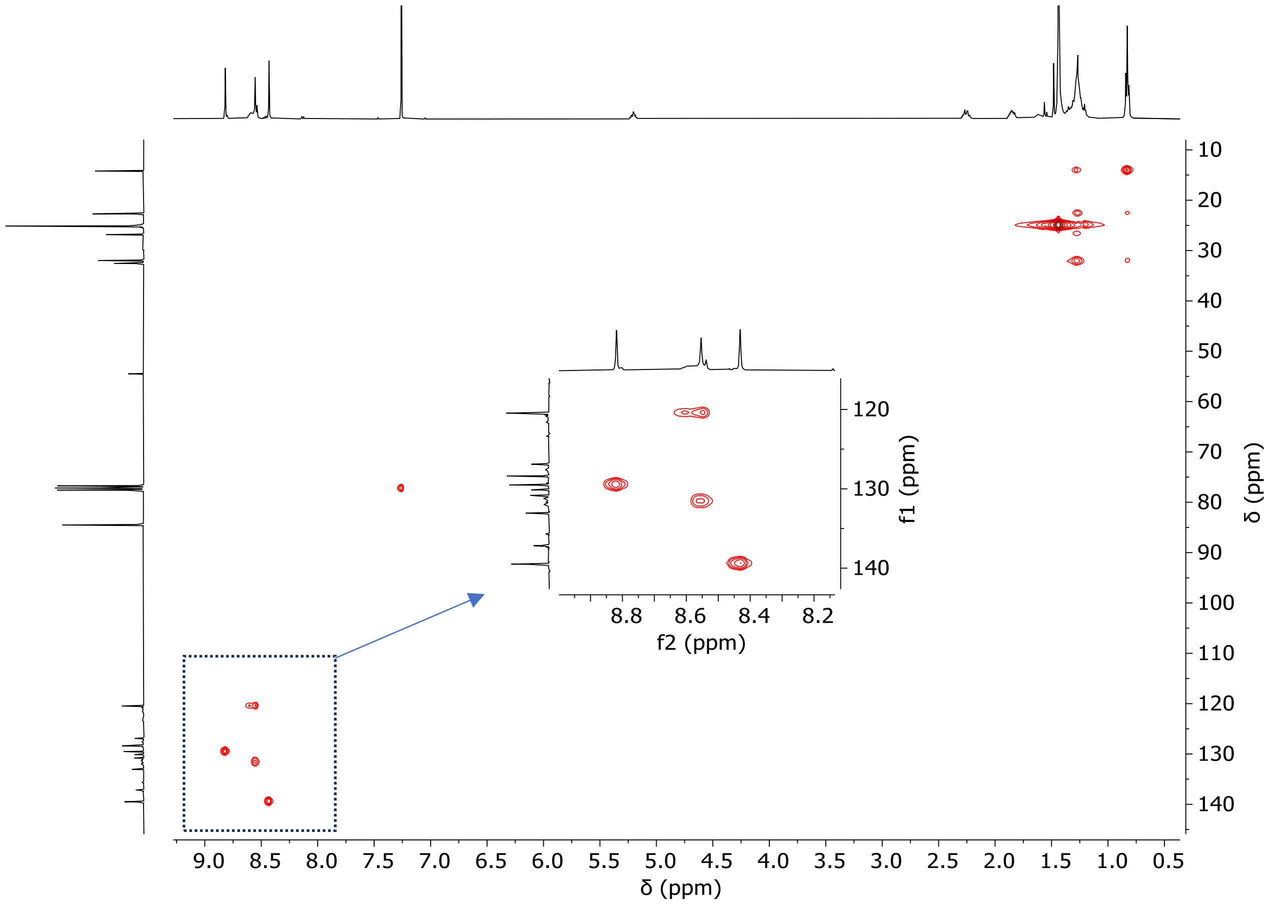

Figure S40 HMQC{1H} (500 MHz) spectrum of compound **3b** in $CDCl_3$.

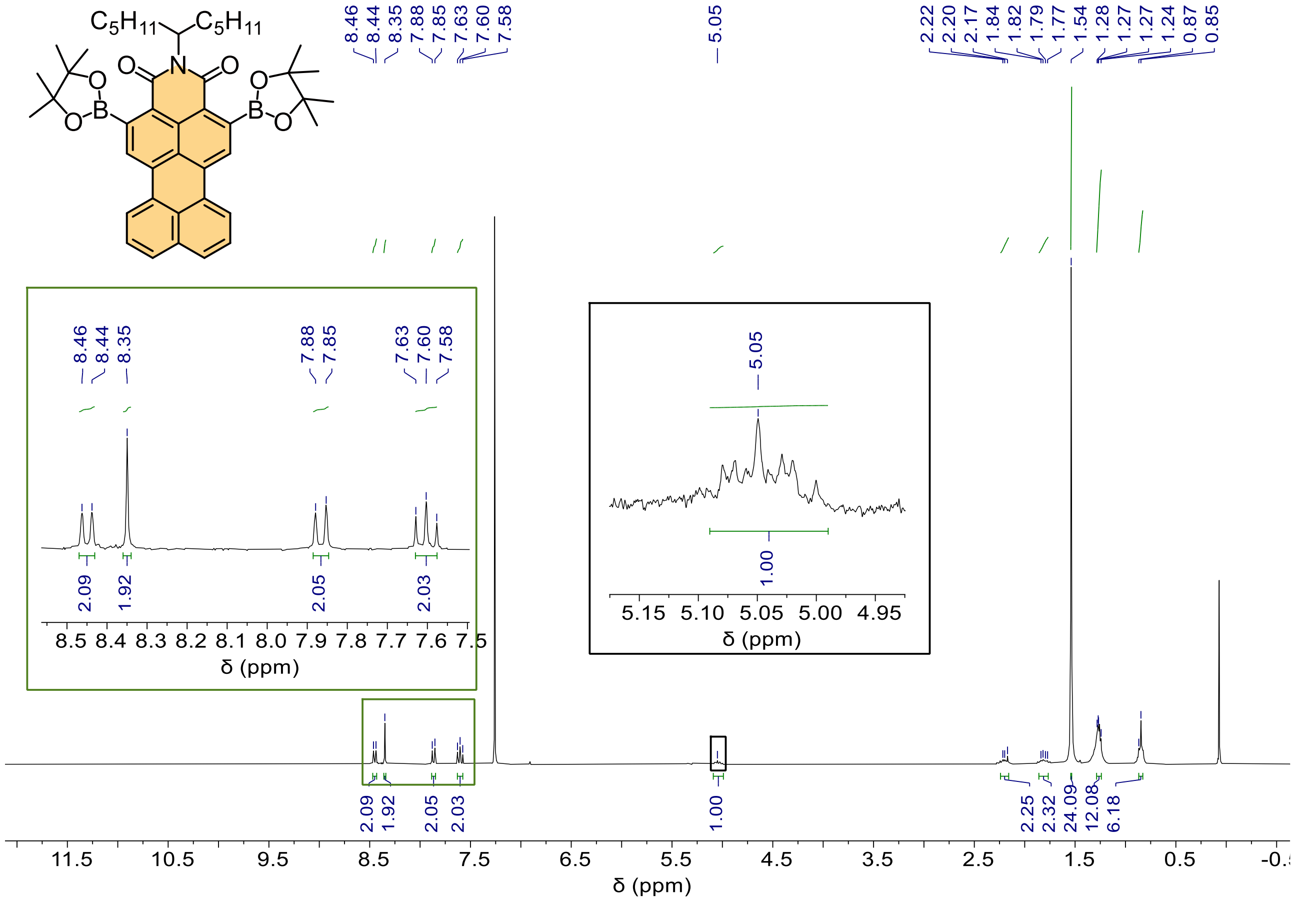

Figure S41 ^{1}H NMR (300 MHz) spectrum of compound **3c** in $CDCl_3$.

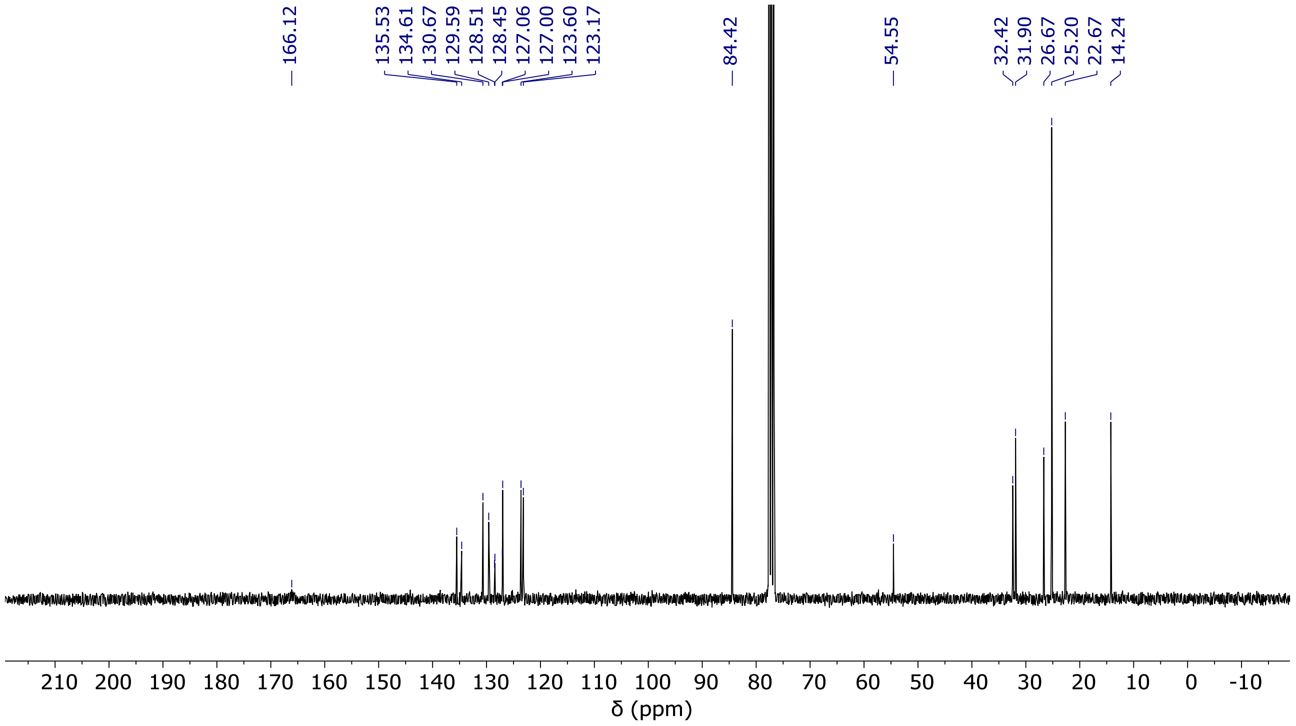

Figure S42 ^{13}C NMR{1H} (126 MHz) spectrum of compound **3c** in $CDCl_3$.

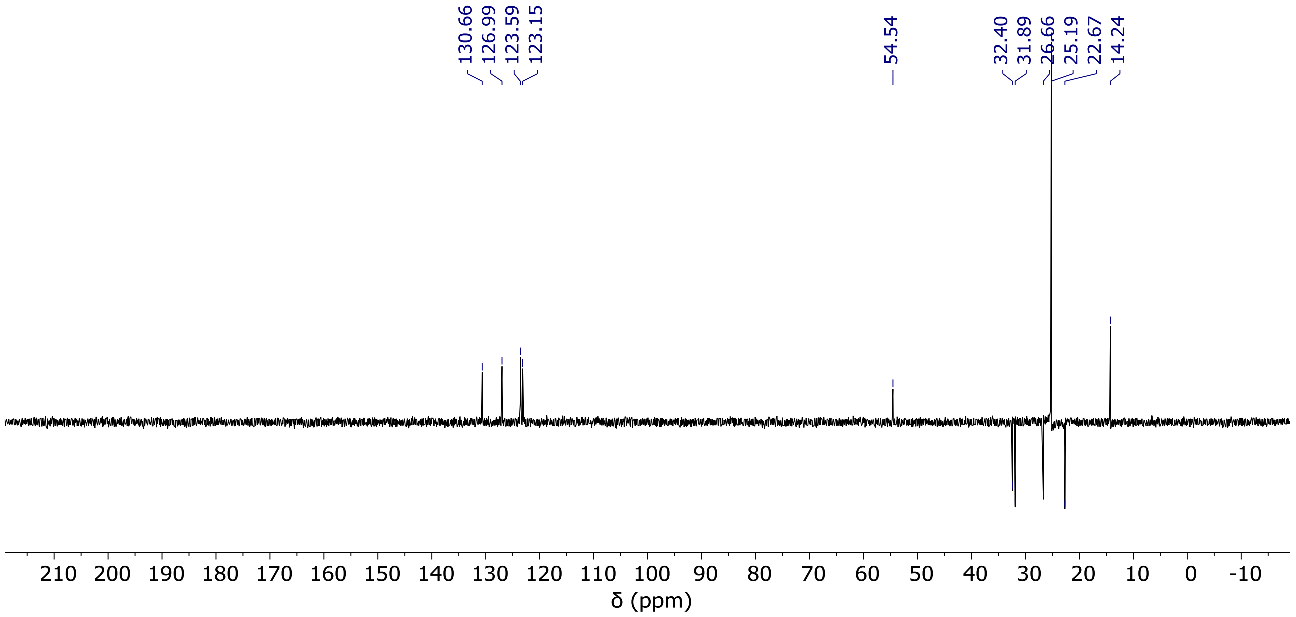

Figure S43 DEPT{1H} (126 MHz) spectrum of compound **3c** in $CDCl_3$.

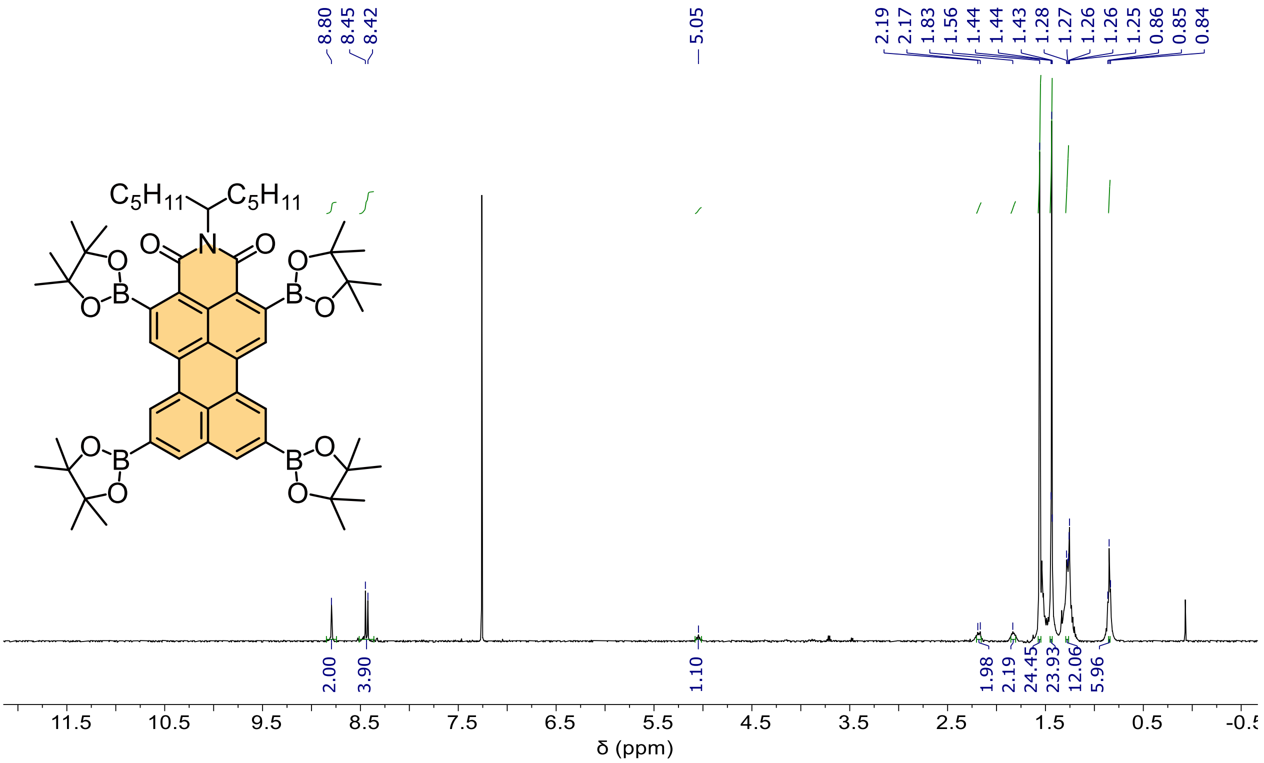

Figure S44 ^{1}H NMR (500 MHz) spectrum of compound **3a** in $CDCl_3$.

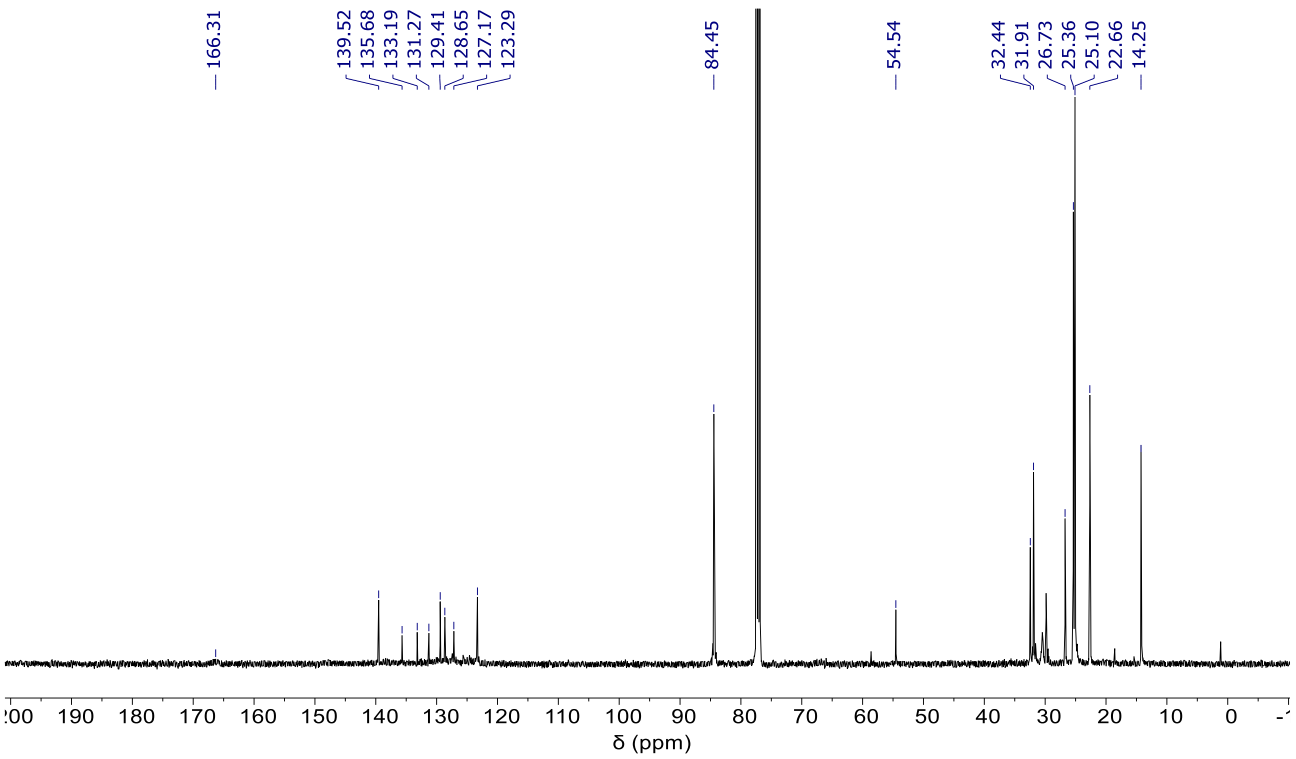

Figure S45 ^{13}C{1H} NMR (126 MHz) spectrum of compound **3a** in $CDCl_3$.

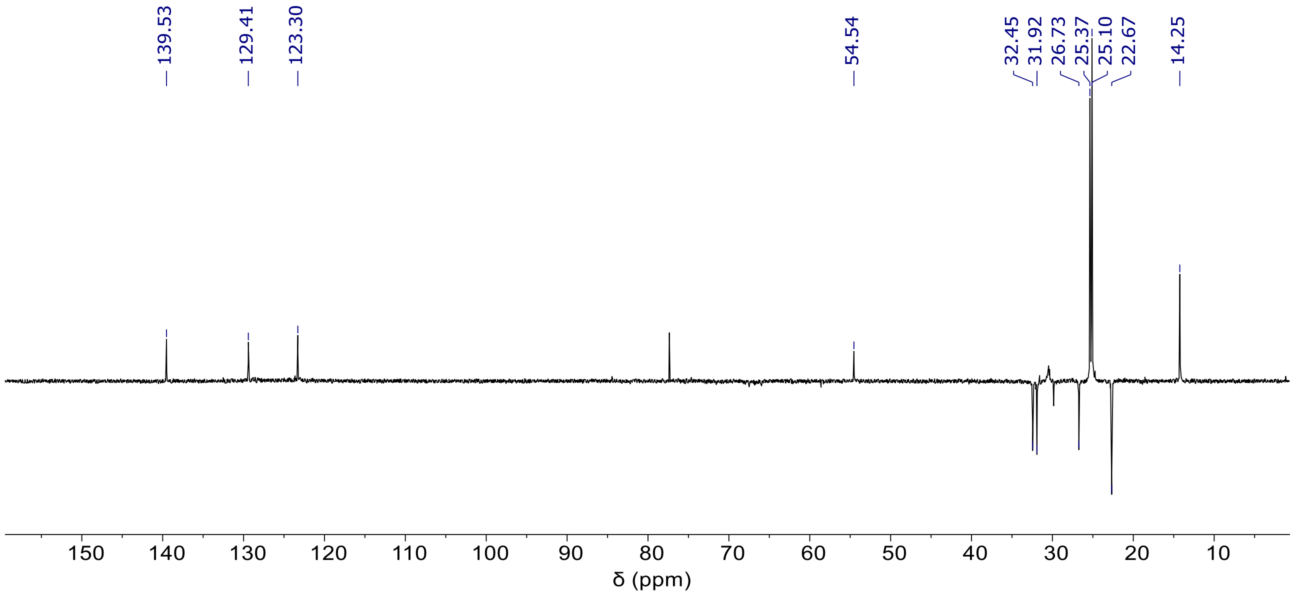

Figure S46 DEPT{1H} (126 MHz) spectrum of compound **3a** in $CDCl_3$.

S.5. HRMS CHARACTERIZATION

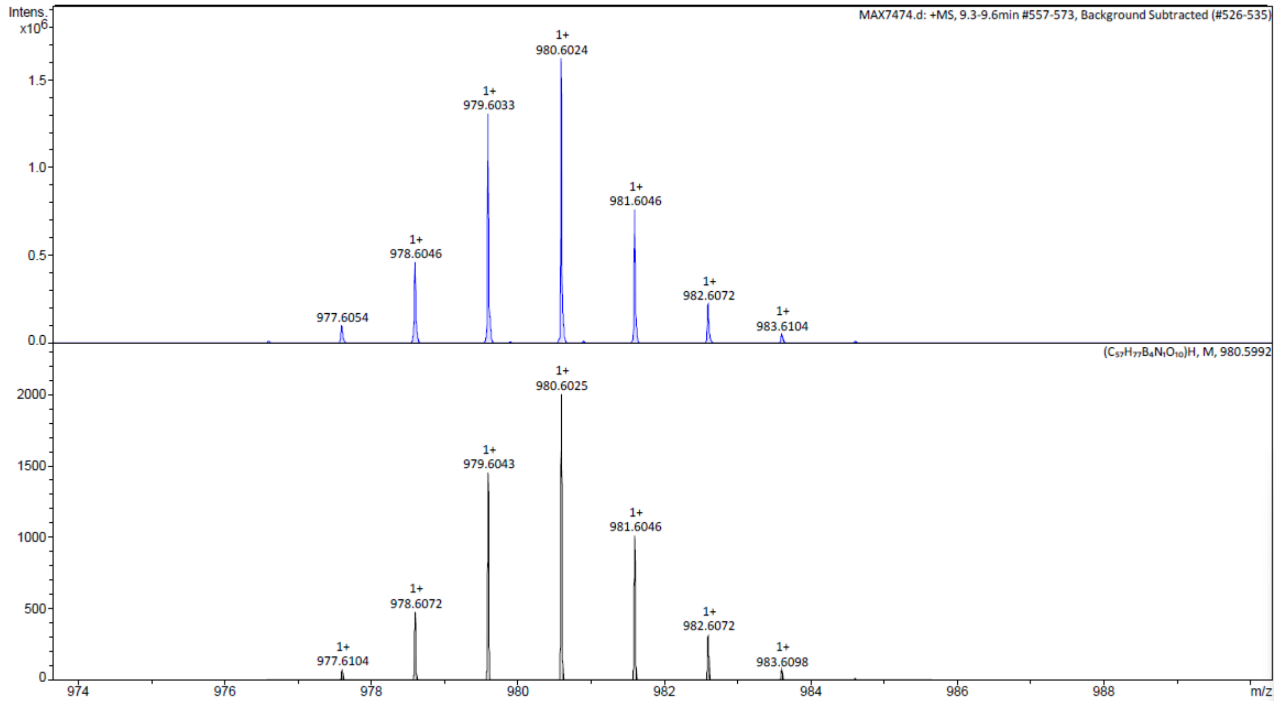

Figure S47 HRMS (APCI) of compound **3a**, calculated (down) vs measured (up).

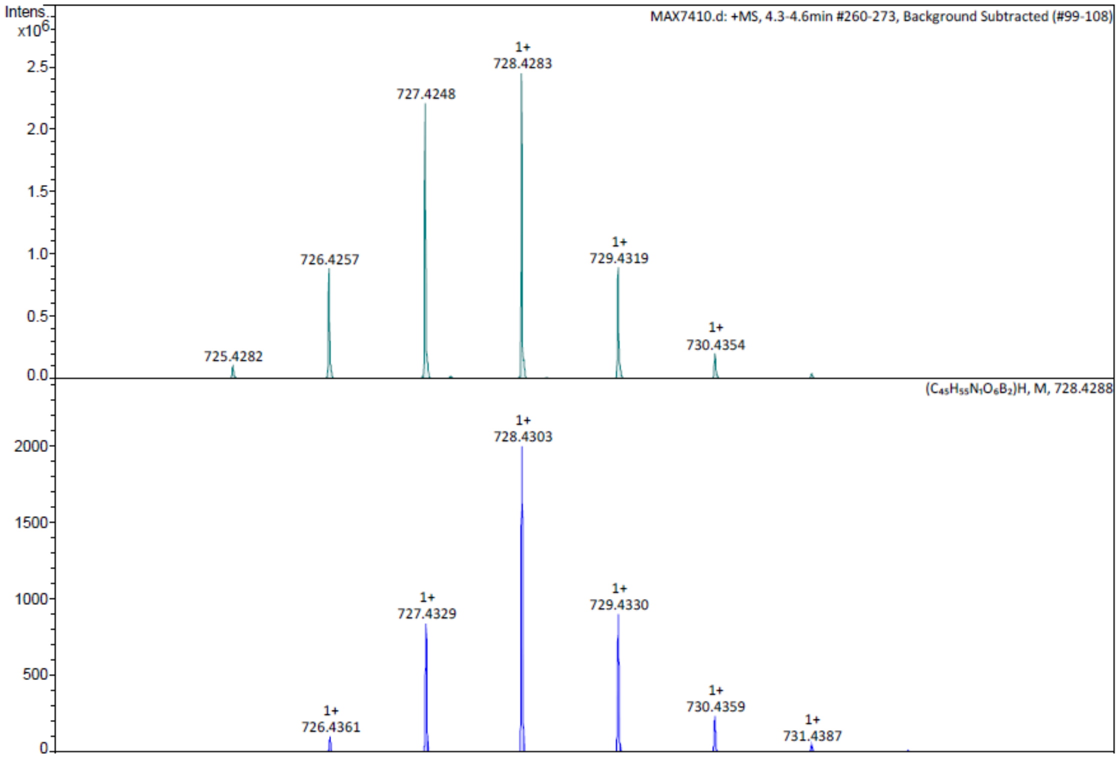

Figure S48 HRMS (APCI) of compound **3b**, calculated (down) vs measured (up).

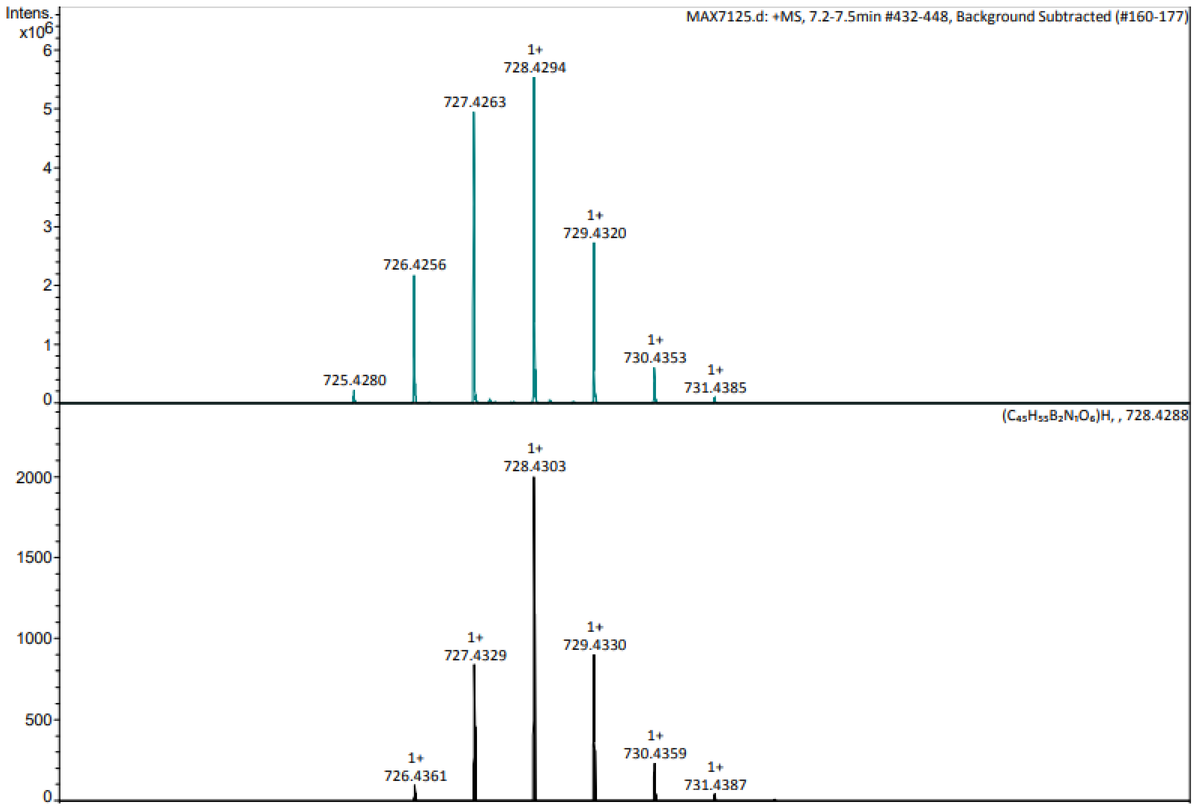

Figure S49 HRMS (APCI) of compound **3c**, calculated (down) vs measured (up).

S.6. SUPPORTING REFERENCES

(S1) Merz, J.; Steffen, A.; Nitsch, J.; Fink, J.; Schürger, C. B.; Friedrich, A.; Krummenacher, I.; Braunschweig, H.; Moos, M.; Mims, D.; Lambert, C.; Marder, T. B. Synthesis, Photophysical and Electronic Properties of Tetra-Donor- or Acceptor-Substituted *Ortho* -Perylenes Displaying Four Reversible Oxidations or Reductions. *Chem. Sci.* **2019**, *10* (32), 7516–7534.

(S2) Battagliarin, G.; Li, C.; Enkelmann, V.; Müllen, K. 2,5,8,11-Tetraboronic Ester Perylenediimides: A Next Generation Building Block for Dye-Stuff Synthesis. *Org. Lett.* **2011**, *13* (12), 3012–3015.

(S3) a) Lee, C.; Yang, W.; Parr, R. G. Development of the Colle-Salvetti Correlation-Energy Formula into a Functional of the Electron Density. *Phys. Rev. B* **1988**, *37* (2), 785–789. b) Becke, A. D. Density-Functional Thermochemistry. III. The Role of Exact Exchange. *The Journal of Chemical Physics* **1993**, *98* (7), 5648–5652. c). Kohn, W.; Becke, A. D.; Parr, R. G. Density Functional Theory of Electronic Structure. *J. Phys. Chem.* **1996**, *100* (31), 12974–12980.

(S4) Gaussian 16, Revision C.01; Frisch, M. J.; Trucks, G. W.; Schlegel, H. B.; Scuseria, G. E.; Robb, M. A.; Cheeseman, J. R.; Scalmani, G.; Barone, V.; Petersson, G. A.; Nakatsuji, H.; Li, X.; Caricato, M.; Marenich, A. V.; Bloino, J.; Janesko, B. G.; Gomperts, R.; Mennucci, B.; Hratchian, H. P.; Ortiz, J. V.; Izmaylov, A. F.; Sonnenberg, J. L.; Williams-Young, D.; Ding, F.; Lipparini, F.; Egidi, F.; Goings, J.; Peng, B.; Petrone, A.; Henderson, T.; Ranasinghe, D.; Zakrzewski, V. G.; Gao, J.; Rega, N.; Zheng, G.; Liang, W.; Hada, M.; Ehara, M.; Toyota, K.; Fukuda, R.; Hasegawa, J.; Ishida, M.; Nakajima, T.; Honda, Y.; Kitao, O.; Nakai, H.; Vreven, T.; Throssell, K.; Montgomery, J. A., Jr.; Peralta, J. E.; Ogliaro, F.; Bearpark, M. J.; Heyd, J. J.; Brothers, E. N.; Kudin, K. N.; Staroverov, V. N.; Keith, T. A.; Kobayashi, R.; Normand, J.; Raghavachari, K.; Rendell, A. P.; Burant, J. C.; Iyengar, S. S.; Tomasi, J.; Cossi, M.; Millam, J. M.; Klene, M.; Adamo, C.; Cammi, R.; Ochterski, J. W.; Martin, R. L.; Morokuma, K.; Farkas, O.; Foresman, J. B.; Fox, D. J. Gaussian, Inc., Wallingford CT, 2016.
