# Supplementary material for: Controlling the Symmetry of Perylene Derivatives via Selective ortho-Borylation
Source: J Org Chem. 2025 Feb 27;90(9):3202–8. doi: 10.1021/acs.joc.4c02669 (PMC12131211; doi:10.1021/acs.joc.4c02669)
Supplement: Supplementary file 1 [file jo4c02669_si_001.zip › FID for Publication/Data.docx]

NMR (Bruker XRD-300 (300 MHz) and/or Bruker XRD-500 (500 MHz)

Analyzed with MestreNova

**Compounds**

1a = JG134 tetra – ^1^H 300 MHz; ^13^C and DEPT 75 MHz.

1b = JG134 bis – ^1^H 300 MHz; ^13^C and DEPT 75 MHz.

2a = DSF2 – ^1^H 300 MHz; ^13^C and DEPT 75 MHz.

2b = JG-135-CAR-1907-MONO – ^1^H 300 MHz; ^13^C and DEPT 75 MHz.

3b = JG131 – DSF27 – ^1^H 500 MHz; ^13^C and DEPT 125 MHz.

3c = DSF16 –^1^H 300 MHz; ^13^C and DEPT 125 MHz.

3a = JG133 – ^1^H 500 MHz; ^13^C and DEPT 125 MHz.
